# Integrin αvβ3 is a Potential Therapeutic Target in Cholangiocarcinoma

**DOI:** 10.7150/ijms.125066

**Published:** 2026-02-04

**Authors:** Fitria Sari Wulandari, Chih-Yang Wang, Dana R Crawford, Yung-Ning Yang, Chee-Kin Then, Sachin Kumar, Fat-Moon Suk, Lin-Yi Huang, Yu-Chen SH Yang, Zi-Lin Li, Ya-Jung Shih, Hoai Tran Tu, Kuan Wang, Hoang Dang Phu, Chun-Mao Lin, Do Thi Minh Xuan, Dahlak Daniel Solomon, Hung-Yun Lin, Jacqueline Whang-Peng

**Affiliations:** 1 Graduate Institute of Cancer Molecular Biology and Drug Discovery, College of Medical Science and Technology, Taipei Medical University, Taipei 11031, Taiwan.; 2 Department of Surgery, Division of Neurosurgery, Shuang Ho Hospital, Taipei Medical University, New Taipei City, Taiwan.; 3 TMU Research Center of Cancer Translational Medicine, Taipei Medical University, Taipei 11031, Taiwan.; 4 Department of Immunology and Microbial Disease, Albany Medical College, Albany, NY 12208, USA.; 5 Department of Pediatrics, E-DA Hospital, I-Shou University, Kaohsiung 82445, Taiwan.; 6 School of Medicine, college of Medicine, I-Shou University, Kaohsiung 82445, Taiwan.; 7 Department of Radiation Oncology, Shuang Ho Hospital, Taipei Medical University, New Taipei City, Taiwan.; 8 Graduate Institute of Clinical Medicine, College of Medicine, Taipei Medical University, Taipei, Taiwan.; 9 Faculty of Applied Sciences and Biotechnology, Shoolini University of Biotechnology and Management Sciences, Himachal Pradesh, 173229, India.; 10 Division of Gastroenterology, Department of Internal Medicine, Wan Fang Hospital, Taipei Medical University, Taipei 116, Taiwan.; 11 Department of Internal Medicine, School of Medicine, College of Medicine, Taipei Medical University, Taipei 110, Taiwan.; 12 Joint Biobank, Office of Human Research, Taipei Medical University, Taipei, Taiwan.; 13 Graduate Institute of Metabolism and Obesity Sciences, College of Nutrition, Taipei Medical University, Taipei 11031, Taiwan.; 14 Graduate Institute of Nanomedicine and Medical Engineering, College of Medical Engineering, Taipei Medical University, Taipei 11031, Taiwan.; 15 Faculty of Chemistry, University of Science, Ho Chi Minh City, Vietnam.; 16 Vietnam National University, Ho Chi Minh City, Vietnam.; 17 Department of Biochemistry and Molecular Cell Biology, Taipei Medical University, Taipei, 110, Taiwan.; 18 Faculty of Pharmacy, Van Lang University, 69/68 Dang Thuy Tram Street, Ward 13, Binh Thanh District, Ho Chi Minh City 70000, Vietnam.; 19 Yogananda School of AI Computers and Data Sciences, Shoolini University, Solan 173229, India.; 20 Cancer Center, Wan Fang Hospital, Taipei Medical University, Taipei 11031, Taiwan.; 21 Traditional Herbal Medicine Research Center of Taipei Medical University Hospital, Taipei Medical University, Taipei 11031, Taiwan.; 22 TMU Research Center of Cancer Translational Medicine and Lung Cancer Research Team, Taipei Medical University, Taipei 11031, Taiwan.

**Keywords:** integrin αvβ3, epidermal growth factor receptor (EGFR), programmed death ligand 1 (PD-L1), thyroxine, epidermal growth factor, nano-tetrac, cholangiocarcinoma

## Abstract

Cell surface receptors play vital roles in cancer growth and metastasis. Integrin αvβ3 is overexpressed in various cancer cells and interacts with different growth factors to stimulate cancer progression. Thyroid hormone binds to αvβ3 to activate signal transduction and cell proliferation. However, thyroxine (T_4_) deaminated analogue, tetraiodothyronine (tetrac), competes for the binding on integrin and inhibits cancer cell growth and metastasis. The current study investigated the pathogenic role of integrin αvβ3 and the potential of a novel therapeutic strategy targeted to integrin αvβ3. Pathogenetic studies of clinical samples revealed integrin αvβ3 cross-talked with EGFR and downstream signal transduction networks affected by thyroid hormone and EGF related to the progression of cholangiocarcinoma malignancy. Thyroxine and EGF stimulated PD-Ligand 1 (PD-L1) expression and cancer growth in cholangiocarcinoma. The thyroxine-induced PD-L1 accumulated in the nuclei and colocalized with p300. Alternatively, EGF increased cytosolic PD-L1 and nuclear accumulation of β-catenin. Targeting integrin αvβ3 with lipo-tetrac and its Dox-derivative induced anti-proliferation *in vitro* and in the xenografted animal model. Our research provides a fundamental understanding of the therapeutic role of integrin αvβ3 and the potential therapeutic approach in cholangiocarcinoma treatment.

## Introduction

Cholangiocarcinoma (CCA) is a highly aggressive malignancy arising from the biliary tract epithelium and has become increasingly recognized worldwide for its poor clinical outcome. CCA accounts for about 3 percent of all gastrointestinal cancers and 10-20 percent of primary liver cancer 1, 2. The incidence of cholangiocarcinoma has been rising globally, with the highest rates observed in Southeast Asia, where the disease often results from chronic infection with *Opisthorchis viverrini* and *Clonorchis sinensis*, parasites known to cause biliary tract inflammation 3. However, in Western countries, the disease is mainly associated with primary sclerosing cholangitis, hepatitis B and C virus infections, and liver cirrhosis 3, 4. Clinically, CCA is classified into three anatomical subtypes: intrahepatic (iCCA), perihilar (pCCA), and distal (dCCA). Perihilar tumors are the most common, representing 50-60 percent of all cases, followed by distal CCA at 20-30 percent and intrahepatic CCA at roughly 10 percent 5, 6. These subtypes differ in their cell of origin, stromal architecture, immune composition, and dominant signaling pathways, which has important implications for biomarker expression and therapeutic targeting 7. Despite advances in imaging techniques and surgical approaches, cholangiocarcinoma is typically diagnosed at an advanced stage when surgical resection is no longer feasible. The prognosis remains poor, with a 5-year survival rate of less than 10% for patients diagnosed with advanced disease and high rates of recurrence 1, 8. The lack of effective targeted therapies and the resistance to chemotherapy further complicate treatment outcomes.

Integrins such as integrin αvβ3 are increasingly recognized as critical molecules in tumor progression. Integrin αvβ3 is a cell surface receptor involved in cell adhesion, migration, and invasion 9. It mediates interactions between cells and the extracellular matrix, facilitating tumor cell attachment and migration. Integrin αvβ3 has been implicated in tumor metastasis, and its expression correlates with poor prognosis in various cancers 10. In cholangiocarcinoma, integrin αvβ3 has been shown to contribute to the invasion and migration of cancer cells, enhancing the potential for metastasis 11. The thyroid hormone signaling pathway has a wide range of functions in terms of individual development, maintenance of homeostasis, cell proliferation and differentiation, and glucose metabolism. In addition to thyroid nuclear receptor, triiodothyronine (T_3_) and L-thyroxine (T_4_) can bind to integrin αvβ3 and stimulate cancer cell growth and metastasis 12. It shows the relevant functions of various cancers by stimulating the extracellular signal-regulated kinase 1/2 (ERK1/2) pathways responsible for tumorigenesis 12.

In addition to integrin αvβ3, EGFR (epidermal growth factor receptor) is a key player in the progression of cholangiocarcinoma. This receptor tyrosine kinase is overexpressed in various cancers, including cholangiocarcinoma 13. EGFR regulates numerous cellular processes, including cell proliferation, survival, migration, and invasion, by activating downstream signaling pathways such as RAS/RAF/MEK/ERK, PI3K/AKT, and JAK/STAT 14. EGFR activation plays a crucial role in driving tumor progression by promoting cell division and survival, and its inhibition has been shown to reduce tumor growth and metastasis in preclinical models 15. In cholangiocarcinoma, EGFR overexpression has been associated with poor prognosis, and targeting EGFR has emerged as a potential therapeutic strategy 16. Moreover, integrins have been shown to cooperate with EGFR in promoting tumor progression and immune modulation. It has been well-established that thyroxine stimulates EGFR-dependent signal transduction via integrin αvβ3 12, suggesting that targeting both molecules simultaneously could provide a more effective therapeutic strategy 11.

Recent studies have highlighted the importance of immune evasion mechanisms in the progression of many cancers, including cholangiocarcinoma. Immune checkpoint molecules, such as PD-L1 (programmed death-ligand 1), have gained significant attention due to their enabling tumors to evade immune surveillance. PD-L1 expression is upregulated in many cancers, including cholangiocarcinoma 17-19. It plays a pivotal role in immune suppression by binding to its receptor PD-1 on T cells. This interaction leads to T cell exhaustion, a potentially permanent dysfunction characterized by reduced or absent T cell effector function, lack of response to stimuli, and altered transcriptional and epigenetic state 19. However, the molecular factors regulating PD-L1 expression in cholangiocarcinoma remain poorly understood, making it an important target for further investigation.

One of the hallmarks of cholangiocarcinoma is its ability to evade the immune system, a phenomenon known as immune escape 20. Tumor cells often exploit various immune checkpoint pathways to suppress the body's natural immune response. The interaction between EGFR, integrins, and PD-L1 is particularly interesting, as these molecules may work together to modulate both the tumor microenvironment and the immune landscape 21. EGFR activation can induce PD-L1 expression in tumor cells, while integrins may contribute to immune cell recruitment and immune suppression 21, 22. The exact mechanisms by which EGFR and integrin αvβ3 regulate PD-L1 expression and immune cell infiltration in cholangiocarcinoma remain unclear, and understanding these interactions is of utmost importance for developing targeted therapies to overcome immune evasion. Given the aggressive nature of this disease, developing novel therapeutic strategies targeting the underlying molecular mechanisms is crucial 1, 8. However, the interplay between EGFR and other molecular markers, such as PD-L1, and their combined impact on immune modulation in cholangiocarcinoma remains unclear.

Crosstalk between signal transduction pathways has been well-described in cancer pathology. It has also developed combination therapies to block different signal transduction pathways. Recently, we have shown that targeting integrin αvβ3 by 3 3' 5 5'-tetraiodothyroacetic acid and its nano-particulate derivatives (NDAT) can inhibit cancer growth *in vitro* and *in vivo* in EGFR-mutant cancer cell lines 12. Modulating integrin αvβ3-dependent signal transduction by nano-tetrac suppresses activations of PI3K, ERK1/2, and PD-L1 expression sequentially. Consequently, nano-tetrac inhibits cancer proliferation *in vitro* and *in vivo* 12.

**Figure 1:**
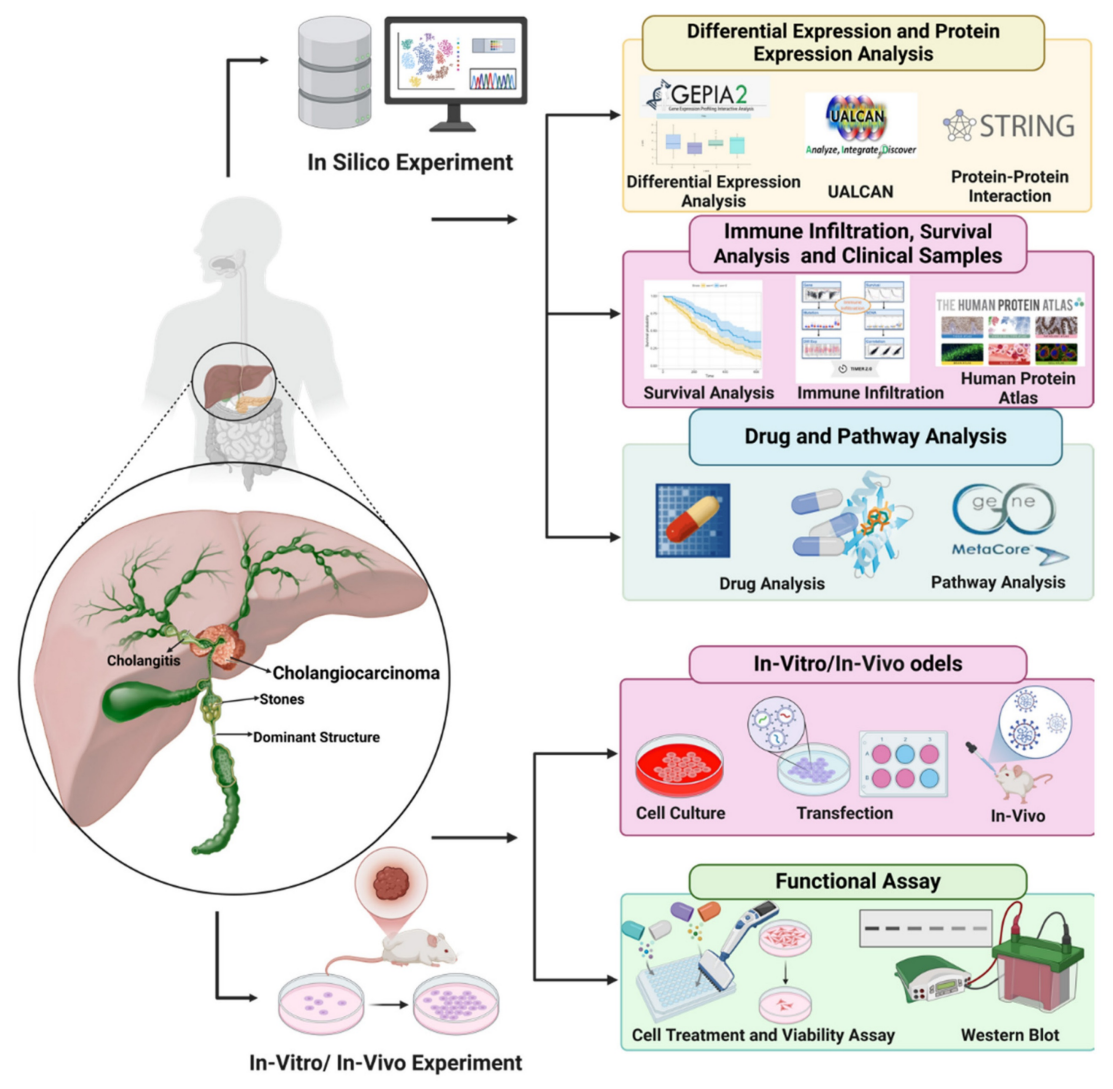
Schematic diagram depicting the experimental workflow employed in this study to investigate cholangiocarcinoma and its molecular mechanisms. The schematic diagram illustrates the integrated in silico, *in vitro*, and *in vivo* approaches used in this study. Computational analyses were conducted to examine differential gene expression, protein interactions, immune infiltration, and survival correlations using tools such as GEPIA2, UALCAN, STRING, and the Human Protein Atlas. Drug sensitivity, molecular docking, and pathway analysis were performed using GDSC2 and MetaCore to explore potential therapeutic targets. *In vitro* experiments included cell culture, transfection, and functional assays, such as cell viability and Western blot analysis, to validate computational findings and investigate key molecular mechanisms in cholangiocarcinoma.

In this study, we aimed to analyze the pathogenic role of integrin αvβ3 and its correlations with EGFR and PD-L1 in cholangiocarcinoma. Combining bioinformatic analysis, cell line experiments, and clinical biopsy validation, we validated the integrin αvβ3 antagonist, nano-tetrac derivative-induced anti-proliferation in cholangiocarcinoma xenograft modeling. The flowchart of our study, as illustrated in **Fig. 1**, represents a significant step forward in our understanding of cholangiocarcinoma. We aimed to understand how integrin αvβ3 and EGFR regulate PD-L1 expression and immune modulation in cholangiocarcinoma to identify new therapeutic strategies that target these molecular pathways. By bridging bioinformatic insights with experimental validation, our findings may provide new avenues for targeted therapies that can significantly improve the prognosis of cholangiocarcinoma patients. We also validated the hypothesis of the stimulating effect of thyroid hormone and EGF in cholangiocarcinoma, providing reassurance about the reliability of our findings. Finally, we verified the anti-cancer growth of nano-tetrac derivatives effectively in xenograft modeling, further underlining the potential impact of our study.

## Materials and Methods

### Cell Cultures

Human bile duct carcinoma TFK-1 was obtained from the American Type Culture Collection (Gaithersburg and Germantown, MD). KRAS wild-type SSP-25 and KRAS mutant HuCCT1 were obtained from Riken Bioresource Research Center (Tsukuba, Japan) and were authenticated by a next-generation sequencing (NGS) analysis. Based on the NGS analysis, results indicated that the intrahepatic cholangiocarcinoma, SSP-25 cell is ETK-1:TP53; Simple; p.Arg175His (c.524G>A), which correlated with the results shown on the website. Cells were cultured in RPMI-1640 medium supplemented with 10% fetal bovine serum in a humidified incubator with 5% CO_2_ at 37 ºC.

### DL-N2 and Integrin αvβ3 Binding Characteristics

DL-N2 is a nanoparticulate formulation of tetraiodothyroacetic acid (tetrac) designed to target the thyroid hormone receptor site on integrin αvβ3. Structural and biochemical studies show that this receptor pocket is formed at the interface between the αv S1 domain and the β3 I-like domain, indicating that effective binding requires the intact αvβ3 heterodimer. DL-N2 does not interact with αv or β3 individually. Functional loss-of-activity experiments further demonstrate that β3 plays the dominant role in transmitting tetrac-dependent inhibitory signaling, whereas αv knockdown produces only a partial reduction in response. Through this interaction, DL-N2 blocks T_4_-mediated activation of FAK/SRC, PI3K, ERK1/2, and STAT3 pathways, thereby suppressing integrin-dependent tumor growth and enhancing the effects of EGFR-targeted therapy 23-25.

### Bioinformatic Analysis

Bioinformatic analysis was carried out to investigate the expression and correlation of key genes in cholangiocarcinoma using publicly available datasets. Bioinformatics tools such as GEPIA2 and UALCAN were used to perform differential expression analysis, survival analysis, and clinical relevance studies 26, 27. GEPIA2 is a comprehensive web tool for analyzing gene expression across various types of cancer using RNA-seq data. We utilized this tool to analyze the differential expression of integrin αvβ3, EGFR, and PD-L1 in different cancer types, specifically focusing on cholangiocarcinoma. The tools allow for the visualization of gene expression data and comparing expression patterns between tumor and normal tissues. UALCAN is another powerful bioinformatic tool that we used for clinical analysis. It integrates large-scale cancer data to study the expression of genes in various cancer types. We specifically employed UALCAN to assess the relationship between EGFR, integrin αvβ3, and PD-L1 expression in cholangiocarcinoma and their relevance to patient survival. STRING databases were used to explore the interactions between EGFR, PD-L1, and integrin αvβ3 for protein-protein interaction (PPI) network analysis 28. STRING allowed us to visualize the interactions between these genes and identify potential signaling pathways involved in cholangiocarcinoma progression. In addition to the interaction analysis, we used Metacore, Gene Ontology (GO) and Kyoto Encyclopedia of Genes and Genomes (KEGG) pathway analysis to investigate the biological functions and signaling pathways associated with EGFR and integrin αvβ3 29.

Single-cell RNA-seq data for cholangiocarcinoma was analyzed using the publicly available dataset GSE142784, which was downloaded as a pre-processed and annotated object from the TISCH2 portal 30. The dataset contained curated major and minor cell-type labels, including malignant status, fibroblast subtypes, endothelial cells, immune subsets, and myeloid lineages. The expression matrix and metadata were imported into Seurat (v5.0) for downstream analysis 31. Following log-normalization, highly variable genes were identified using the default selection method. Principal component analysis (PCA) was performed, and the first 20 principal components were used to compute the shared nearest neighbor graph. Uniform manifold approximation and projection (UMAP) was then carried out to generate two-dimensional embeddings and cluster separations. Cell clustering was conducted using the standard Seurat workflow with the default resolution parameter, after which the original TISCH2 annotations were mapped back onto the Seurat-generated clusters to ensure biological consistency.

To examine gene-level expression patterns, feature plots and violin plots were generated for ITGAV, ITGB3, EGFR, and CD274, enabling visualization of their distribution across malignant, stromal, and immune populations. For intercellular communication analysis, the annotated Seurat object was converted for use in CellChat (v1.6), and ligand-receptor modeling was performed using the built-in human interaction database. Signaling probabilities, interaction strengths, and compartment-level communication patterns were computed using the default parameters. All plots, including UMAP projections, violin plots, and communication networks, were generated using the SingleCellPipeline (SCP) package, which provided unified visualization formatting and ensured reproducible figure generation.

### Cell Viability Assay

SSP-25 and HuCCT1 cells were seeded in 96-well plates at a 3000 cells/well density. After 24 h for cell attachment, cells were starved with 0.25% hormone-depleted serum-supplemented medium for 24 h. Then, serum-starved cells were treated with various concentrations of EGF (cat. no.: E9644, Sigma-Aldrich, St. Louis, MO, USA), or T_4_ (cat. no.: SI-T2376, Sigma-Aldrich) at varying concentrations and treatment times according to the experimental design. Cells that did not receive EGF or T_4_ were treated with phosphate-buffered saline (PBS) or KOH-PG buffer, respectively. Medium and reagents were refreshed daily. Cell viability was assayed with an alamar blue Cell Viability Reagent (cat. no.: DAL1025, Thermo Scientific, Waltham, MA, USA), according to the manufacturer's instructions.

### Western Blotting

SSP-25 and HuCCT1 cells were seeded in 6 cm petri dishes. Cells were starved with 0.25% hormone-depleted serum-supplemented medium for 24 h. Cells were treated with different agents for 24 h before harvest. After cells were harvested, proteins were extracted according to the research design. Protein samples were resolved by 10% sodium dodecyl-sulfate polyacrylamide gel electrophoresis (SDS-PAGE). A 10 μg quantity of protein was loaded into each well with sample buffer, and samples were resolved by electrophoresis at 100 V for 2 h. The resolved proteins were transferred from the polyacrylamide gel to Millipore Immobilon-PSQ Transfer polyvinylidene difluoride (PVDF) membranes (Millipore, Billerica, MA, USA) with the Mini Trans-Blot® Cell (Bio-Rad Laboratories). Membranes were blocked with a solution of 2% bovine serum albumin in Tris-buffered saline (TBS) with 0.1% Tween-20 (TBST) and incubated with primary antibodies to PD-L1 (cat. no.: GTX104763, GeneTex International, Hsinchu City, Taiwan) at a 1:1000 dilution, AKT (cat. no.: 60203, Proteintech) at a 1:1000 dilution, p-AKT (Ser473) (cat. no.: 9271, Cell Signaling Technology) at a 1:1000 dilution, STAT3 (cat. no.: 610190, BD Biosciences) at a 1:1000 dilution, p-STAT3 (Tyr705) (cat. no.: 9145, Cell Signaling Technology) at a 1:1000 dilution, p-STAT3 (Ser727) (cat. no.: 9136, Cell Signaling Technology) at a 1:1000 dilution, non-p-β-catenin (Ser33/Ser37/Tyr41) (cat. no.: 8814, Cell Signaling Technology) at a 1:1000 dilution, p-β-catenin (Ser33/Ser37/Tyr41) (cat. no.: 9561, Cell Signaling Technology) at a 1:1000 dilution, β-catenin (cat. no.: 610153, BD Biosciences, San Diego, CA, USA) at a 1:1000 dilution, lamin B1 (cat. no.: GTX103292, GeneTex International) at a 1:1000 dilution, and glyceraldehyde-3-phosphate dehydrogenase (GAPDH) (cat. no.: 60004-1, GeneTex International) at a 1:10,000 dilution at 4 °C overnight. The antibody-probed membrane was washed with TBST containing 5% fat-free milk (5% TBST/milk) three times for 10 min and then probed with goat anti-mouse immunoglobulin G (IgG) (cat no.: GTX213111-05, GeneTex International) or goat anti-rabbit IgG (cat. no.: GTX213110-04, GeneTex International) horseradish peroxidase (HRP)-conjugated secondary antibodies, which were prepared in 5% TBST/milk at a 1:10,000 dilution at room temperature for 1 h. After the membrane was washed three times for 10 min with TBS, chemiluminescent detection was performed using the Immobilon Western Chemiluminescent HRP Substrate (Millipore, Billerica, MA, USA). Bands were imaged with the BioSpectrum Imaging System (UVP, Upland, CA, USA) and quantified using densitometry by ImageJ 1.47 software (National Institute of Health, Bethesda, MD, USA) according to the software instructions.

### Animal Study Design

All animal experimental procedures were approved by the Institutional Animal Care and Use Committee of the National Defense Medical Center, Taipei, Taiwan (IACUC, LAC-2020-0471). 6-week-old NOD SCID mice were purchased from the National Laboratory Animal Center (Taipei, Taiwan), housed in a reserved, pathogen-free facility and acclimated to the nursery for one week before the experiment. For xenograft implantation, mice were anesthetized with 3 % isoflurane and subcutaneously inoculated with aliquots of 1 × 10^7^ TFK-1 cells/100 μl 50 % Matrigel (BD Matrigel™ Basement Membrane Matrix). Seven days post-implantation, the tumor status was assessed, and the mice were randomly divided into distinct experimental groups. After two weeks, when the tumor grew bigger than 100 mm^3^, mice received intravenous injections of either solvent (PBS), DL-N2 (tetrac 0.1 mg/kg), Lipo-Dox (Dox 2 mg/kg), or DL-N2-Dox (tetrac 0.1 mg/kg and Dox 2 mg/kg) by one dose per week for four weeks. The tumor sizes will be measured every three days by caliper and calculated by formula V = (d× D × L)/2. The change in tumor volume was calculated by dividing the final measured volume by the initial measured volume. After four weeks of treatment, all animals were sacrificed, the tumor weight was measured and analyzed.

### Statistical Analysis

In this study, the statistical significance of all data was analyzed by a two-tailed Student's t-test using Excel. All data are presented as the mean ± standard deviation (SD) or standard error of the mean (SEM). p < 0.05 (*), and p < 0.01 (**) were considered statistically significant.

## Results

### Bioinformatic Analyses Reveal Integrin αvβ3-EGFR-PD-L1 Axis in Cholangiocarcinoma

Targeting integrin αvβ3 with thyroxine deaminated analogues or its nano-particulate can inhibit cancer growth *in vitro* and *in vivo*. They also induced anti-cancer growth in *KRAS* mutant EGFR antagonist gefitinib-resistant cancer cell lines 32. These observations raised our interest in investigating the key role of integrin αvβ3 in cholangiocarcinoma and its correlations with EGFR in pathogenic effects in cholangiocarcinoma. To comprehensively explore the molecular dynamics of integrin αvβ3 in cholangiocarcinoma, we began with bioinformatic studies that integrated various publicly available cancer datasets. The central focus of this study was the analysis of integrin αvβ3 and its network, especially EGFR. They are key surface biomarkers implicated in cancer progression and metastasis. Integrin αvβ3 is a critical molecule involved in cell adhesion, migration, and extracellular matrix interactions, facilitating tumor invasion in various types of cancer. On the other hand, EGFR is well-known for its role in cell signaling pathways, such as ERK, AKT, and PI3K, which are crucial for tumor survival and proliferation. Furthermore, studies demonstrate that integrin αvβ3 crosstalks with EGFR for cancer progression.

**Figure 2:**
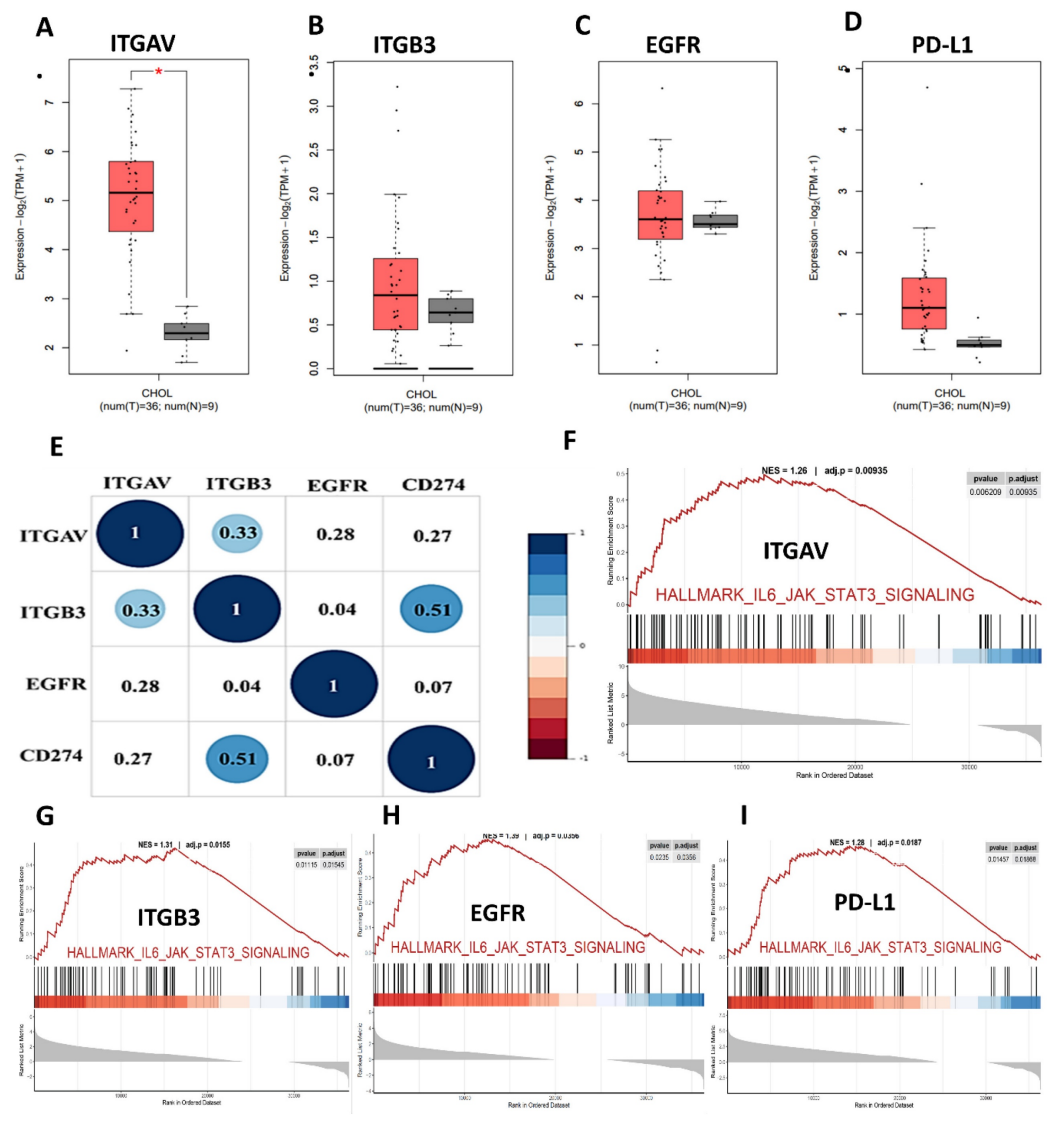
Expression and pathway analysis of ITGAV, ITGB3, EGFR, and PD-L1 in cholangiocarcinoma (CHOL). (A-D) Box plots showing gene expression levels (log₂ TPM + 1) of ITGAV, ITGB3, EGFR, and PD-L1 in CHOL tumor tissues (T, n = 36) versus normal tissues (N, n = 9) from TCGA data. Tumor samples show significantly elevated expression compared to normal controls, with statistical significance denoted by asterisks (*p < 0.05). (E) Correlation matrix (circle plot) displaying positive correlations among ITGAV, ITGB3, CD274 (PD-L1), and EGFR, highlighting potential co-expression and coordinated regulation in cholangiocarcinoma pathogenesis. (F-I) Gene Set Enrichment Analysis (GSEA) plots showing enrichment of the IL6-JAK-STAT3 signaling pathway in samples with high expression of the respective genes, indicating their involvement in inflammatory and tumor-promoting pathways.

To elucidate the oncogenic and immunomodulatory landscape of cholangiocarcinoma (CHOL), we conducted a series of bioinformatic analyses focusing on integrin αvβ3 (comprising ITGAV and ITGB3), EGFR, and PD-L1 (CD274). Differential gene expression analysis from publicly available datasets revealed that ITGAV, ITGB3, EGFR, and PD-L1 were all significantly upregulated in CHOL tumor tissues compared to normal bile duct tissues **(Fig. 2A-D)**. ITGAV expression was markedly higher in tumors, aligning with its established role in facilitating extracellular matrix interactions and tumor invasion **(Fig. 2A)**. ITGB3 expression was notably elevated **(Fig. 2B)**. In parallel, EGFR, a known driver of cell proliferation and survival, also showed strong upregulation **(Fig. 2C)**, highlighting its involvement in promoting tumor cell adhesion and metastasis. Furthermore, the immune checkpoint molecule PD-L1 was significantly overexpressed **(Fig. 2D)**, indicating the potential for immune escape mechanisms in CHOL.

Correlational analysis revealed significant positive associations between ITGAV, ITGB3, EGFR, and PD-L1 expression levels **(Fig. 2E)**. For example, ITGAV expression correlated with both EGFR (r = 0.31) and PD-L1 (r = 0.31). ITGB3 also showed modest correlations with EGFR (r = 0.28) and PD-L1 (r = 0.27). These findings suggest that these molecules may operate in a coordinated network contributing to CHOL pathogenesis. The co-expression and pathway convergence underscore the potential of targeting the integrin αvβ3-EGFR-PD-L1 axis as a therapeutic strategy in cholangiocarcinoma. To further understand the functional implications of these molecular alterations, we performed Gene Set Enrichment Analysis (GSEA) on samples with high expression of the above markers. The analyses showed robust enrichment of the IL6/JAK/STAT3 signaling and IFN-γ response pathways **(Fig. 2F-I)**, which are known to contribute to tumor immune modulation and inflammation. Notably, STAT3 activation has been linked to the transcriptional regulation of PD-L1, suggesting a possible mechanistic link among integrin signaling, EGFR activation, and immune checkpoint expression.

Moreover, PD-L1, a well-known immune checkpoint molecule, was also highly expressed in cholangiocarcinoma samples, correlating with the tumor's potential to evade immune detection. *ITGAV* expression shows differential expression, with increased levels in specific cancers like breast and gastric cancer. *ITGB3* also follows a similar pattern, indicating its involvement in tumor progression and metastasis. *EGFR* expression is significantly higher in various cancers, with prominent upregulation observed in tumors such as lung and colon cancers 33. However, *PD-L1* expression is notably elevated across multiple tumor types, suggesting its role in immune escape mechanisms 34. These initial findings underscore the importance of integrin αvβ3, EGFR, and PD-L1 as key players in the pathogenesis of cholangiocarcinoma. The upregulation of these molecules in cholangiocarcinoma supports their role in immune evasion and suggests that targeting them could be a promising therapeutic strategy.

### Correlation between Integrin αvβ3 Expression and Key Genes in Cholangiocarcinoma

**Figure 3:**
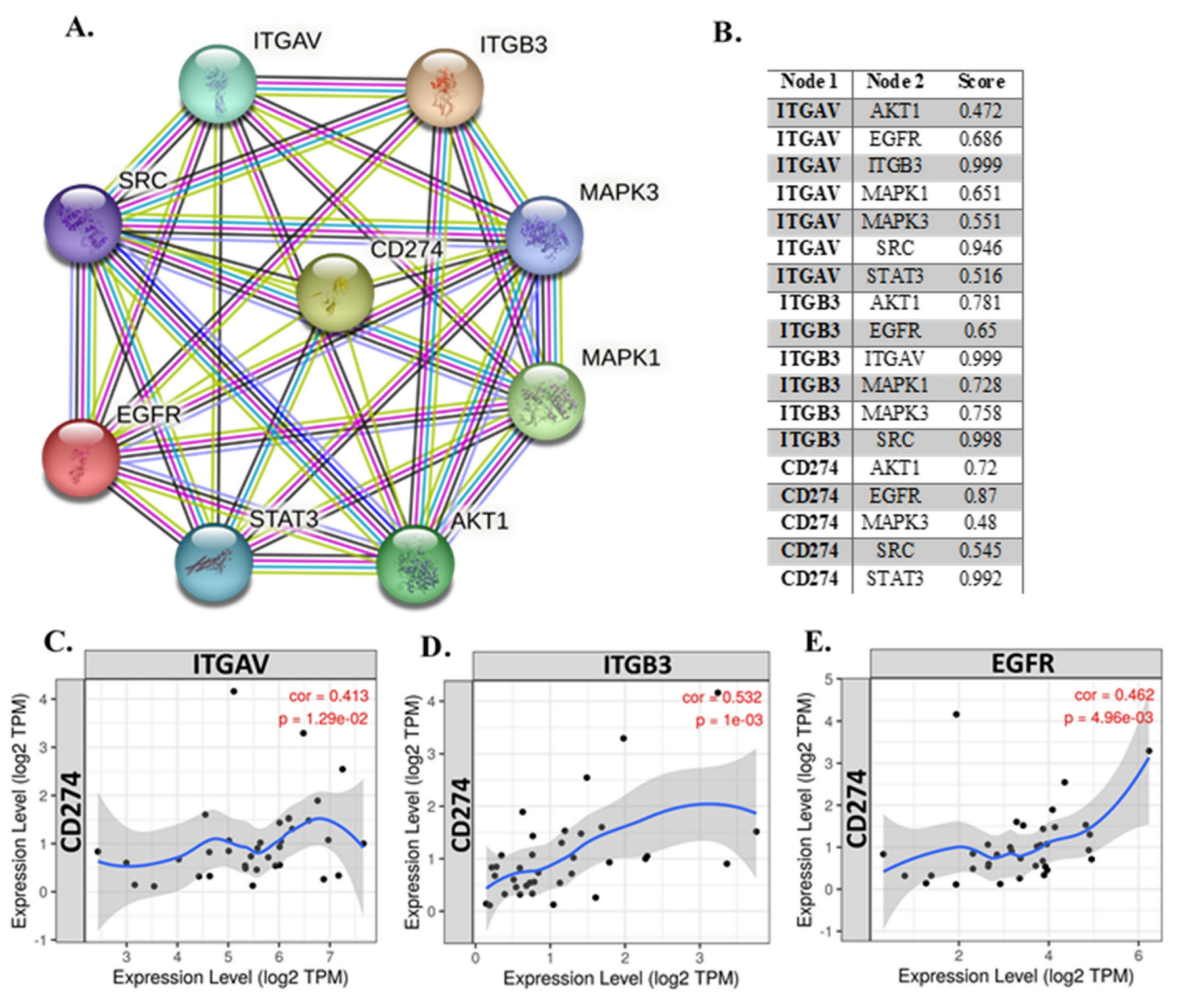
Integrated correlation and interaction analysis of ITGAV, ITGB3, EGFR, and CD274 (PD-L1) in cholangiocarcinoma. (A) Protein-protein interaction (PPI) network constructed using the STRING database, depicting the interactions among key genes: ITGAV, ITGB3, EGFR, CD274, SRC, STAT3, MAPK1, MAPK3, and AKT1. Edge colors represent known or predicted interactions, highlighting the central role of EGFR and CD274 in immune regulation and oncogenic signaling. (B) Tabulated STRING correlation scores between select gene pairs, showing strong co-associations such as ITGAV-ITGB3 (0.999), ITGB3-SRC (0.998), and CD274-EGFR (0.87), suggesting functional interplay in CHOL progression. (C-E) Expression correlation scatter plots between CD274 (PD-L1) and ITGAV (C), ITGB3 (D), and EGFR (E) in cholangiocarcinoma (TCGA dataset). All three genes show significant positive correlations with CD274: ITGAV (cor = 0.413, p = 1.29e-02), ITGB3 (cor = 0.532, p = 1e-03), and EGFR (cor = 0.462, p = 4.96e-03), supporting potential co-regulation and immune checkpoint association.

To explore the relationship between integrin αvβ3 (composed of ITGAV and ITGB3) and other key genes implicated in cholangiocarcinoma, we performed both correlation analysis and protein-protein interaction (PPI) network mapping **(Fig. 3)**. The PPI network **(Fig. 3A)** revealed extensive interactions among integrin subunits (ITGAV and ITGB3), EGFR, CD274 (PD-L1), and major signaling molecules such as AKT1, SRC, STAT3, MAPK1, and MAPK3. Notably, EGFR emerged as a central hub in the network, displaying strong associations with ITGAV, ITGB3, and PD-L1. This suggests that EGFR may coordinate tumor-promoting signaling events such as cell adhesion, migration, and immune evasion. The STRING interaction confidence scores **(Fig. 3B)** further confirmed these associations, with particularly high scores for ITGAV-ITGB3 (0.999), ITGB3-SRC (0.998), ITGB3-MAPK1 (0.728), and CD274-EGFR (0.87), highlighting their potential co-regulatory roles in cholangiocarcinoma pathogenesis.

We next examined whether these protein-level interactions are mirrored at the transcriptional level. Correlation analysis of gene expression **(Fig. 3C-E)** revealed that ITGAV expression positively correlated with PD-L1 (CD274) (R = 0.413, p = 1.29e-02; **Fig. 3C**), suggesting a potential link between integrin signaling and immune checkpoint regulation. Similarly, ITGB3 expression was significantly correlated with PD-L1 (R = 0.532, p = 1.00e-03; **Fig. 3D**), reinforcing the idea that integrin αvβ3 may influence immune evasion. A strong positive correlation was also observed between ITGAV and ITGB3 (R = 0.615, p = 9.19e-05), indicating coordinated expression of these integrin subunits. EGFR expression was moderately correlated with both ITGAV (R = 0.462, p = 4.96e-03; **Fig. 3E**) and PD-L1 (R = 0.13, p = 0.39; non-significant), suggesting that while EGFR might not directly regulate PD-L1 transcriptionally, it could modulate immune escape through integrin-associated signaling pathways. EGFR showed no significant correlation with ITGB3 (R = 0.07, p = 0.65), indicating a more complex or context-dependent interaction between these molecules. These findings suggest that integrin αvβ3 and EGFR may synergize to promote cholangiocarcinoma progression by enhancing both migratory signaling and immune checkpoint expression. Targeting the crosstalk between these pathways could offer novel therapeutic strategies for cholangiocarcinoma.

### Correlation between Immune Cell Infiltration and Key Genes in Cholangiocarcinoma

**Figure 4:**
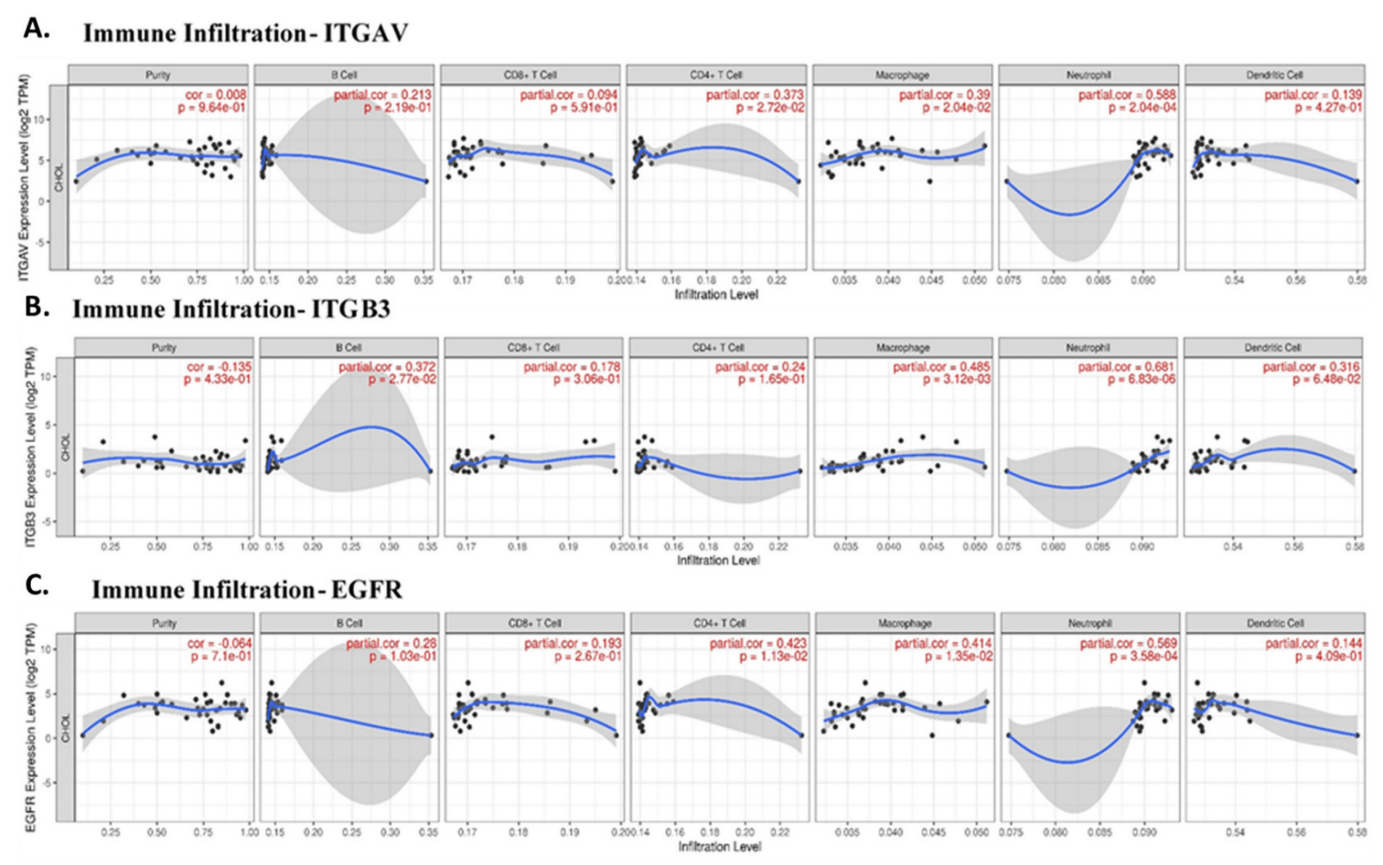
The correlation between the expression levels of ITGAV, ITGB3, and EGFR with the infiltration levels of various immune cell types in cholangiocarcinoma. A. Illustrates the correlation between ITGAV and immune cell infiltration, revealing a significant relationship with Neutrophils (partial correlation = 0.588, p = 2.04e-04), supporting the role of ITGAV in neutrophil recruitment in cholangiocarcinoma. CD4+ T cells also exhibit a notable correlation. B. Displays the correlation of ITGB3 with immune infiltration, with significant associations with Neutrophils (partial correlation = 0.661, p = 6.83e-06) and Macrophages (partial correlation = 0.485, p = 3.12e-03), indicating a potential role of ITGB3 in immune cell recruitment and tumor progression. C. Shows the relationship between EGFR expression and immune cell infiltration, with notable neutrophils (partial correlation = 0.569, p = 3.58e-04), suggesting that EGFR expression is associated with immune cell infiltration, particularly in the context of immune modulation. B Cells and CD8+ T cells show weaker correlations. These findings suggest that EGFR, ITGAV, and ITGB3 play significant roles in modulating the immune microenvironment, particularly in relation to immune cells like neutrophils, macrophages, and CD4+ T cells, which are essential in tumor immunity.

Given the role of integrins and EGFR in immune modulation, we next explored the correlation between these genes and immune cell infiltration in cholangiocarcinoma. Immune cells such as neutrophils, macrophages, and CD4+ T cells are integral components of the tumor microenvironment and can either promote or inhibit tumor progression, depending on their polarization. Our analysis revealed that ITGAV expression was significantly correlated with neutrophil infiltration (partial correlation = 0.588, p = 2.04e-04), supporting the idea that ITGAV might be involved in recruiting neutrophils to the tumor site **(Fig. 4A)**. This result consists with previous reports showing that integrins are involved in immune cell trafficking and tumor inflammation. ITGB3 was also associated with neutrophil and macrophage infiltration **(Fig. 4B)**, suggesting that integrin signaling pathways play a vital role in shaping the immune landscape in cholangiocarcinoma. A significant correlation between EGFR expression and the infiltration of CD4+ T cells (partial correlation = 0.423, p = 1.13e-02) and neutrophils (partial correlation = 0.569, p = 3.58e-04) in cholangiocarcinoma tissues **(Fig. 4C)**. This result suggests that integrin αvβ3 and EGFR may play a role in recruiting and activating immune cells within the tumor microenvironment, particularly, neutrophils and T cells, which are important for anti-tumor immunity. Furthermore, together, these results highlight the potential of integrin αvβ3 and EGFR as modulators of the immune response in cholangiocarcinoma. These findings are crucial for understanding the immune dynamics in cholangiocarcinoma and may help inform therapeutic strategies aimed at reprogramming the immune microenvironment.

### Single-Cell RNA Sequencing Identifies Cell Type-Specific Expression of Integrin αvβ3, EGFR, and PD-L1 in Cholangiocarcinoma

**Figure 5:**
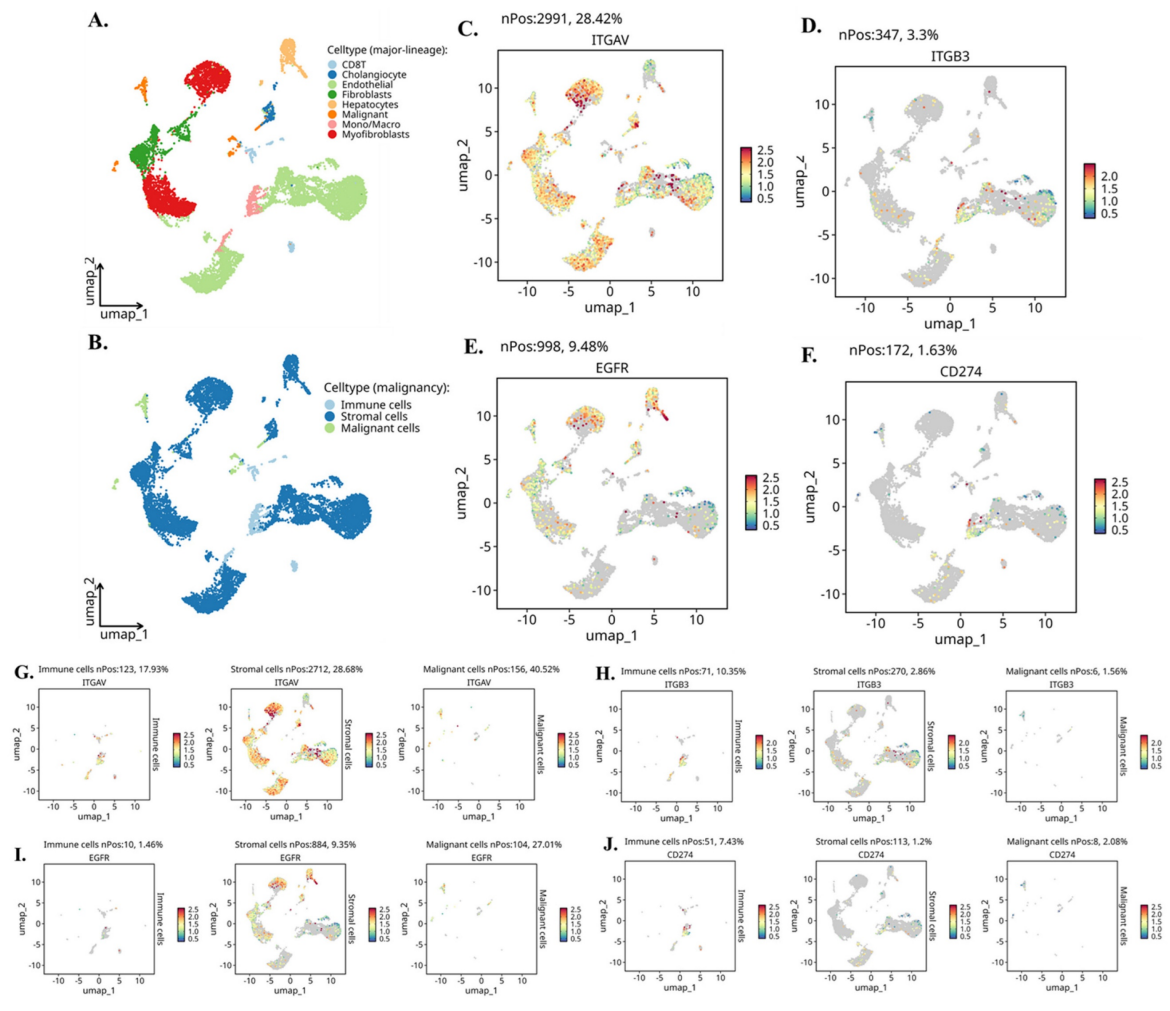
Single-cell expression landscape of ITGAV, ITGB3, EGFR, and CD274 in cholangiocarcinoma. (A) UMAP projection of the single-cell dataset showing major lineage identities, including cholangiocytes, endothelial cells, fibroblasts, hepatocytes, immune cells, malignant cells, monocytes/macrophages, and myofibroblasts. (B) UMAP classification of cells by malignancy status, separating immune, stromal, and malignant populations. (C-F) Gene-level UMAPs displaying the distribution and expression intensity of ITGAV (C), ITGB3 (D), EGFR (E), and CD274 (F) across all cell populations. Expressing cells are highlighted, and non-expressing cells are shown in grey; nPos values indicate the number and percentage of positive cells. (G-J) Compartment-specific expression of each gene across immune, stromal, and malignant cells. Panels show UMAPs re-projected within each compartment for ITGAV (G), ITGB3 (H), EGFR (I), and CD274 (J), illustrating differential enrichment across distinct cellular subsets. Expression intensity is represented using a continuous color scale.

To map the cellular sources and biological context of integrin αvβ3, EGFR, and PD-L1 expression in cholangiocarcinoma (CCA), we analyzed independent scRNA-seq dataset CHOL_GSE142784. UMAP clustering **(Fig. 5A-B)** clearly resolved malignant epithelial cells, non-malignant cholangiocytes, stromal fibroblasts and myofibroblasts, endothelial cells, hepatocytes, and multiple immune lineages including T cells and monocyte/macrophage populations. This provided a cellular atlas for identifying lineage-specific roles of integrin signaling and EGFR-driven pathways in the tumor microenvironment. Global gene-expression mapping revealed distinct biological functions associated with each molecule **(Fig. 5C-F)**. ITGAV (integrin αv) showed widespread distribution across malignant, stromal, and endothelial cells **(Fig. 5C)**, consistent with its known role as a broadly expressed adhesion receptor involved in ECM sensing, mechanotransduction, and pro-invasive remodeling. In contrast, ITGB3 (integrin β3) exhibited highly restricted expression **(Fig. 5D)**, appearing predominantly in fibroblasts, myofibroblasts, and a subset of macrophages. This pattern aligns with the biological requirement of β3 for generating the αvβ3 heterodimer specifically within activated stromal and inflammatory niches. EGFR expression was strongly enriched in malignant cholangiocytic cells **(Fig. 5E)**, reflecting its function as a proliferative and survival driver in CCA. CD274 (PD-L1) was primarily expressed by monocyte/macrophage populations **(Fig. 5F)**, supporting its role in shaping immunosuppressive circuits through myeloid-driven checkpoint regulation.

Compartment-specific reanalysis **(Fig. 5G-J)** further uncovered a functionally coordinated division of labor among malignant, stromal, and immune lineages. ITGAV remained most abundant in malignant epithelial cells **(Fig. 5G, left)**, indicating that tumor cells provide the αv subunit necessary for ligand recognition and downstream FAK/Src activation. Stromal fibroblasts and myofibroblasts generated the highest levels of ITGB3 **(Fig. 5H, middle)**, confirming that the β3 subunit originates from stromal sources and may heterodimerize with tumor-derived αv in a paracrine manner. EGFR expression was again dominant in malignant cells **(Fig. 5I, right)**, highlighting the tumor-intrinsic reliance on EGFR-mediated mitogenic signaling. CD274 showed strong enrichment in immune cells, especially macrophages **(Fig. 5J, left)**, indicating that the immunosuppressive PD-L1 signal is largely myeloid-driven, with minor contributions from malignant cells.

**Figure 6:**
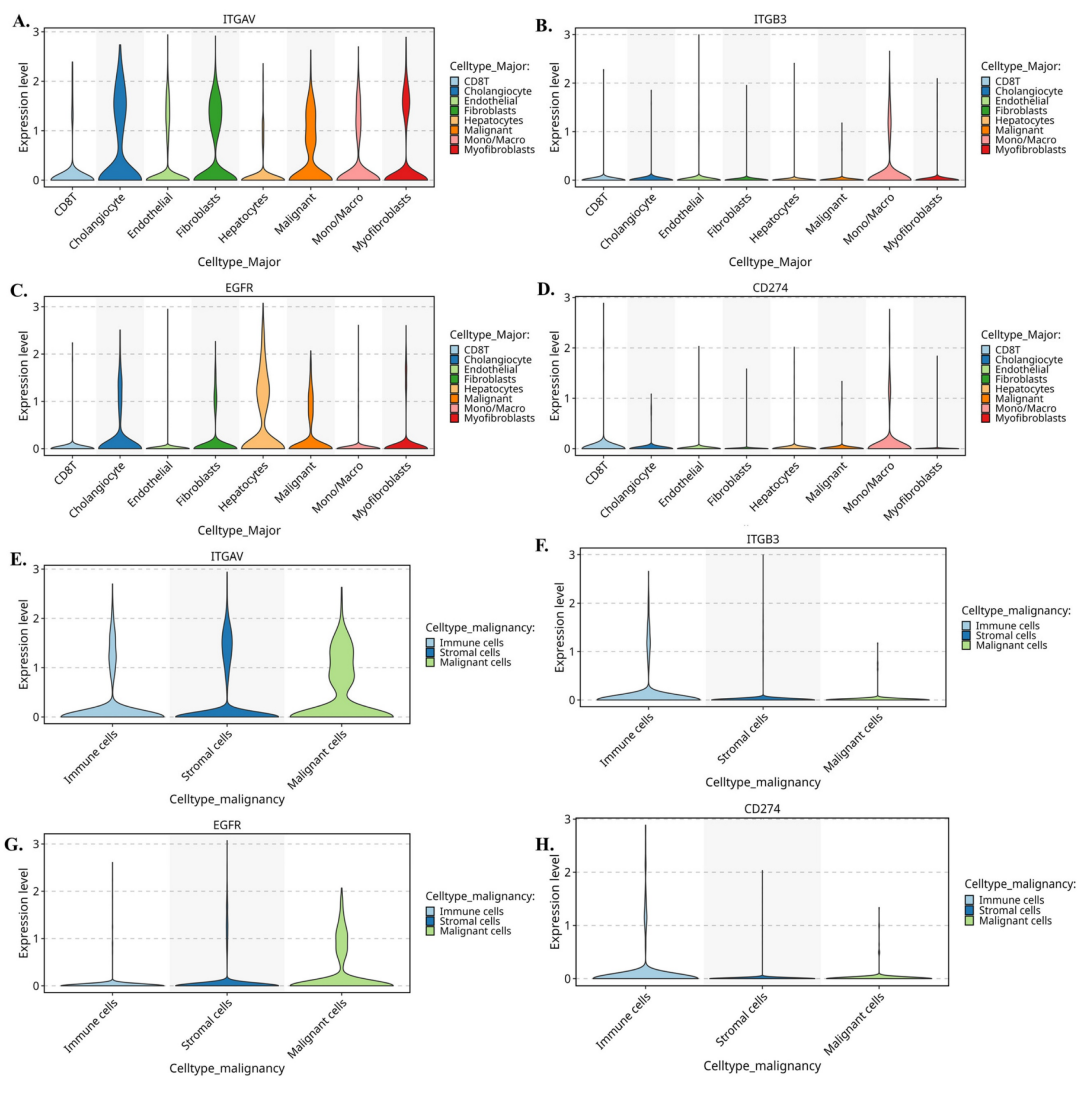
Single-cell expression patterns of ITGAV, ITGB3, EGFR, and CD274 across major cholangiocarcinoma cell populations. (A-D) Violin plots showing expression of ITGAV (A), ITGB3 (B), EGFR (C), and CD274 (D) across major cell types identified in the single-cell CCA atlas, including cholangiocytes, endothelial cells, fibroblasts, hepatocytes, macrophages/monocytes, myofibroblasts, NK/T cells, and B cells. (E-H) Violin plots comparing the expression of ITGAV (E), ITGB3 (F), EGFR (G), and CD274 (H) among malignant epithelial cells, stromal cells, and immune cells. Malignant epithelial cells show higher expression of ITGAV and EGFR, while ITGB3 is enriched in stromal and macrophage populations. CD274 is detected in both malignant and immune subsets.

Further we evaluate the violin plots **(Fig. 6A-D)** which quantify these patterns and further reinforce the compartmental specificity. In Figure 6A, ITGAV expression peaks in malignant epithelial cells, with moderate levels in stromal fibroblasts and endothelial cells. In Figure 6B, ITGB3 shows a striking stromal bias, being highly expressed in fibroblasts, myofibroblasts, and macrophages. Figure 6C demonstrates that EGFR is strongly enriched in malignant cells, while Figure 6D shows CD274 expression primarily in monocyte/macrophage populations. Moreover, when these cells are reorganized into malignant, stromal, and immune compartments, the distribution becomes even clearer. Figure 6E shows that ITGAV remains dominant in malignant cells, while Figure 6F confirms stromal localization of ITGB3. Figure 6G highlights malignant enrichment of EGFR, and Figure 6H demonstrates that CD274 is mainly immune-derived with modest expression in malignant cells. Together, these patterns reveal a functional division in which malignant cells supply αv and EGFR, while stromal fibroblasts and immune cells contribute β3 and PD-L1.

**Figure 7:**
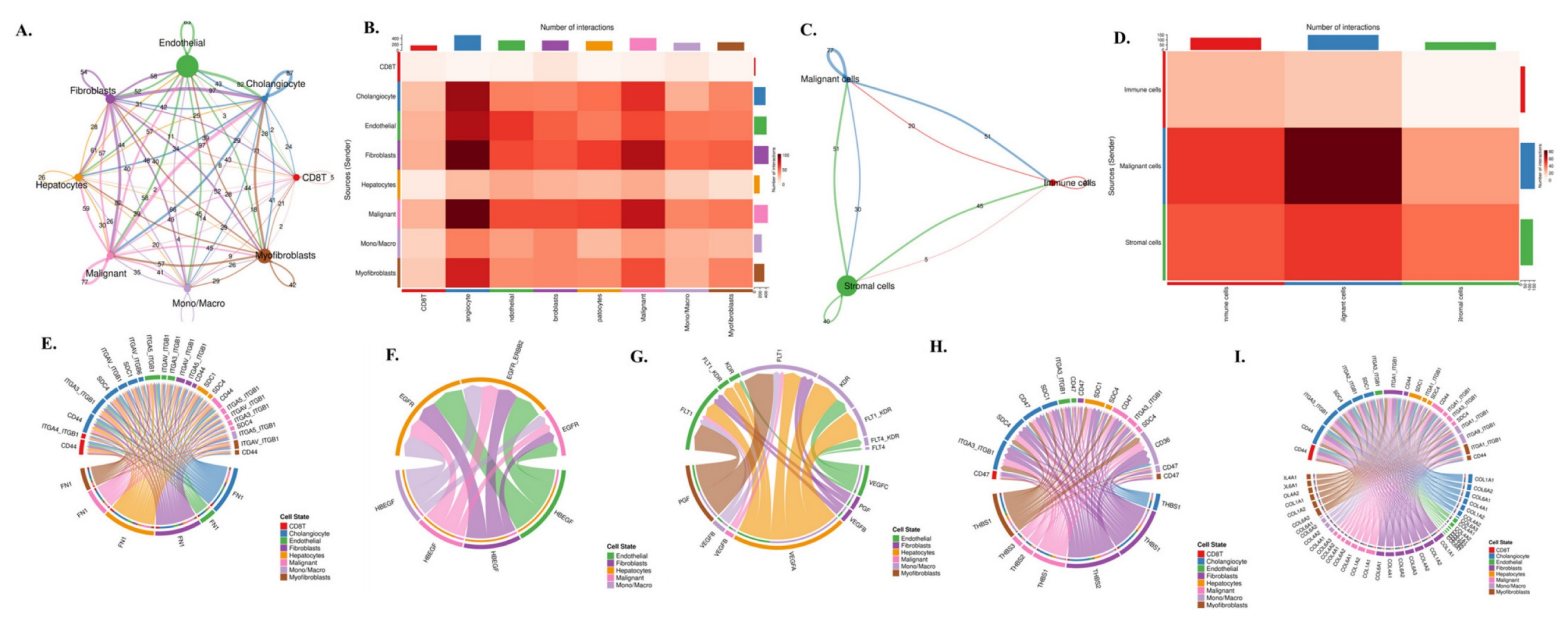
Cell-cell communication network and ligand-receptor signaling architecture in cholangiocarcinoma. (A) Global cell-cell communication network inferred from CellChat, showing the number and strength of outgoing and incoming interactions among cholangiocytes, endothelial cells, fibroblasts, hepatocytes, malignant epithelial cells, monocyte/macrophage lineages, myofibroblasts, and CD8 T cells. Node size represents overall communication strength, and edge thickness corresponds to interaction probability. (B) Heatmap summarizing the total number of ligand-receptor interactions between each pair of cell types, illustrating the dominant communication routes across the tumor microenvironment. (C) Simplified three-compartment interaction network showing signaling exchanges among malignant, stromal, and immune cells. Edge direction and thickness indicate the magnitude of outgoing and incoming signals for each compartment. (D) Heatmap of compartment-level interactions depicting the number of signals transmitted from stromal, malignant, and immune populations to one another. (E) Chord diagram illustrating ECM-integrin signaling interactions, including FN1-, COL1A1-, COL3A1-, and THBS1-mediated binding to integrin complexes such as ITGAV-ITGB3 and CD44 across multiple cell types. (F) Chord diagram of EGFR-related signaling pathways showing ligand-receptor pairs such as HBEGF-EGFR and EREG-EGFR among endothelial, fibroblast, malignant, and immune populations. (G) Chord diagram showing VEGF and FLT signaling interactions, including VEGFA-FLT1/FLT4 and related endothelial and stromal communication circuits. (H) Chord diagram mapping the SPP1-CD44 and SPP1-ITG ligand-integrin axes, highlighting macrophage-derived signaling to malignant and stromal compartments. (I) Chord diagram summarizing global ligand-receptor interactions among all cell types, integrating ECM, immune, angiogenic, and growth factor pathways within the cholangiocarcinoma microenvironment.

To investigate how these lineage-specific expression patterns translate into functional communication within the tumor microenvironment, we performed a comprehensive CellChat analysis **(Fig. 7A-I)**. The global interaction network **(Fig. 7A)** revealed dense bidirectional signaling among nearly all cell types, with endothelial cells, fibroblasts, and malignant epithelial cells functioning as major communication hubs. Quantification of ligand-receptor pairs **(Fig. 7B)** showed that malignant cells receive a disproportionately high number of incoming signals, particularly from fibroblasts, myofibroblasts, and monocyte/macrophage populations. When the data were simplified into malignant, stromal, and immune compartments **(Fig. 7C)**, stromal-to-malignant communication emerged as the dominant axis, followed by strong immune-to-malignant signaling. This was further supported by compartment-level interaction counts **(Fig. 7D)**, where malignant cells appeared as the primary signal recipients in the tri-compartment network. To dissect the molecular nature of these interactions, we mapped specific ligand-receptor families that regulate tumo stroma immune cross-talk. ECM-integrin signaling **(Fig. 7E)** showed strong fibroblast- and myofibroblast-derived FN1, COL1A1, COL3A1, and THBS1 engaging integrin complexes such as ITGAV-ITGB3 and CD44 on malignant cells. EGFR-driven pathways **(Fig. 7F)** demonstrated that ligands including HBEGF and EREG originate mainly from endothelial and fibroblast populations, delivering mitogenic cues directly to EGFR-high malignant cells. Angiogenic circuits involving VEGFA, FLT1, and FLT4 **(Fig. 7G)** were primarily exchanged between malignant, stromal, and endothelial compartments, consistent with vascular remodeling in cholangiocarcinoma. Macrophage-derived SPP1 formed a dominant immunomodulatory axis by binding to ITGAV/ITGB3 and CD44 receptors on malignant and stromal cells **(Fig. 7H)**, reinforcing tumor-supportive and immune-suppressive signaling. Finally, the integrated ligand-receptor map **(Fig. 7I)** combines ECM, growth factor, angiogenic, and immune pathways, illustrating the highly coordinated and multi-layered communication structure underpinning cholangiocarcinoma progression.

Taken together, these integrated single-cell and CellChat analyses reveal a highly structured and compartment-specific signaling architecture within the cholangiocarcinoma microenvironment. Malignant epithelial cells emerge as a central ITGAV-high and EGFR-high population, positioning them as the dominant recipients of proliferative, pro-survival, and ECM-driven mechanotransductive cues. In contrast, stromal fibroblasts and myofibroblasts form an ITGB3-rich niche that supplies the key β3 subunit required for assembling the functional αvβ3 integrin complex, while also producing abundant ECM ligands such as FN1, COL1A1, COL3A1, and THBS1 that activate integrin signaling on tumor cells. Meanwhile, macrophages serve as the primary source of PD-L1 and secrete SPP1, reinforcing immune suppression and potentiating integrin-dependent communication with malignant and stromal compartments. These coordinated exchanges create a directional signaling hierarchy in which stromal and immune cells continuously feed integrin-activating, EGFR-stimulating, and immune-modulating signals to malignant cells. Collectively, this framework highlights a cooperative tri-compartment ecosystem malignant, stromal, and immune that sustains tumor growth, invasion, angiogenesis, and immune evasion through tightly integrated αvβ3, EGFR, and PD-L1 signaling circuits.

### Protein-Level Validation of ITGAV, ITGB3, EGFR, and PD-L1 Expression in Cholangiocarcinoma Tissues

**Figure 8:**
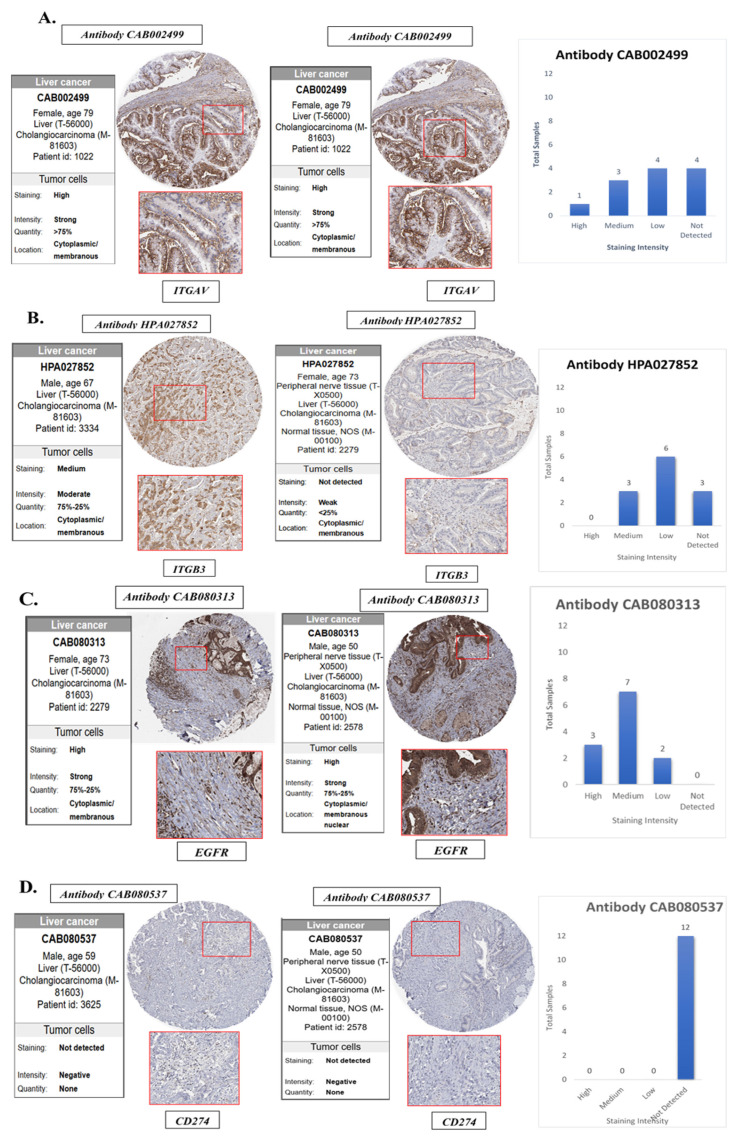
Validation of the protein expression levels of EGFR, integrin αvβ3 in normal bile duct tissue and cholangiocarcinoma tissues. (A) ITGAV, (B) ITGB3, (C) EGFR and (D) CD274(PD-L1) protein expression were analyzed in bile ducts cholangiocarcinoma tissues using HPA database. HPA, the human protein atlas.

To validate the transcriptomic and single-cell findings at the protein level, we utilized immunohistochemistry (IHC) data from The Human Protein Atlas (HPA) to assess the expression of ITGAV, ITGB3, EGFR, and PD-L1 in cholangiocarcinoma tissues and normal bile duct controls **(Fig. 8)**. This analysis aimed to confirm whether the elevated mRNA expression of these genes is reflected at the protein level in clinical tumor specimens, thereby strengthening their translational relevance. The IHC images revealed that ITGAV, ITGB3, and EGFR proteins were highly expressed in cholangiocarcinoma tissues compared to normal bile ducts. ITGAV showed strong membranous and cytoplasmic staining in tumor epithelial cells, consistent with its role in cell adhesion and integrin-mediated signaling. ITGB3 exhibited a similar localization pattern, reinforcing its involvement in forming the αvβ3 heterodimer and facilitating tumor invasion. EGFR was prominently expressed in the membrane and cytoplasm of malignant cholangiocytes, supporting its known function as a driver of proliferative and survival signaling in tumors. In contrast, PD-L1 (CD274) protein expression was undetectable in the cholangiocarcinoma tissues analyzed, despite its upregulation at the mRNA level in bulk and single-cell RNA sequencing data. This discrepancy suggests that PD-L1 may be regulated post-transcriptionally or its expression may be context-dependent, induced only under specific microenvironmental stimuli such as cytokine exposure or immune pressure. Alternatively, it may reflect tumor heterogeneity, where only subsets of cholangiocarcinoma cases exhibit detectable PD-L1 protein expression.

Collectively, these protein-level findings confirm the elevated expression of ITGAV, ITGB3, and EGFR in human cholangiocarcinoma and highlight them as robust biomarkers and potential therapeutic targets. Despite transcriptomic evidence, the absence of detectable PD-L1 protein underscores the need for integrated multi-level analyses when evaluating immune checkpoint markers in cancer **(Fig. 8)**.

### Pathway Enrichment Analysis of Integrin αvβ3, EGFR, and PD-L1 in Cholangiocarcinoma

**Figure 9:**
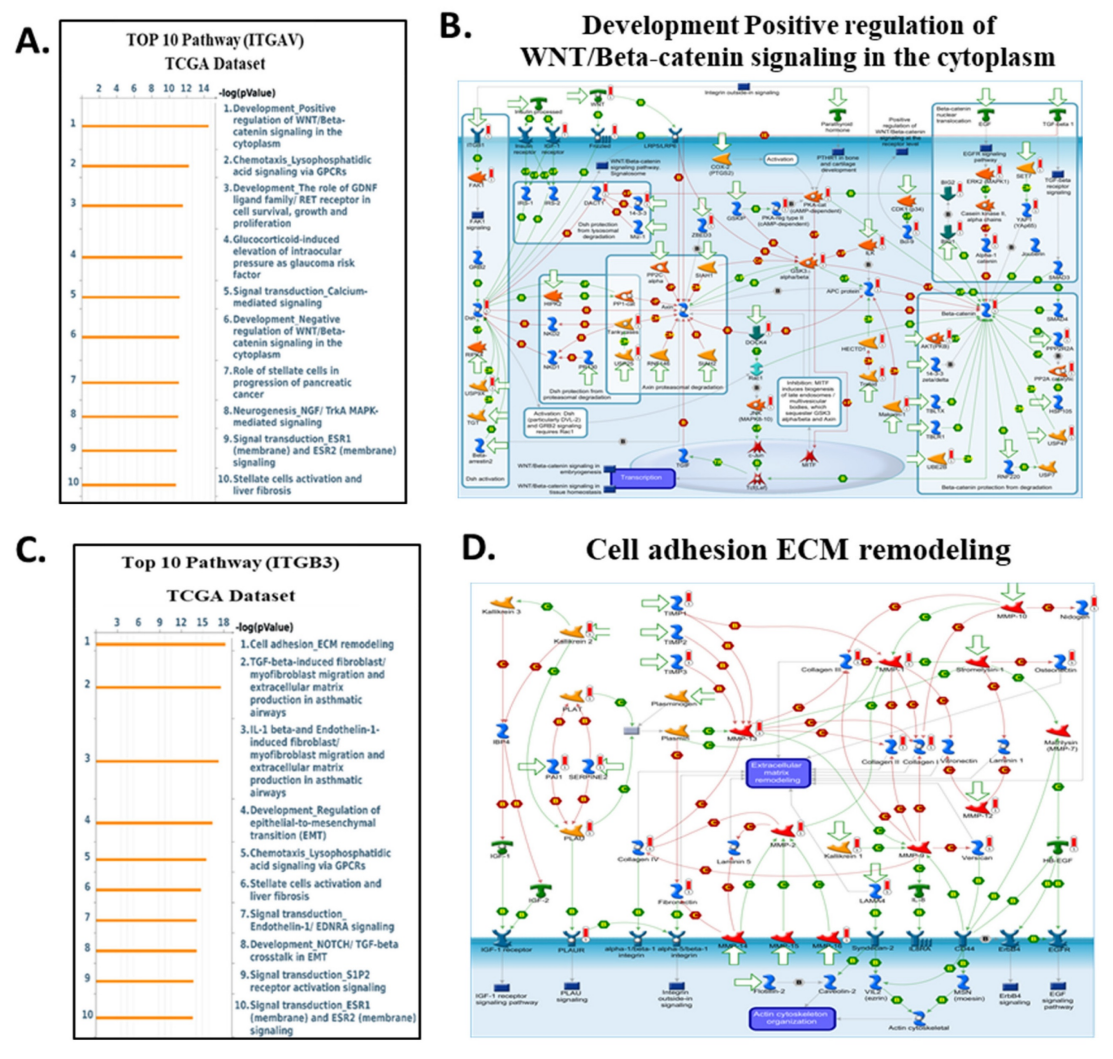
Top enriched pathways and signaling diagrams associated with ITGAV and ITGB3 in the TCGA dataset. (A) Top 10 enriched pathways correlated with ITGAV expression in the TCGA dataset, ranked by -log₁₀(p-value). Prominent pathways include WNT/β-catenin signaling, chemotaxis via GPCRs, and MAPK-mediated signaling. (B) Metacore diagram depicting the WNT/β-catenin signaling cascade, highlighting molecules correlated with ITGAV expression. Molecules upregulated are marked in red, downregulated in green, with z-score predictions shown in orange (activation) or blue (inhibition). (C) Top 10 enriched pathways associated with ITGB3 expression in the TCGA dataset. Key pathways include cell adhesion and extracellular matrix (ECM) remodeling, TGF-β signaling, and EMT regulation. (D) Metacore diagram of ECM remodeling and integrin signaling associated with ITGB3 expression. Color coding, as in panel B, illustrates the predicted regulatory effect.

**Figure 10:**
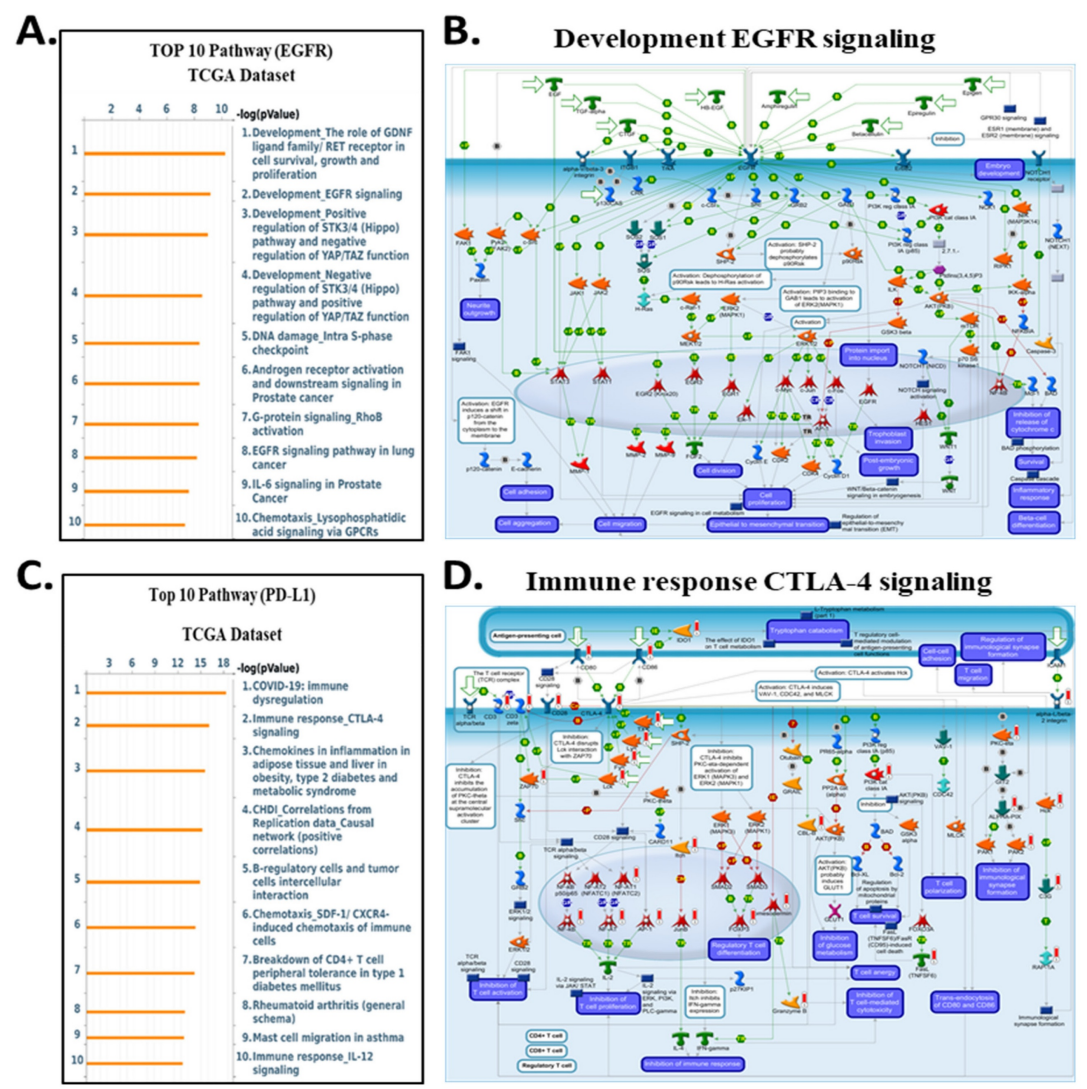
Top enriched pathways and signaling diagrams associated with PD-L1 and EGFR expression in the TCGA dataset. (A) Top 10 enriched pathways associated with EGFR expression in the TCGA dataset. Key pathways include RET/FGFR signaling, Hippo-YAP/TAZ pathway regulation, DNA damage response, and EGFR signaling in cancer progression. (B) Metacore diagram showing development and oncogenic signaling networks associated with EGFR expression. Major pathways include EGFR and GPCR signaling, cell proliferation, and migration pathways. As described in panel B, color coding highlights molecular expression and predicted regulatory outcomes. (C) Top 10 enriched pathways correlated with PD-L1 expression, ranked by -log₁₀(p-value). Prominent pathways include immune dysregulation in COVID-19, CTLA-4 signaling, chemokine signaling in inflammation, and T-cell tolerance and migration, reflecting the immune regulatory role of PD-L1. (D) Metacore signaling diagram illustrating immune-related pathways associated with PD-L1 expression. Key elements include T-cell activation, antigen presentation, and cytokine signaling. Nodes are colored to reflect gene expression: red for upregulated, green for downregulated. Predicted pathway activity is shown in orange (activation) and blue (inhibition).

To clarify the downstream biological programs governed by integrin αvβ3, EGFR, and CD274 (PD-L1) in cholangiocarcinoma, we analyse the MetaCore enrichment results into coherent mechanistic modules rather than listing individual pathways. This allowed us to identify the dominant oncogenic, stromal, and immune circuits that converge downstream of the ITGAV, ITGB3, EGFR, and CD274 expression in the TCGA-CHOL dataset **(Fig. 9-10)**. First, pathway ITGAV, the most prominent enrichments involved ECM remodeling, cytoskeletal reorganization, mechanotransduction, and WNT/β-catenin signaling **(Fig. 9A-B)**. These findings are consistent with the established role of integrin αvβ3 in translating matrix stiffness and stromal cues into intracellular signaling outputs that promote cell adhesion, migration, and EMT-related transcriptional programs. Mechanistically, genes associated with ITGAV expression were embedded in pathways regulating fibroblast activation, LOX/LOXL1-mediated matrix crosslinking, and GPCR-mediated chemotaxis, reinforcing the central role of αvβ3 in coordinating extracellular matrix-derived signals during tumor progression. Similarly, ITGB3 expression correlated strongly with pathways associated with epithelial-mesenchymal transition, TGF-β-NOTCH crosstalk, and cell adhesion dependent signaling **(Fig. 9C-D)**. MetaCore network maps highlighted increased integrin-FAK interactions, ECM degradation, and cytoskeletal rearrangement, all of which support the notion that ITGB3 enhances cellular plasticity and invasive remodeling within the cholangiocarcinoma microenvironment. These results position integrin αvβ3 as a key upstream regulator of structural and transcriptional reprogramming in CCA.

In contrast, genes correlated with EGFR expression enriched for classical oncogenic pathways, including RET/FGFR-MAPK signaling, Hippo-YAP/TAZ regulation, growth factor-mediated migration, and DNA damage-associated signaling **(Fig. 10A-B)**. These enrichments are consistent with EGFR's known involvement in proliferative and survival pathways, and they align with prior reports demonstrating that integrin-mediated EGFR cross-activation amplifies ERK and PI3K/AKT output to support tumor progression. For CD274 (PD-L1), MetaCore analysis highlighted immune-related pathways such as CTLA-4 signaling, cytokine-driven tolerance, chemokine responses, and T-cell exhaustion signatures **(Fig. 10C-D)**. These findings reinforce the functional linkage between inflammatory transcriptional programsparticularly those involving STAT3, NF-κB, and β-catenin and PD-L1 upregulation, supporting a tumor cell-intrinsic mechanism of immune suppression in cholangiocarcinoma. Supplementary Figures S1-S8 and Supplementary Tables S2-S5 further expand these observations, providing complete lists of enriched pathways, gene networks, and statistical associations. Together, these analyses reveal that ITGAV, ITGB3, EGFR, and CD274 converge on interconnected processes involving ECM remodeling, mechanotransduction, proliferative ERK/AKT signaling, transcriptional plasticity, and immune escape. This integrated MetaCore framework aligns closely with the central signaling architecture of our study and strengthens the therapeutic rationale for targeting the ITGAV/ITGB3-EGFR-PD-L1 axis in cholangiocarcinoma.

Collectively, these pathway findings position integrin αvβ3 as a master regulator of the molecular circuitry that shapes cholangiocarcinoma aggressiveness. Rather than functioning as a passive adhesion receptor, αvβ3 integrates cues from the extracellular matrix with intracellular growth factor signaling to coordinate ERK- and AKT-dependent proliferation, STAT3-driven inflammatory transcription, β-catenin-mediated plasticity, and PD-L1-associated immune evasion. MetaCore-derived networks consistently converged on these signaling hubs, and the corresponding pathway activation patterns were strongly reflected in our transcriptomic and cellular phenotypes across ITGAV-, ITGB3-, EGFR-, and CD274-high tumors. Importantly, the alignment between computational enrichment and functional data highlights a highly interconnected signaling axis in which αvβ3 amplifies EGFR activity, stabilizes β-catenin, reinforces EMT programs, and enhances PD-L1 expression to support tumor progression and immune suppression. These integrated observations underscore the therapeutic relevance of targeting αvβ3 by binding to the extracellular domain of the integrin, DL-N2 derivatives have the potential to dampen multiple oncogenic circuits simultaneously, offering a rational strategy to disrupt the ITGAV/ITGB3-EGFR-STAT3/β-catenin-PD-L1 axis and overcome multifaceted pathogenic mechanisms in cholangiocarcinoma.

### Thyroxine and EGF Induce Different Signaling Pathways to Stimulate Proliferation in KRAS Wild-Type SSP-25 Cells and KRAS Mutant HuCCT1 Cells

**Figure 11:**
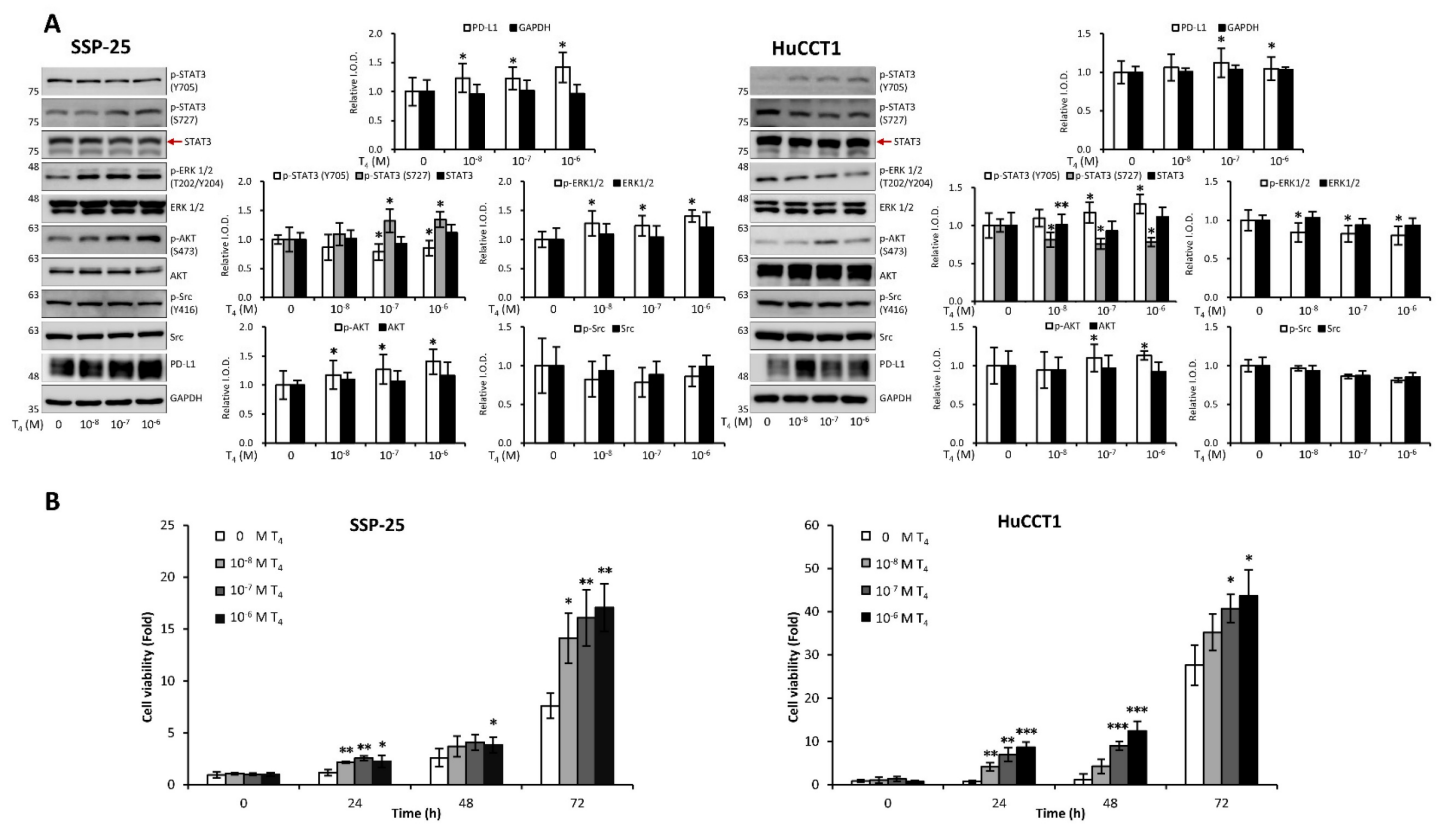
Thyroxine modulates signal transduction pathways in cholangiocarcinoma cells. (A). T_4_ modulates signal transduction pathways in cholangiocarcinoma cells. Serum-starved SSP-25 (left) and HuCCT1 cells (right) were left unstimulated or were stimulated with various concentrations of T_4_ (10^-8^, 10^-7^, and 10^-6^ M) for 24 h. Cells that did not receive stimulation with T_4_ were treated with KOH-PG buffer instead. After stimulation, the cells were lysed, and cell lysates were subjected to Western blotting to detect p-ERK1/2, ERK1/2, p-AKT, AKT, p-STAT3 (Ser727), p-STAT3 (Tyr705), STAT3, p-Src, Src, and PD-L1; GAPDH was used as a loading control for protein normalization. Quantitative results are expressed as relative integrated optical densities (IODs) by defining the amounts of the indicated detected proteins in unstimulated cells as 1. Data are presented as the mean ± SEM of three independent experiments. * p < 0.05 compared to unstimulated cells. (B). Thyroid hormone affects the growth of cholangiocarcinoma cells. Serum-starved SSP-25 (left) and HuCCT1 cells (right) were left unstimulated or stimulated with various concentrations of T_4_ (10^-8^, 10^-7^, and 10^-6^ M) for the indicated times. Cells that did not receive stimulation with T_4_ were treated with KOH-PG buffer instead. After stimulation, the cells were subjected to the alamar blue cell viability assay. The quantitative results were expressed as fold changes by defining the viability of the unstimulated group of each cell line at 0 h as 1. Data are represented as the mean ± SD of triplicate cultures in three independent experiments. * p < 0.05 and ** p < 0.01 compared to T_4_-unstimulated cells at the same time point.

Clinical studies highlight the role of integrin αvβ3 in cholangiocarcinoma, so we further examined thyroxine (T_4_) signaling in CCA cell models representing different anatomical origins. SSP-25 and HuCCT1 cells, both derived from intrahepatic CCA, and TFK-1, originating from perihilar CCA, provided a relevant platform to evaluate subtype-related signaling behavior. In KRAS wild-type SSP-25 cells, T_4_ activated ERK1/2 and AKT beginning at 10⁻⁸ M and induced STAT3 phosphorylation at 10⁻⁷ M without affecting Src **(Fig. 11A, left)**. In KRAS-mutant HuCCT1 cells, T_4_ showed an opposite pattern: ERK1/2 and STAT3 (S727) were suppressed at 10⁻⁸ M, whereas STAT3 (Y705) and AKT were activated from 10⁻⁷ M onward **(Fig. 11A, right)**. These results indicate that T_4_ engages integrin αvβ3 to regulate ERK, STAT3, and AKT signaling in a KRAS-dependent manner, and that these responses may differ across intrahepatic and extrahepatic CCA models. Studies suggest that thyroxine promotes cancer cell proliferation across various cancer types. We sought to determine whether thyroxine specifically stimulates cell growth in different KRAS status cholangiocarcinoma cells. KRAS wild-type SSP-25 and KRAS mutant HuCCT1 cells were stimulated with various concentrations of thyroxine for 24, 48, and 72 h to assess its effects on cell proliferation. T_4_ induced cell growth in SSP-25 cells starting at 10⁻⁸ M during the 24 to 72 h treatment period, whereas HuCCT1 cell growth was significantly stimulated at 10⁻⁷ and 10⁻⁶ M during the same period** (Fig. 11B, right)**.

**Figure 12:**
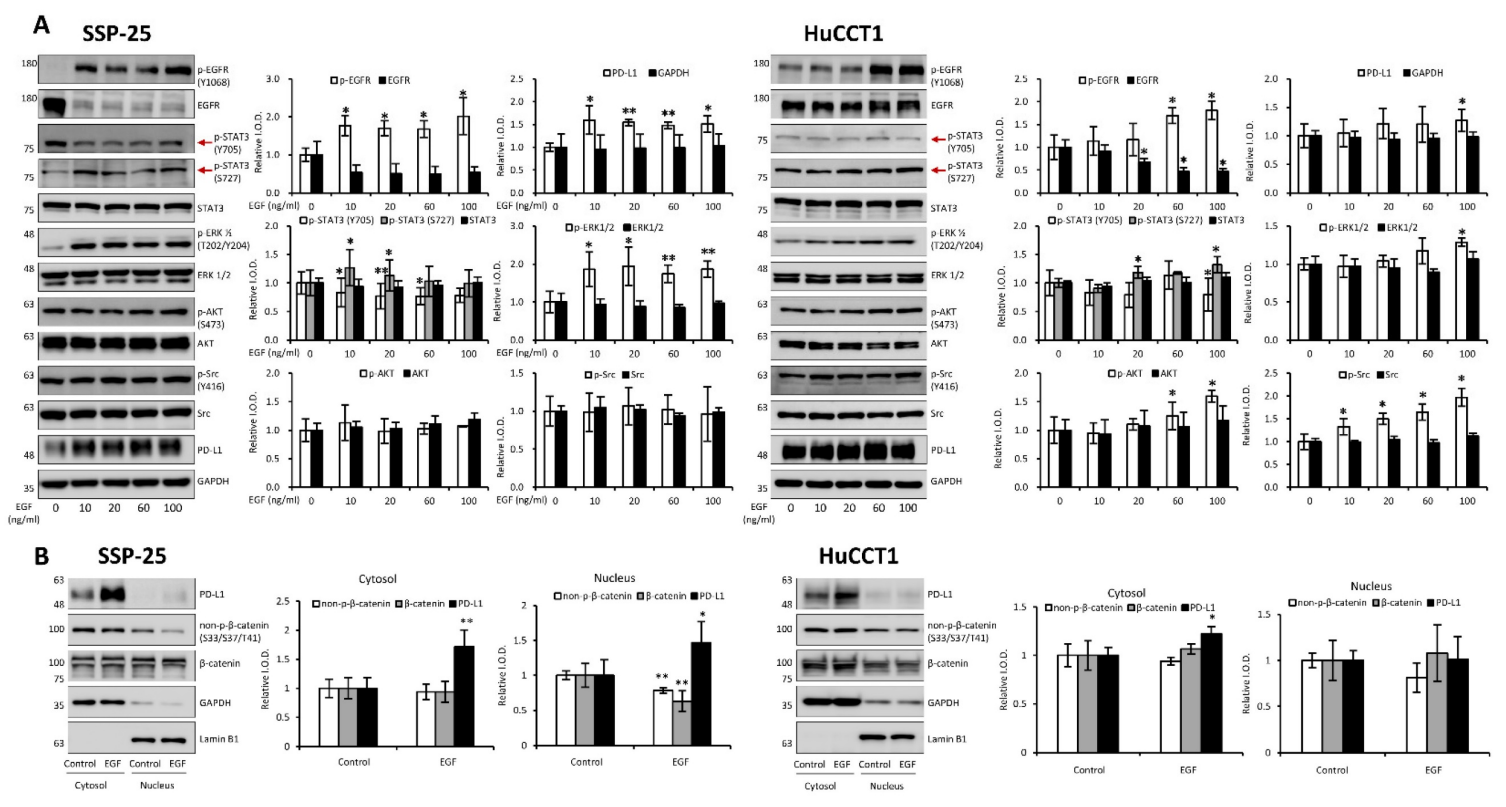
EGF modulates signal transduction pathways in cholangiocarcinoma cells. (A). EGF modulates signal transduction pathways. Serum-starved SSP-25 (left) and HuCCT1 cells (right) were left unstimulated or were stimulated with various concentrations of EGF (10, 20, 60, and 100 ng/ml) for 24 h. Cells that did not receive stimulation with EGF were instead treated with PBS. After stimulation, the cells were lysed, and cell lysates were subjected to Western blotting to detect p-EGFR, EGFR, p-ERK1/2, ERK1/2, p-AKT, AKT, p-STAT3 (Ser727), p-STAT3 (Tyr705), STAT3, p-Src, Src, and PD-L1; GAPDH was used as a loading control for protein normalization. Quantitative results are expressed as relative integrated optical densities (IODs) by defining the amounts of the indicated detected proteins in unstimulated cells as 1. Data are presented as the mean ± SEM of three independent experiments. * p < 0.05 and ** p < 0.01 compared to unstimulated cells. (B). EGF stimulation does not affect the translocation of PD-L1 from the cytosol to the nucleus in cholangiocarcinoma cells. Serum-starved SSP-25 (left) or HuCCT1 (right) cells were left unstimulated or stimulated with 20 or 100 ng/ml EGF, respectively, for 24 h. Cells that did not receive stimulation with EGF were instead treated with PBS. After stimulation, the cells were isolated into nuclear and cytosolic fractions, and then the cell lysates were subjected to Western blotting to detect PD-L1, non-p-β-catenin (Ser33/Ser37/Tyr41), and β-catenin; Lamin B1 and GAPDH were used as loading controls for nuclear and cytosolic fractions, respectively.

EGF has been shown to promote cholangiocarcinoma growth 35. However, KRAS wild-type SSP-25 cells showed greater sensitivity to EGF treatment 35. To further elucidate the molecular mechanisms, we investigated the signal transduction pathways activated by EGF in KRAS wild-type SSP-25 and KRAS mutant HuCCT1 cells. EGF treatment induced the activation of ERK1/2, AKT, and STAT3 pathways in both cancer cell lines, suggesting that EGF activated common signaling pathways to stimulate cancer cell proliferation **(Fig. 12A)**. EGFR and T_4_ share common signaling pathways, indicating the availability of signaling crosstalk in stimulating tumor growth. These findings highlighted the complexity of cholangiocarcinoma signaling and suggested that KRAS mutation influences the cellular signaling pathways activated by thyroxine (T_4_) and EGF, further complicating the tumor's response to these molecules.

It has been reported that thyroxine-induced nuclear translocated PD-L1 plays a vital role in cancer cell proliferation 36. Further evidence indicates that nuclear PD-L1 promotes angiogenesis in malignancies 37. To investigate whether EGF induces nuclear PD-L1 translocation, SSP-25 **(Fig. 12B, left)** and HuCCT1 **(Fig. 12B, right)** were stimulated with EGF for 24 h. Under EGF stimulation in these two cell lines, the upregulated PD-L1 was primarily found in the cytosol of both cell lines. However, a slight increase in nuclear PD-L1 was observed only in KRAS wild-type SSP-25 cells. In contrast, KRAS mutant HuCCT1 cells did not exhibit nuclear PD-L1 accumulation **(Fig. 12B)**. EGFR activation leads to β-catenin-mediated PD-L1 expression, promoting immune evasion in glioblastoma 38. In EGF-stimulated SSP-25 cells **(Fig. 12B, left)** but not in HuCCT1 cells **(Fig. 12B, right)**, a decrease in total levels of non-p-β-catenin and total β-catenin was observed in the nuclear fraction, suggesting p-β-catenin increased. Thus, EGF increased cytosolic PD-L1 expression, likely through β-catenin activation 39 in cholangiocarcinoma.

### Targeting Integrin αvβ3 Inhibits Cholangiocarcinoma Growth *In Vitro* and *In Vivo*

**Figure 13:**
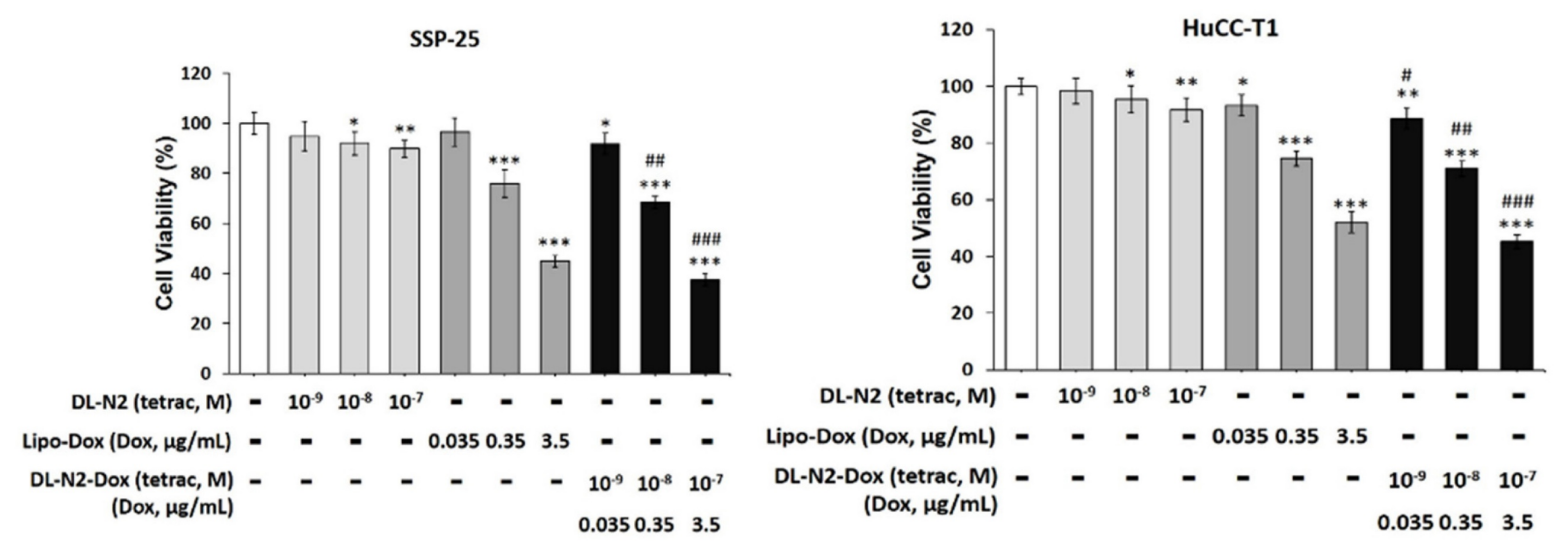
DL-N2 and DL-N2-Dox induced cytotoxicity in cholangiocarcinoma cells. A. SSP-25 cells and B. HuCCT1 cells were treated with DL-N2 (10^-9^ to 10^-7^ M), Lipo-Dox (0.035 µg/ml to 3.5 µg/ml) or DL-N2-Dox for 3 days. Cells were harvested, and cytotoxicity assays were conducted. Cells were harvested, and cytotoxicity assays were conducted. Data are presented as the mean ± SD. *p < 0.05, **p < 0.01, ***p < 0.001 as compared with control. #p < 0.05, ##p < 0.01, ###p < 0.001 as compared with corresponded Lipo-Dox.

We investigated targeting integrin αvβ3 to inhibit cholangiocarcinoma growth by a liposome-linked tetraiodothyroacetic acid (DL-N2). Two cholangiocarcinoma cell lines, SSP-25 and HuCCT1 cells, were treated with DL-N2, Lipo-Dox, or DL-N2 payload with Dox (DL-N2-Dox) to assess their cytotoxic effects **(Fig. 13)**. SSP-25 and HuCCT1 cells were exposed to a single dose of varying concentrations of DL-N2, Lipo-Dox, or DL-N2-Dox and incubated for three days. Following incubation, the cells were harvested for cytotoxicity assays to assess the effects of the treatments. DN-N2, Lipo-Dox, and DL-N2-Dox reduced cell viability in both cholangiocarcinoma cell lines. Notably, the DL-N2-Dox exhibited a more substantial antiproliferative effect on cancer cells **(Fig. 13)**. This treatment also significantly suppressed cholangiocarcinoma proliferation, particularly in KRAS wild-type SSP-25 cells.

**Figure 14:**
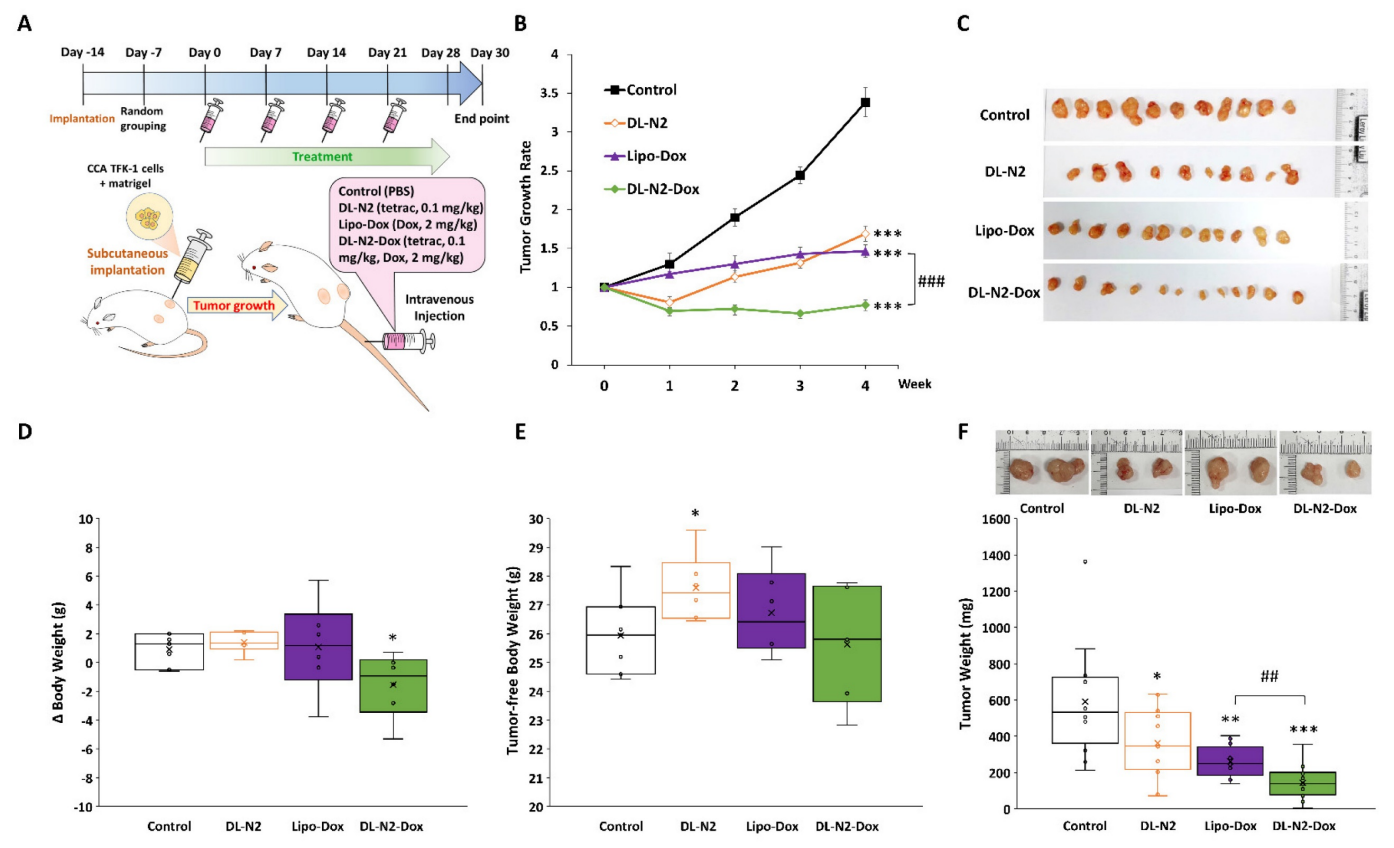
DL-N2 and DL-N2-Dox inhibited the tumor growth in cholangiocarcinoma TFK-1 xenograft study. (A) Schematic protocol of cholangiocarcinoma xenograft model. After TFK-1 cancer cell inoculation in the NOD SCID mice, the mice were treated with solvent (PBS), DL-N2 (tetrac, 0.1 mg/kg), Lipo-Dox (Dox, 2 mg/kg) or DL-N2-Dox (tetrac, 0.1 mg/kg, Dox, 2 mg/kg) one dose per week for 4 weeks. After 4 weeks of treatment, mice were sacrificed, and tumors were harvested and weighed. (B) The growth rate of CCA tumors during the experiment. The growth curve of tumor volume is normalized to the volume at the beginning of the treatment. Data are shown as Mean ± SEM. *p<0.05, **p<0.01, ***p<0.001 as compared with control. ###p<0.001, as compared with Lipo-Dox. (C) Images of xenografted tumors collected from each group. (D) Changes in body weight over the 4-week treatment. (E) Tumor-free body weight of xenograft mice, calculated by subtracting tumor weight from the total body weight of each mouse on the day of sacrifice. (F) The weight of CCA tumors collected from the sacrificed mice. *p<0.05, **p<0.01, ***p<0.001, as compared with control. ##p<0.01, as compared with Lipo-Dox.

Next, we conducted the anti-tumor growth effect of DL-N2 and its derivatives in cholangiocarcinoma xenografted mice. Since cholangiocarcinoma SSP-25 and HuCCT1 cells exhibit slow growth (doubling times of 64 and 55 hours, respectively) and have difficulty forming tumors in xenografted animals, 1 × 10^7^ cholangiocarcinoma TFK-1 cells were inoculated in mice as described in the Materials and Methods. After the TFK-1 cells had grown for 14 days and formed tumors, mice were intravenously injected with DL-N2 (tetrac 0.1 mg/kg), Lipo-Dox (Dox 2mg/kg), or DL-N2-Dox (tetrac 0.1 mg/kg, Dox 2mg/kg) once per week for four weeks. The schematic protocol of the cholangiocarcinoma xenograft model is presented in **Fig. 14A**. DL-N2, Lipo-Dox, and DL-N2-Dox all significantly reduced tumor growth rates compared with the control group. DL-N2 and DL-N2-Dox significantly reduced tumor growth after one week of treatment, whereas Lipo-Dox required two weeks to reduce tumor growth significantly. Moreover, DL-N2-Dox exhibited a markedly more potent effect than Lipo-Dox **(Fig. 14B)**. After 4 weeks of treatment, mice were sacrificed, and tumors were harvested and weighed. The xenograft tumors were collected from each group and are presented in **Fig. 14C**. It was observed that the tumors treated with liposomal drugs, DL-N2, Lipo-Dox, or DL-N2-Dox, were significantly smaller than those in the control group. Among all treatment groups, the tumors in the DL-N2-Dox group were remarkably small, exhibiting the most significant size reduction. After treatment, the weight changes of mice in the DL-N2 and Lipo-Dox groups were not significantly different from those in the control group. However, the weight changes of mice in the DL-N2-Dox group differed from those in the control group **(Fig. 14D)**. Additionally, the tumor-free body weight of mice in the DL-N2 group was significantly higher than that of the control group. In contrast, no significant difference was observed in the tumor-free body weight of mice in the Control, Lipo-Dox, and DL-N2-Dox groups **(Fig. 14E)**. The tumor weights of DL-N2, Lipo-Dox, and DL-N2-Dox were significantly lower than those in the control group, with the tumor weight of DL-N2-Dox being considerably lighter than that of Lipo-Dox **(Fig. 14F)**. Similarly, based on the overall results, the changes in body weight observed in the control group were attributed to tumor growth. In contrast, the significant decrease in body weight change in the DL-N2-Dox group was due to tumor reduction. Furthermore, DL-N2 effectively inhibited tumor growth and prevented weight loss. In contrast to the control group, where tumor burden contributed to weight changes, the DL-N2 group exhibited an increase in tumor-free body weight, further demonstrating the biological safety of DL-N2.

## Discussion

Cholangiocarcinoma represents a biologically heterogeneous group of biliary tract cancers composed of intrahepatic (iCCA), perihilar (pCCA), and distal (dCCA) subtypes 6. These entities differ markedly in stromal architecture, immune infiltration, and dominant oncogenic pathways, creating major challenges for therapeutic development. The public datasets used in this study including TCGA-CHOL and available single-cell atlases are predominantly derived from intrahepatic CCA, as large-scale transcriptomic resources for pCCA and dCCA remain limited 40. This imbalance has hindered a comprehensive understanding of how integrin αvβ3, EGFR, and PD-L1 signaling operate across the full spectrum of CCA subtypes. Nevertheless, the biological divergence among these subtypes strongly suggests that integrin-centered signaling hubs may not be uniformly conserved. iCCA, for example, is characterized by a dense desmoplastic reaction and LOXL1-enriched matrix that enhances mechanotransduction through αvβ3 and amplifies downstream YAP/TAZ, STAT3, and β-catenin activation 41, 42. In contrast, pCCA) and dCCA often exhibit distinct ductal architecture, bile flow dynamics, and inflammatory microenvironments that may influence FN1/COL1A1 integrin-EGFR crosstalk or alter dependence on αvβ3-mediated adhesion and invasion 43. Interestingly, despite these differences, ITGAV, ITGB3, and EGFR remain tightly co-expressed across multiple datasets, suggesting that αvβ3-driven signaling may represent a shared pathogenic axis across CCA subtypes, although with subtype-specific intensities rather than entirely distinct mechanisms. Future subtype-stratified analyses using balanced sampling, spatial transcriptomics, and stromal cell-resolved profiling will be essential to determine whether αvβ3 represents a universal therapeutic node across cholangiocarcinoma or a selectively targetable vulnerability enriched within iCCA.

Our analysis indicates that integrin αvβ3 regulates PD-L1 expression through a primarily tumor cell-intrinsic mechanism, rather than via macrophage-dependent mechanotransduction. All in vitro experiments were conducted in purified cholangiocarcinoma cell lines devoid of immune or stromal components, allowing us to isolate integrin-EGFR intracellular crosstalk. Under these conditions, integrin αvβ3 activation enhanced the FAK/SRC, ERK1/2, AKT, β-catenin, and STAT3 pathways canonical transcriptional regulators of PD-L1. The rapid induction of PD-L1 following T_4_ or EGF stimulation, and its equally rapid suppression by tetrac or DL-N2, further supports a direct signaling relationship. Although macrophage mechanotransduction and matrix stiffness may modulate PD-L1 in vivo, these influences were absent from our experimental system. Thus, our findings support a model in which integrin-dependent signaling upregulates PD-L1 directly within malignant cholangiocarcinoma cells. Future work incorporating tumor-immune co-culture or spatial profiling could help delineate additional microenvironmental contributions that may shape the therapeutic potential of integrin-ICI combinations.

Previous work has studied integrins or EGFR in isolation, but a unified mechanistic framework linking integrin αvβ3, EGFR, STAT3/β-catenin activation, and PD-L1 induction in cholangiocarcinoma has not been established 44, 45. Likewise, the interplay between thyroid hormone signaling, EGFR activity, and KRAS mutational status remains poorly defined. Few studies have explored how integrin αvβ3 contributes to immune-cell recruitment, how stromal ligands shape integrin activation, or how integrin inhibition could cooperate with EGFR-targeted therapy in KRAS-mutant CCA 46, 47. These knowledge gaps limit the translational rationale for developing integrin-targeted therapeutics.

Also, these translational challenges surrounding integrin-directed therapies reflect lessons learned from earlier clinical programs. Several αvβ3 antagonists, including cilengitide, failed to demonstrate durable clinical benefit owing to suboptimal pharmacokinetics, poor tumor penetration, and lack of integrin-selective patient stratification 48. Many early trials relied on pan-integrin inhibition or high-affinity ligands that paradoxically reduced vascular delivery and tissue exposure 49. Importantly, these therapeutic attempts occurred in tumor types with limited stromal stiffness or weak integrin dependence. More recent studies emphasize the need for affinity tuning, specificity for pathological rather than physiological integrin states, and rational combination strategies with EGFR inhibitors or immune checkpoint blockade. In contrast to prior settings, cholangiocarcinoma presents a markedly integrin-dependent stromal landscape, characterized by LOXL1-driven matrix crosslinking, strong αvβ3-EGFR-STAT3/β-catenin co-activation, and robust PD-L1 induction 43. These features provide a mechanistically grounded rationale for revisiting αvβ3-targeted therapy. DL-N2 derivatives, which selectively bind the extracellular domain of integrin αvβ3 and disrupt downstream EGFR-STAT3-β-catenin signaling, overcome several limitations of earlier integrin inhibitors and represent a renewed therapeutic opportunity suited to the unique biology of CCA.

In this study, we addressed these gaps by integrating bulk transcriptomics (TCGA-CHOL), single-cell RNA sequencing datasets (GSE138709 and GSE142784), protein-level validation, mechanistic signaling assays in KRAS wild-type and KRAS-mutant CCA cell lines, and xenograft models to define the integrin αvβ3-EGFR-PD-L1 signaling axis in cholangiocarcinoma. Our findings identify integrin αvβ3 as a central pathogenic hub that links ECM remodeling, EGFR activation, STAT3/β-catenin signaling, and PD-L1-mediated immune modulation, and they demonstrate that DL-N2 derivatives inhibit these pathways through integrin-directed targeting 6, 34, 38-41.

Our study also explored the pathogenic role of integrin αvβ3 in cholangiocarcinoma and its potential as a therapeutic target. Our results prove that integrin αvβ3 was significantly upregulated in cholangiocarcinoma tissues compared to normal patients' samples **(Fig. 2 and 6)**. Integrin αvβ3 plays vital roles in cell adhesion, migration, and invasion, which are essential for tumor metastasis 10, 50. Integrin αvβ3 is known to interact with the extracellular matrix, promoting the metastatic spread of cancer cells 12. Patients with intrahepatic cholangiocarcinoma show an elevated level of lysyl oxidase-like 1 (LOXL1) in the tissues and sera compared to nontumor tissues and the sera of unaffected individuals 51. Overexpression of LOXL1 promotes cell proliferation, colony formation, and metastasis *in vivo* and *in vitro* and induced angiogenesis via the interaction with fibulin 5 (FBLN5) to bind with integrin αvβ3 and activate the FAK-MAPK signaling pathway inside vascular endothelial cells 51. Thyroid hormone binds to the extracellular domain of integrin αvβ3 on endothelial cells. It controls the transcription of specific vascular growth factor genes, regulates growth factor receptor/growth factor interactions, and stimulates endothelial cell migration to a vitronectin cue 52. On the other hand, in HMG-CoA reductase inhibitors, lovastatin inhibits the expression of integrin β3 and cell surface heterodimer integrin αvβ3 and downstream signaling, including FAK activation, and β-catenin, vimentin, ZO-1, and β-actin 53. The consequence downregulates the expressions of transforming growth factor (TGF)-β1, cyclooxygenase (COX)-2, and intercellular adhesion molecule (ICAM)-1 53 and affects cell adhesion 53. Integrin αvβ3 is upstream of EGFR to modulate EGFR-dependent activities **(Fig. 3)**. EGFR and integrin αv might work synergistically to promote cancer cell migration and invasion, key processes in tumor metastasis **(Fig. 9-10)**.

Our findings highlight the importance of integrin αvβ3 in cholangiocarcinoma progression and support the hypothesis that targeting integrin αvβ3 may potentially inhibit EGFR-dependent pathogenic effects via crosstalk effects. Therefore, targeting integrin αvβ3 could provide a novel therapeutic approach for this aggressive malignancy. EGFR has long been recognized as a key player in cancer progression, primarily due to its involvement in regulating cell proliferation, survival, and migration 14. We also observed a strong correlation between EGFR and integrin αvβ3 expression **(Fig. 2)**, suggesting that these two molecules may work synergistically to promote cholangiocarcinoma progression. Thyroxine via integrin αvβ3 **(Fig. 11)** and EGF via EGFR **(Fig. 12)** activated different signal transduction pathways in cholangiocarcinoma progression **(Fig. 9-10)** and immune modulation **(Fig. 4)**. Additionally, we demonstrated that modulating integrin αvβ3 activity by tetraiodothyroacetic acid (tetrac), a derivative of L-thyroxine (T_4_), inhibited EGFR-delivered signal pathways and activities 54. DL-N2 acts directly at the thyroid hormone receptor site located on the extracellular domain of the integrin αvβ3 heterodimer. Structural analyses show that this ligand-binding pocket is formed at the interface of the αv S1 domain and the β3 I-like domain, meaning that effective binding requires the intact αvβ3 complex. DL-N2 does not bind αv or β3 individually. Prior functional knockdown studies demonstrate that β3 plays the dominant signaling role because β3 silencing nearly abolishes tetrac- and nano-tetrac-mediated inhibition of downstream pathways, whereas αv knockdown produces only partial loss of responsiveness. These findings suggest that β3-driven signaling transduction is the primary determinant of DL-N2 tumor-suppressive activity, supporting our observation that DL-N2 suppresses both integrin-driven and EGFR-dependent signaling via pathway crosstalk 23, 24.

In addition to their roles in promoting tumor cell proliferation and survival, integrin αvβ3 and EGFR also play a critical role in modulating the immune microenvironment of cholangiocarcinoma. Our study revealed significant correlations between EGFR and immune cell infiltration, particularly CD4+ T cells and neutrophils **(Fig. 4C)**. This result suggests that EGFR may contribute to immune cell recruitment and activation in the tumor microenvironment, thereby influencing the immune landscape of cholangiocarcinoma. Similarly, integrin αvβ3 was found to correlate with the infiltration of neutrophils **(Fig. 4A-B)**, a type of immune cell known to play pro-inflammatory and anti-tumor roles in the tumor microenvironment. The interaction between EGFR, integrin αvβ3, and immune cells such as neutrophils and CD4+ T cells likely contributes to the cholangiocarcinoma immune evasion mechanisms **(Fig. 4)**. Previous studies have demonstrated that integrins can modulate immune cell recruitment, and EGFR signaling is known to influence immune cell activation and cytokine production. These findings suggest that integrin αvβ3 and EGFR contribute to the growth and metastasis of cholangiocarcinoma and play a significant role in shaping the immune response within the tumor microenvironment. Also, our single-cell transcriptomic profiling provided an essential layer of biological resolution that clarified the cellular sources and functional architecture of the integrin αvβ3-EGFR-PD-L1 axis in cholangiocarcinoma. By integrating two independent datasets (GSE138709 and GSE142784), we were able to dissect expression patterns across malignant epithelial cells, stromal fibroblast lineages, endothelial cells, and myeloid populations. The UMAP projections **(Fig. 5)** demonstrated that ITGAV and EGFR are predominantly confined to malignant and cholangiocyte-like clusters, supporting their role as tumor-intrinsic drivers of proliferation and epithelial plasticity. In contrast, ITGB3 was enriched within fibroblasts, myofibroblasts, and macrophage subsets, indicating that β3-dependent integrin signaling is largely supplied by the tumor stroma rather than the malignant epithelium. Notably, CD274 (PD-L1) localized to specific monocyte/macrophage subsets with additional expression in malignant cells, suggesting that immune checkpoint activity arises from both tumor-cell-intrinsic and tumor-associated macrophage (TAM) compartments. These observations were further supported by violin plot analyses **(Fig. 6)**, which confirmed that malignant cells contribute αv and EGFR, while stromal and immune cells provide β3 and PD-L1. This compartment-specific distribution suggests a cooperative signaling ecosystem in which malignant cells rely on integrin-activating ligands derived from CAFs, myofibroblasts, and TAMs to reinforce adhesion, extracellular matrix remodeling, and immune evasion. Importantly, CellChat modeling **(Fig. 7)** revealed a directional communication hierarchy dominated by stromal-to-malignant signaling. Fibroblast-derived ECM ligands (FN1, COL1A1, COL3A1, and THBS1) and macrophage-derived SPP1 emerged as the strongest initiators of integrin activation, directly engaging ITGAV/ITGB3 on malignant cells. Cholangiocyte-derived EGF and AREG provided complementary activation of EGFR, forming a dual-input system in which matrix-based and growth-factor-based signals converge on malignant epithelial clusters. These interactions provide a mechanistic explanation for why integrin αvβ3 and EGFR signaling remain persistently active in cholangiocarcinoma despite molecular heterogeneity. Taken together, the single-cell analyses refine the integrin αvβ3-EGFR-PD-L1 landscape by demonstrating that malignant epithelial cells form the core ITGAV/EGFR-high population, whereas stromal fibroblasts, myofibroblasts, and TAMs provide the β3- and PD-L1-rich microenvironmental cues that sustain tumor-stroma crosstalk and immune suppression. These findings indicate that integrin- and EGFR-dependent signaling in cholangiocarcinoma is not driven by a single cell population but instead emerges from coordinated interactions between malignant cells and the surrounding stroma. More importantly, the compartment-specific distribution of ITGAV, ITGB3, EGFR, and CD274 provides a mechanistic basis for understanding how upstream agonists, including growth factors and hormones, may differentially activate these pathways depending on the cellular context and KRAS mutation status.

In light of this, we next examined how thyroxine (T_4_) **(Fig. 11)** and EGF 35 stimulated cholangiocarcinoma growth, they activated different signal transduction pathways. Furthermore, they activated the differential effects in *KRAS* wild-type and *KRAS* mutant cholangiocarcinoma **(Fig. 11-12)**. Thyroxine and EGF induce different signaling pathways to stimulate the proliferation in *KRAS* Wild-Type SSP-25 cells and *KRAS* mutant HuCCT1 cells. Thyroxine primarily activated STAT3 and β-catenin signaling in *KRAS* mutant HuCCT1 cells, indicating that the *KRAS* mutation modulates the response to thyroxine **(Fig. 11)**. However, EGF induced the activation of ERK1/2, AKT, and STAT3 pathways in cholangiocarcinoma **(Fig. 12A)**. These findings highlighted the complexity of cholangiocarcinoma signaling and suggested that *KRAS* mutation influences the cellular signaling pathways activated by thyroxine (T_4_) and EGF and, further complicating the tumor's response to these molecules. On the other hand, Gene Set Enrichment Analysis (GSEA) indicated that the IL6/JAK/STAT3 signaling and IFN-γ response pathways were over-activated in cholangiocarcinoma patients **(Fig. 2F-I)**. Those clinical data and *in vitro* studies suggest that thyroxine (T_4_) and EGF may play vital roles in activating common signaling pathways to stimulate cancer cell proliferation and contribute to tumor immune modulation and inflammation. EGFR and T_4_ share common signaling pathways, indicating the availability of signaling crosstalk in stimulating tumor growth. These findings highlighted the complexity of cholangiocarcinoma signaling and suggested that *KRAS* mutation influences the cellular signaling pathways activated by thyroxine (T_4_) and EGF, further complicating the tumor's response to these molecules.

It has been reported that thyroxine-induced nuclear translocated PD-L1 plays a vital role in cancer cell proliferation 36. Further evidence indicates that nuclear PD-L1 promotes angiogenesis in malignancies 37. EGF treatment stimulated PD-L1 accumulation primarily in the cytosol of both SSP-25 and HuCCT1 cells **(Fig. 12B)**. However, EGF increased nuclear PD-L1 in KRAS wild-type SSP-25 cells **(Fig. 12B, left)**, but not in KRAS mutant HuCCT1 cells **(Fig. 12B, right)**. EGF binds to EGFR to activate β-catenin-mediated PD-L1 expression and promote immune evasion in glioblastoma 38. However, EGF treatment decreased non-p-β-catenin and total β-catenin levels observed in the nuclear fraction in SSP-25 cells **(Fig. 12, left)** but not in HuCCT1 cells **(Fig. 12, left)**. These results suggest that p-β-catenin increased in the nucleus in SSP-25 cells **(Fig. 12, left)**. Thus, EGF increased cytosolic PD-L1 expression, likely through β-catenin activation 39 in cholangiocarcinoma. However, T_4_ induces PD-L1 expression through STAT3 activation, translocates PD-L1 into the nucleus, and induces β-catenin expression in cancer cells 36. The different responses to T_4_ and EGF in *KRAS* mutant cholangiocarcinoma suggest that *KRAS* mutation status may influence the response to treatment with T_4_ or EGF, which could be considered when designing personalized therapeutic strategies for cholangiocarcinoma patients. Thus, our study demonstrates that integrin αvβ3 and EGFR play vital roles in cholangiocarcinoma progression, immune modulation, and immune evasion.

PD-L1 has emerged as a key immune checkpoint molecule in tumor immune evasion. Our study found that PD-L1 expression was significantly correlated with integrin αvβ3 expression in cholangiocarcinoma tissues **(Fig. 3)** and influences the infiltration levels of various immune cells **(Fig. 4)**. These findings are consistent with previous reports showing that PD-L1 expression in multiple cancers is associated with poor prognosis. PD-L1 binds to the PD-1 receptor on T cells, leading to T cell exhaustion and immune suppression. By upregulating PD-L1, cholangiocarcinoma cells can evade immune surveillance, allowing the tumor to grow and metastasize. Interestingly, PD-L1 knockdown experiments revealed that PD-L1 inhibition decreased p-β-catenin and active β-catenin expression in cancer cell proliferation 36. This result suggests that PD-L1 regulates the β-catenin signaling pathway and highlights the complexity of immune regulation in cholangiocarcinoma. *KRAS* mutations have been shown to alter the signaling pathways involved in immune modulation, which may influence the response to PD-L1 blockade. These findings provide further insight into the potential of targeting PD-L1 as an immune checkpoint inhibitor in cholangiocarcinoma therapy. These findings have important therapeutic implications, particularly for developing combination therapies that target EGFR, integrin αvβ3, and PD-L1. Alternatively, targeting integrin αvβ3 by tetrac and its nano-derivative blocks PD-L1 expression in *KRAS* mutant cancer cells 38 underscores the importance of considering *KRAS* mutation status when designing immune-based therapies.

Integrin αvβ3 antagonist, NDAT has been shown to induce antiangiogenic actions, including disruption of crosstalk between integrin αvβ3 and adjacent cell surface vascular growth factor receptors, resulting in disordered vascular endothelial growth factor (VEGF) and basic fibroblast growth factor (bFGF; FGF2) actions at their respective plasma membrane receptors 52. NDAT also downregulates the expression of VEGFA and EGFR genes, upregulates transcription of the angiogenesis suppressor gene, thrombospondin 1 (THBS1; TSP1), and decreases the cellular abundance of Ang-2 protein and matrix metalloproteinase-9 52. Inhibition of integrin αvβ3 by tetrac derivatives further enhances the anti-proliferation induced by the EGFR inhibitor, gefitinib, in KRAS mutant cancer cells. DL-N2 and DL-N2-Dox inhibit cell proliferation in cholangiocarcinoma *in vitro* **(Fig. 13)**. In addition, DL-N2 payload with doxorubicin (Dox) suppressed cancer proliferation in cholangiocarcinoma, especially in Ras wild-type SSP-25 cells. DL-N2 and DL-N2-Dox also inhibited tumor growth in cholangiocarcinoma xenografted mice **(Fig. 14)**.

One indicator of drug-induced cytotoxicity is body weight loss when the drug is applied in a xenograft. DL-N2 and DL-N2-Dox effectively suppressed tumor growth and prevented weight loss, demonstrating their biosafety in animals **(Fig. 14)**. Similar observations are obtained in other tetrac-derivatives 55. Similar observations are obtained in other tetrac-derivatives 32. DL-N2, DL-N2-Dox, and Lipo-Dox reduce xenograft tumor growth in cholangiocarcinoma TFK-1 cells xenografted mice. Treatment of DL-N2 or DL-N2-Dox reduced the growing sizes of tumors compared to those of control and Lipo-Dox-treated groups **(Fig. 14)**. In addition, DL-N2 and DL-N2-Dox also significantly reduced tumor growth after one week of treatment **(Fig. 14B)**. Those observations suggest that the tumor-specific targeting effect against integrin αvβ3 is vital in inhibiting cancer cell growth. However, DL-N2-Dox showed more tumor size reduction than DL-N2 after four weeks of treatment **(Fig. 14F)**, suggesting that the cytotoxic effect of Dox induces a more cancer-killing effect than tetrac. It is not surprising to obtain such results since Lipo-Dox is more effective than DL-N2 *in vitro* studies in killing cancer cells **(Fig. 13)**. However, the harvested tumor sizes from DL-N2 and Lipo-Dox-treated mice were not significantly different, confirming our hypothesis that tetrac-derivatives inhibit angiogenesis via growth factors such as EGF 56. DL-N2 derivatives resulted in the suppression of cholangiocarcinoma tumor growth in a xenograft model **(Fig. 14)**. These results confirmed our previous observation that nano-tetrac binds to integrin αvβ3 to inhibit integrin αvβ3 and EGF-dependent signal transduction in KRAS mutant cancer cells *in vitro* and *in vivo* 38, 54. The EGFR inhibitor, gefitinib, does not inhibit PD-L1 expression and proliferation in the *KRAS* mutant cancer cell line 32. However, tetrac nanoparticulate derivative inhibits PI3K activation, PD-L1 accumulation, and cell growth in gefitinib-resistant cancer cells 32. These observations indicate that blocking the integrin αvβ3-dependent signal transduction pathway can inhibit the EGFR-dependent signal pathway via crosstalk. Although DL-N2 derivatives and Lipo-Dox reduce xenograft tumor weights **(Fig. 14F)**, DL-N2-Dox significantly reduced tumor growth compared to DL-N2 and Lipo-Dox **(Fig. 14B)**. On the other hand, DL-N2-Dox was more efficient than Lipo-Dox, demonstrating that integrin αvβ3-targeted DL-N2-Dox was more efficient than untargeted Lipo-Dox in cancer treatment. Tetrac or NDAT have been shown to facilitate EGFR inhibitors such as cetuximab (Erbitux) 57 and gefitinib 32-induced antiproliferation in *RAS* mutant cancer cells.

While this study provides valuable insights into the roles of integrin αvβ3, EGFR, and their agonists in cholangiocarcinoma, limitations must be addressed in future research. First, the clinical evidence is too scarce and incomplete to draw a solid conclusion. Future studies of patient-derived xenograft (PDX) models to validate further the molecular mechanisms this study identified are urgently needed. Second, while our bioinformatic analysis provided valuable insights into the expression patterns of EGFR, integrin αvβ3, and PD-L1, additional *in vivo* studies are crucial to fully understand the impact of these biomarkers on tumor growth and immune evasion in cholangiocarcinoma. Lastly, combining immune checkpoint inhibitors with EGFR or integrin-targeted therapies must be explored in clinical trials. The findings from this study lay the groundwork for future therapeutic strategies that could improve patient outcomes in cholangiocarcinoma, particularly in patients with KRAS mutations or advanced disease stages.

**Figure 15:**
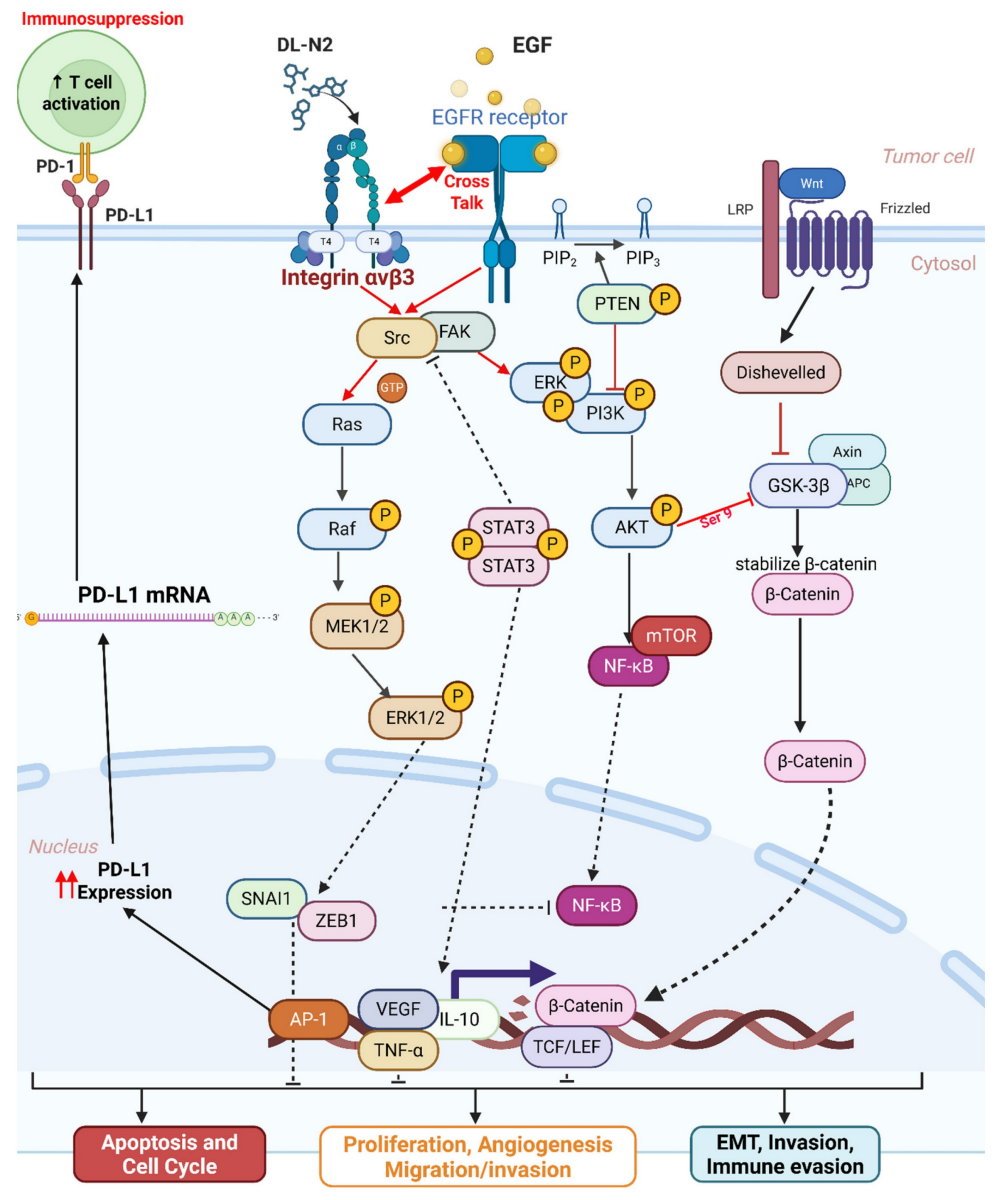
Unified mechanistic framework of the integrin αvβ3-EGFR-SRC axis driving STAT3/AKT signaling, β-catenin stabilization, EMT, cytokine production, and PD-L1-mediated immune evasion. This schematic illustrates how integrin αvβ3 and EGFR cooperate to amplify oncogenic signaling in tumor cells. Thyroxine (T_4_) binds integrin αvβ3 and promotes recruitment and activation of FAK and SRC, which engage the Ras-Raf-MEK-ERK cascade.

In conclusion, integrin αvβ3 and EGFR play essential roles in cell proliferation and progression in cholangiocarcinoma **(Fig. 15)**. Thyroxine and EGF stimulate signal transduction and activate gene expression, cell proliferation, and metastasis via integrin αvβ3 and EGFR, respectively. Notably, there is a crosstalk between these pathways. Both EGF and thyroxine induced PD-L1 expression. Thyroxine-induced PD-L1 involves cancer cell proliferation and surface PD-L1 presentation. However, EGF-induced PD-L1 plays a role in cell surface presentation for immune surveillance. DL-N2 and DL-N2-Dox inhibit integrin αvβ3-dependent cell activities and block EGFR-signaling via crosstalk **(Fig. 15)**. Furthermore, cholangiocarcinoma xenograft studies suggest their therapeutic potential against cholangiocarcinoma.

## Supplementary Material

Supplementary figures and tables.

## Data Availability

Some of the data were obtained from publicly databases. All other data are available from the corresponding author upon reasonable request.

## Abbreviations

CCA: Cholangiocarcinoma
EGF: Epidermal Growth Factor
T_4_: Thyroxine
PD-L1: Programmed Death-Ligand 1
EGFR: Epidermal Growth Factor Receptor
KRAS: Kirsten Rat Sarcoma Viral Oncogene Homolog
PPI: Protein-Protein Interaction
TCGA: The Cancer Genome Atlas
MetaCore: MetaCore Pathway Analysis Platform
UALCAN: University of Alabama at Birmingham Cancer Data Analysis Portal
GEPIA2: Gene Expression Profiling Interactive Analysis 2
FAK: Focal Adhesion Kinase
STRING: Search Tool for the Retrieval of Interacting Genes/Proteins
TME: Tumor Microenvironment
CD4+: Cluster of Differentiation 4 (T-helper cells)
CD8+: Cluster of Differentiation 8 (Cytotoxic T cells)
KRAS-WT: Wild-Type KRAS
KRAS-Mut: Mutant KRAS
T_4_R: Thyroxine Receptor
C8T: Cytotoxic T cells
HCC: Hepatocellular Carcinoma
HIF-1α: Hypoxia-Inducible Factor 1-alpha
mTOR: Mechanistic Target of Rapamycin
PI3K: Phosphoinositide 3-kinase
MEK: Mitogen-Activated Protein Kinase Kinase
ERK: Extracellular Signal-Regulated Kinase
p-ERK: Phosphorylated Extracellular Signal-Regulated Kinase
p-FAK: Phosphorylated Focal Adhesion Kinase
TGF-β: Transforming Growth Factor Beta
IL-6: Interleukin 6
IFN-γ: Interferon Gamma
MMP: Matrix Metalloproteinase
NK-κB: Nuclear Factor Kappa B
MAPK: Mitogen-Activated protein Kinase
FasL: Fas Ligand
TNF-α: Tumor Necrosis Factor Alpha
