# Supplementary material for: Integrin αvβ3 is a Potential Therapeutic Target in Cholangiocarcinoma
Source: Int J Med Sci. 2026 Feb 4;23(3):889–915. doi: 10.7150/ijms.125066 (PMC12964564; doi:10.7150/ijms.125066)
Supplement: Supplementary file 1 — Supplementary figures and tables. [file ijmsv23p0889s1.pdf]

Supplementary data

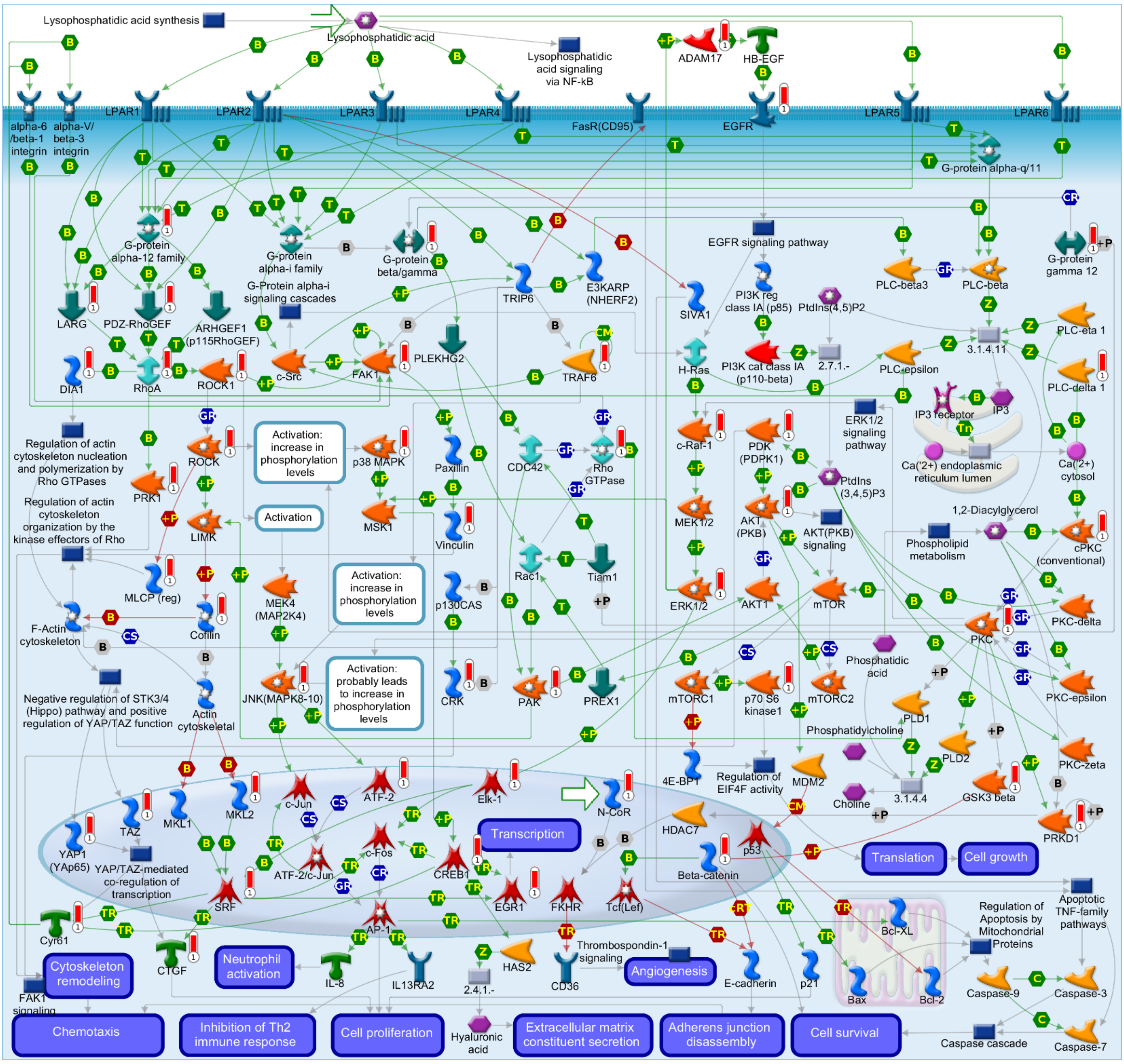

Supplementary Figure S1. MetaCore signaling diagram showing enrichment of the Chemotaxis_Lysophosphatidic Acid (LPA) Signaling via GPCRs" pathway associated with ITGAV expression in cholangiocarcinoma.

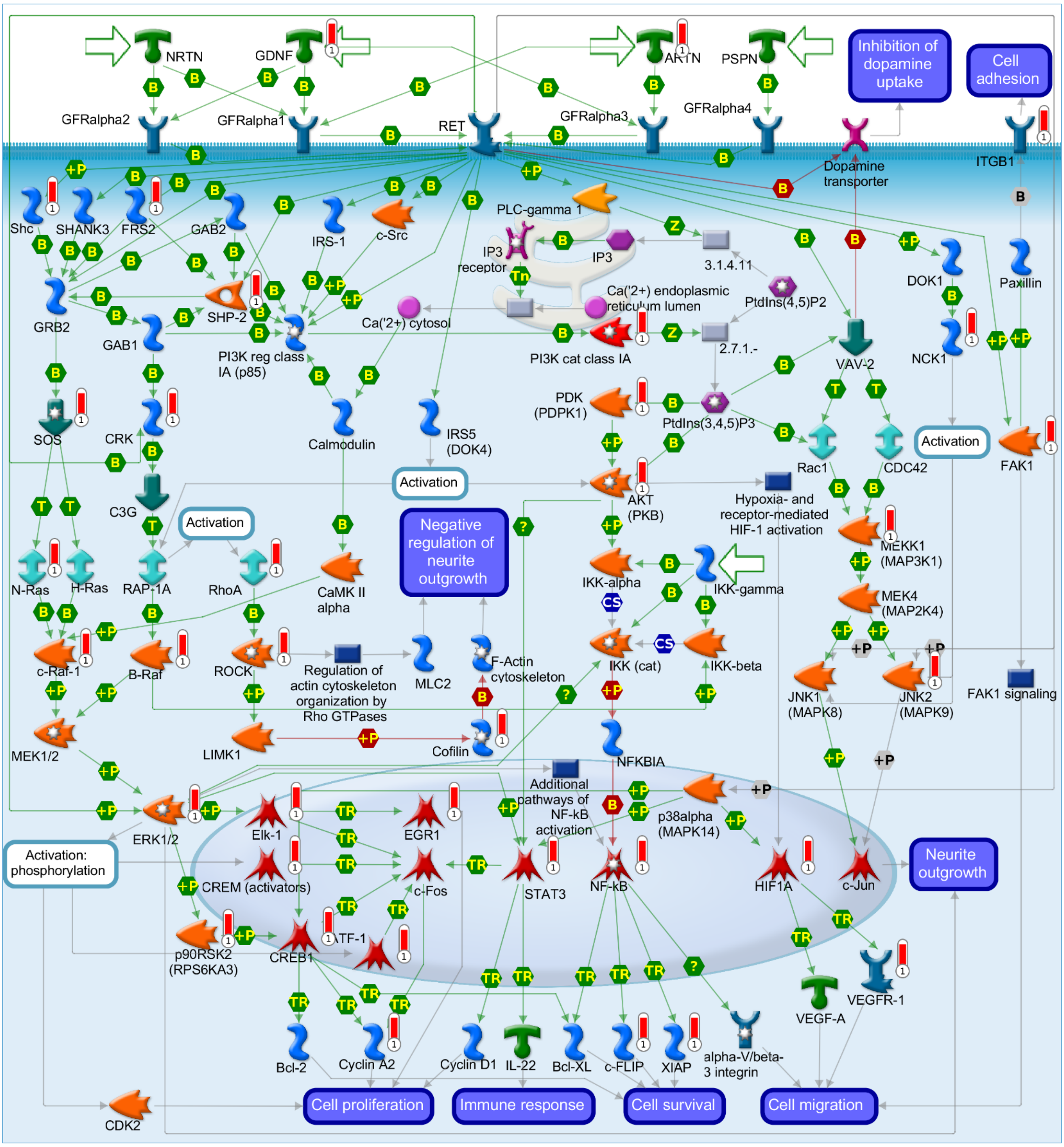

Supplementary Figure S2. MetaCore signaling diagram showing enrichment of the Development_The role of GDNF ligand family RET receptor in cell survival, growth and proliferation pathway associated with ITGAV expression in cholangiocarcinoma.

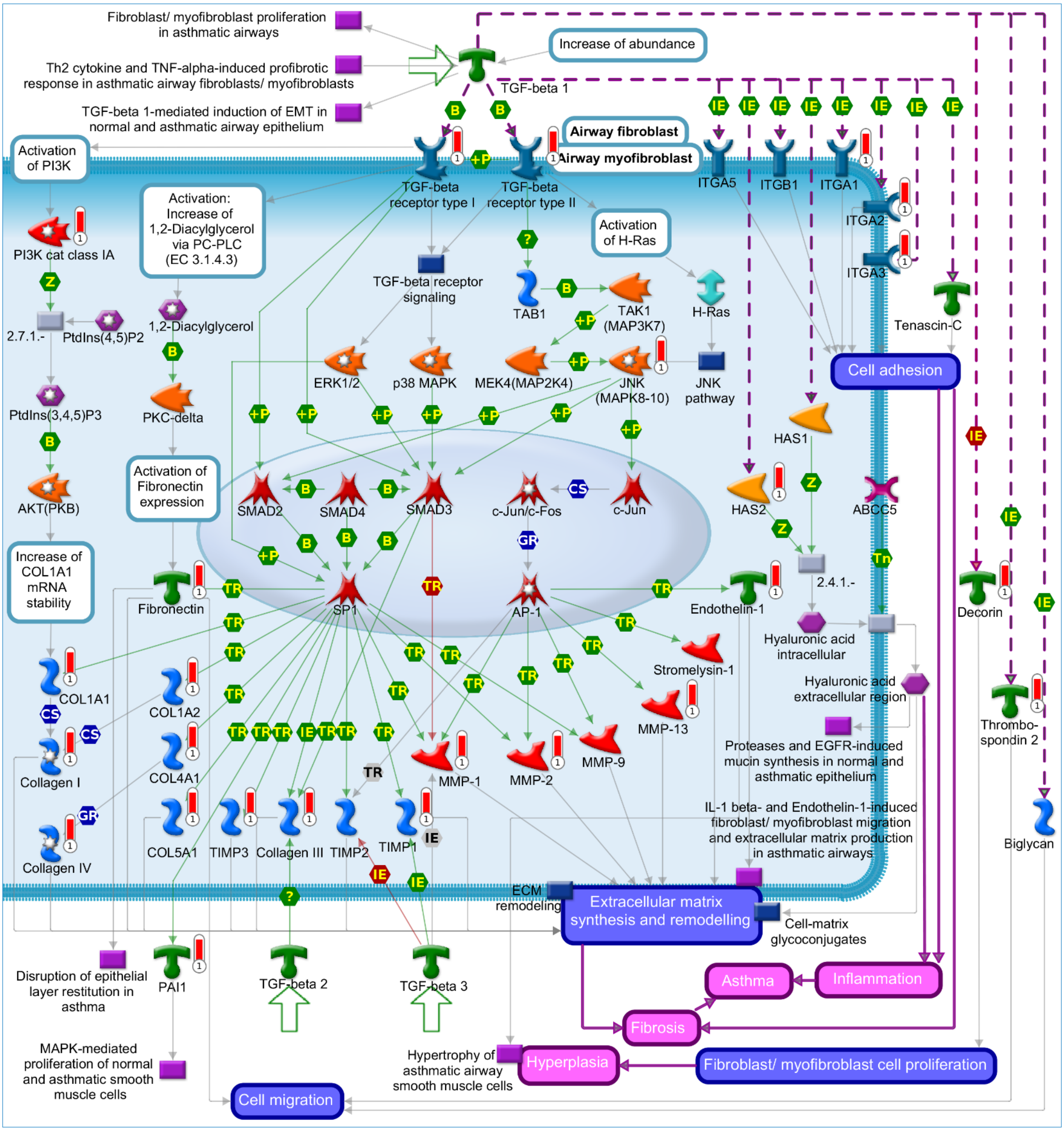

Supplementary Figure S3. MetaCore signaling diagram showing enrichment of TGF-beta-induced fibroblast myofibroblast migration and extracellular matrix production in asthmatic airways pathway associated with ITGB3 expression in cholangiocarcinoma.

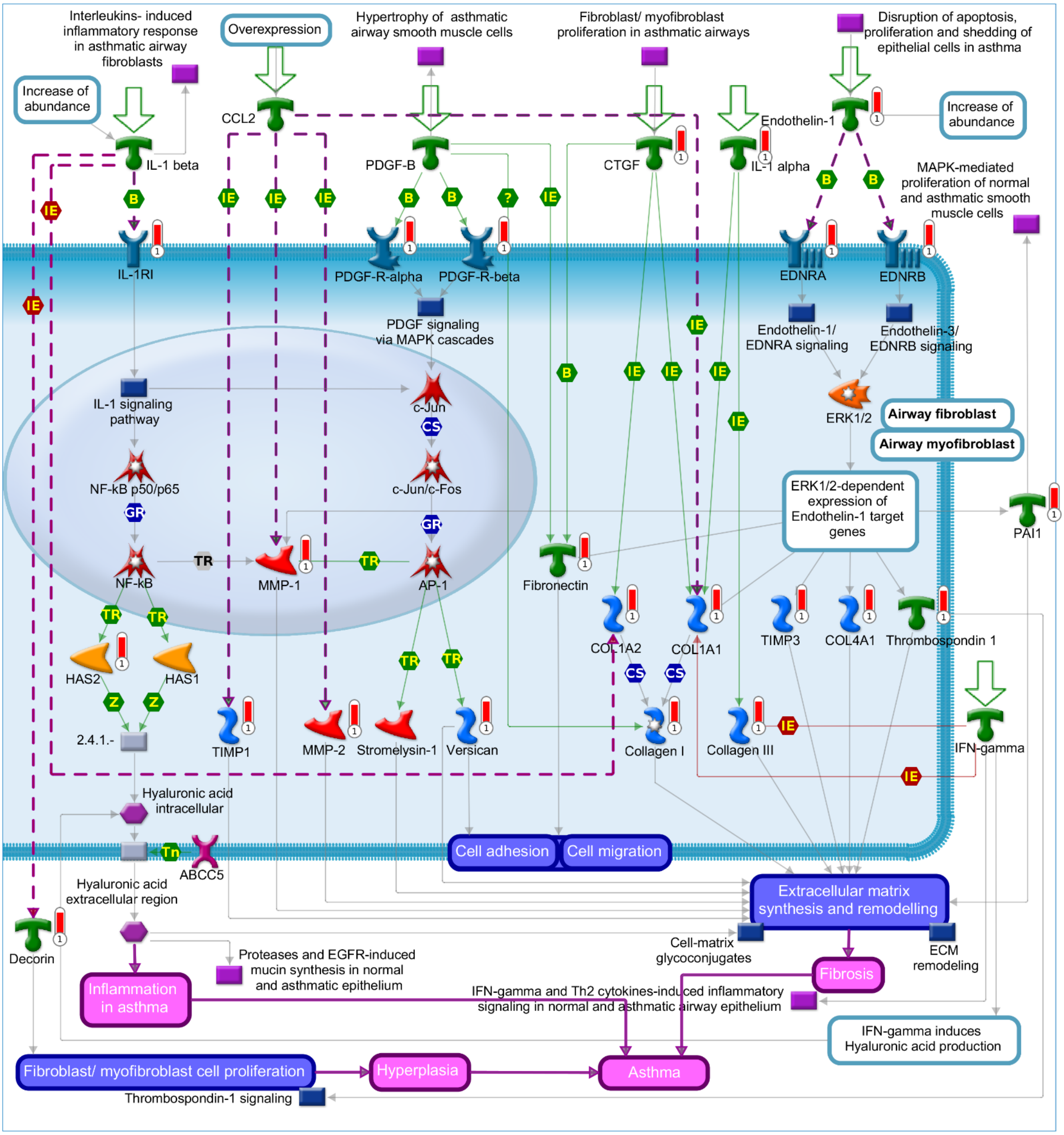

Supplementary Figure S4. MetaCore signaling diagram showing enrichment IL-1 beta- and Endothelin-1-induced fibroblast myofibroblast migration and extracellular matrix production in asthmatic airways pathway associated with ITGB3 expression in cholangiocarcinoma.

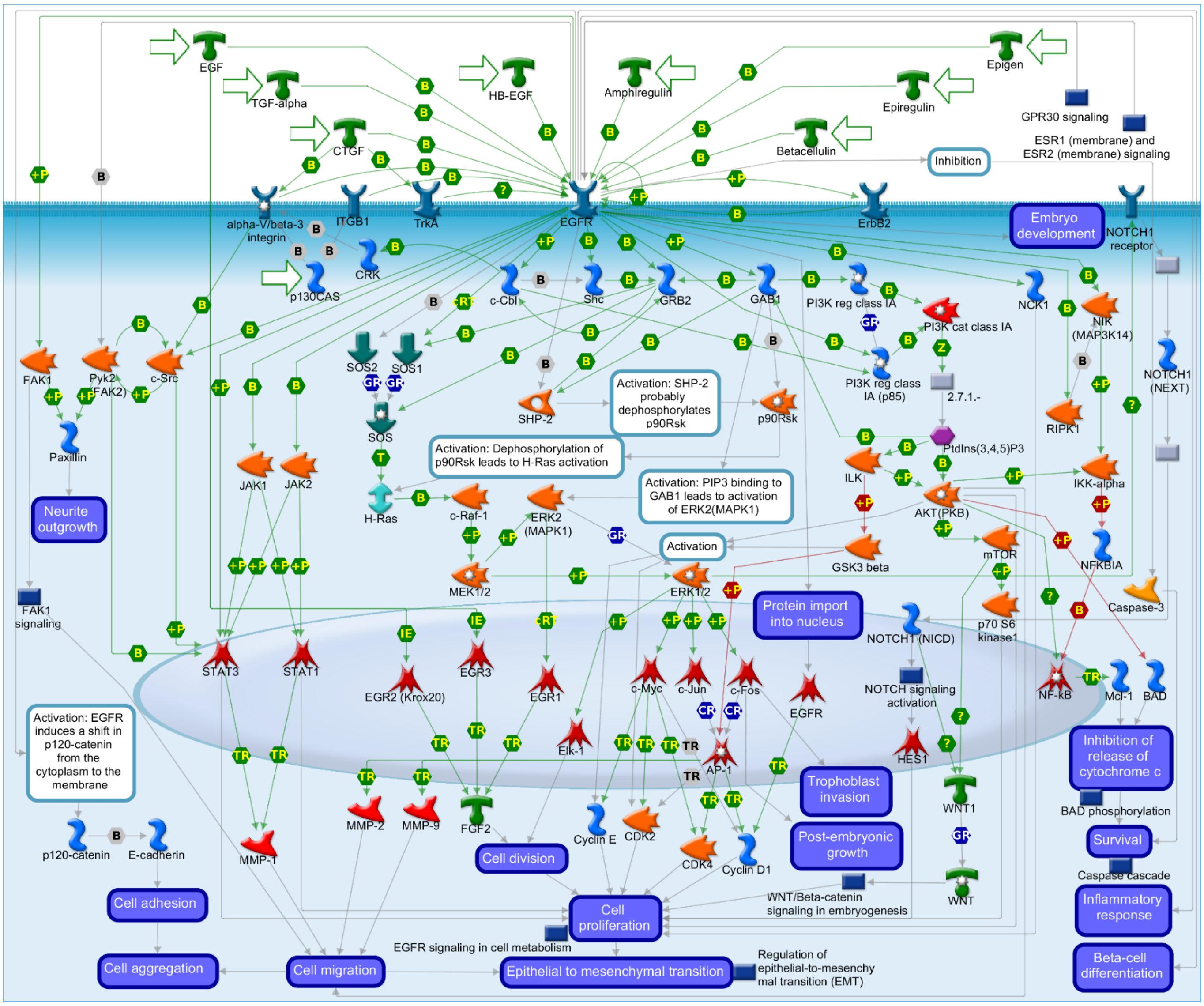

Supplementary Figure S5. MetaCore signaling diagram showing enrichment of Development_EGFR signaling pathway associated with EGFR expression in cholangiocarcinoma.

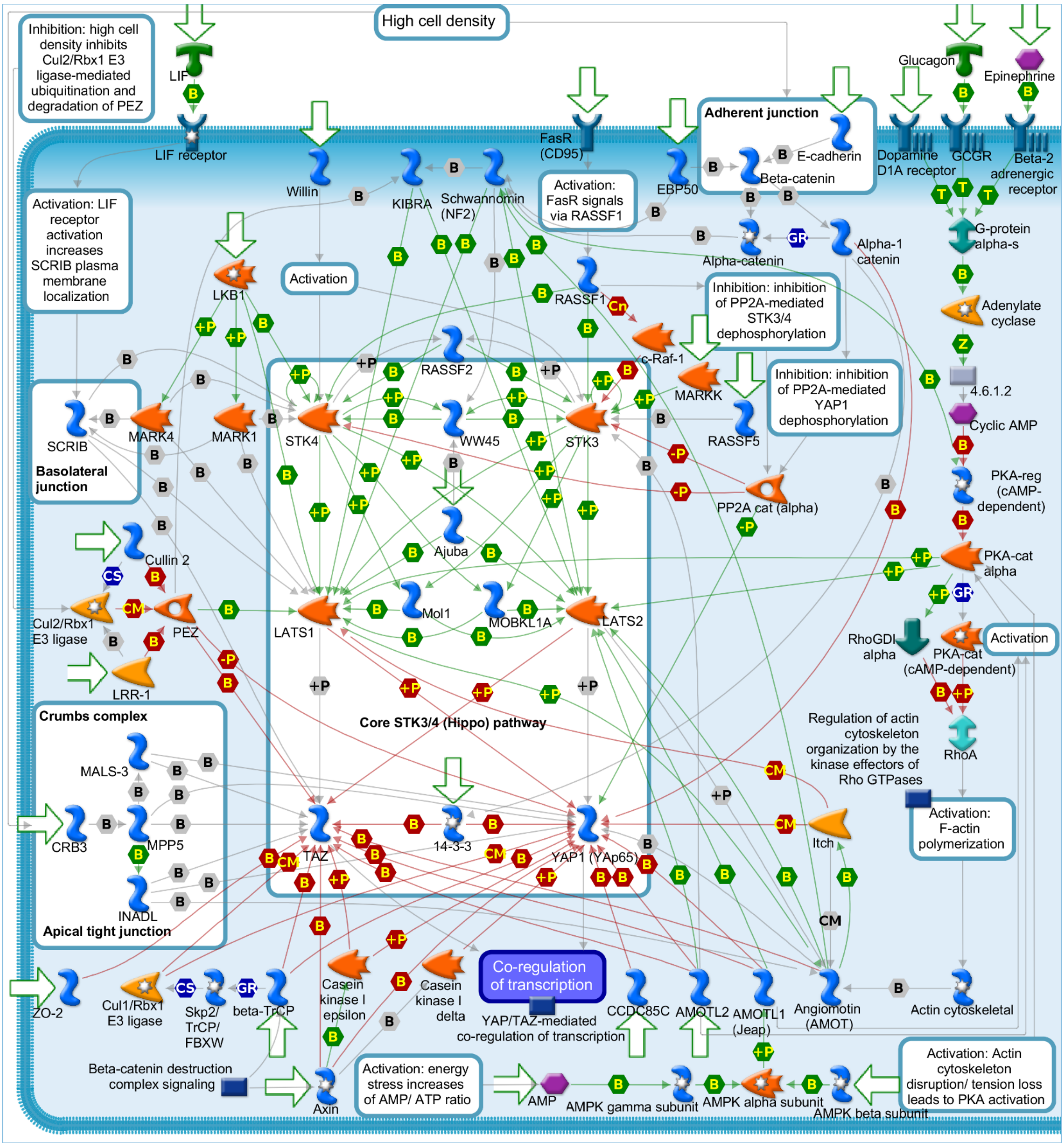

Supplementary Figure S6. MetaCore signaling diagram showing enrichment of Development_Positive regulation of STK34 (Hippo) pathway and negative regulation of YAPTAZ function pathway associated with EGFR expression in cholangiocarcinoma.

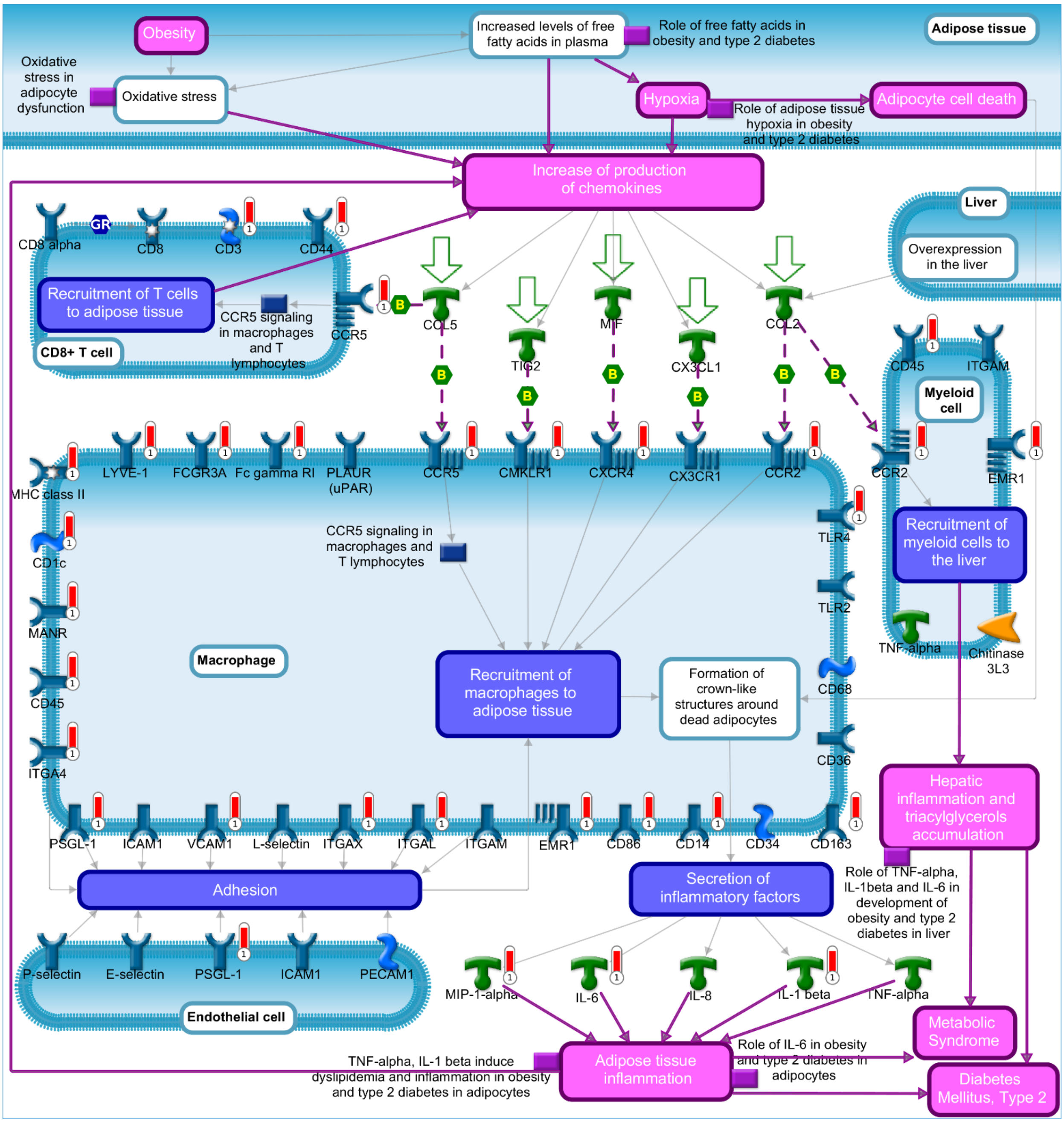

Supplementary Figure 7. MetaCore signaling diagram showing enrichment of Chemokines in inflammation in adipose tissue and liver in obesity, type 2 diabetes and metabolic syndrome pathway associated with CD724 expression in cholangiocarcinoma.

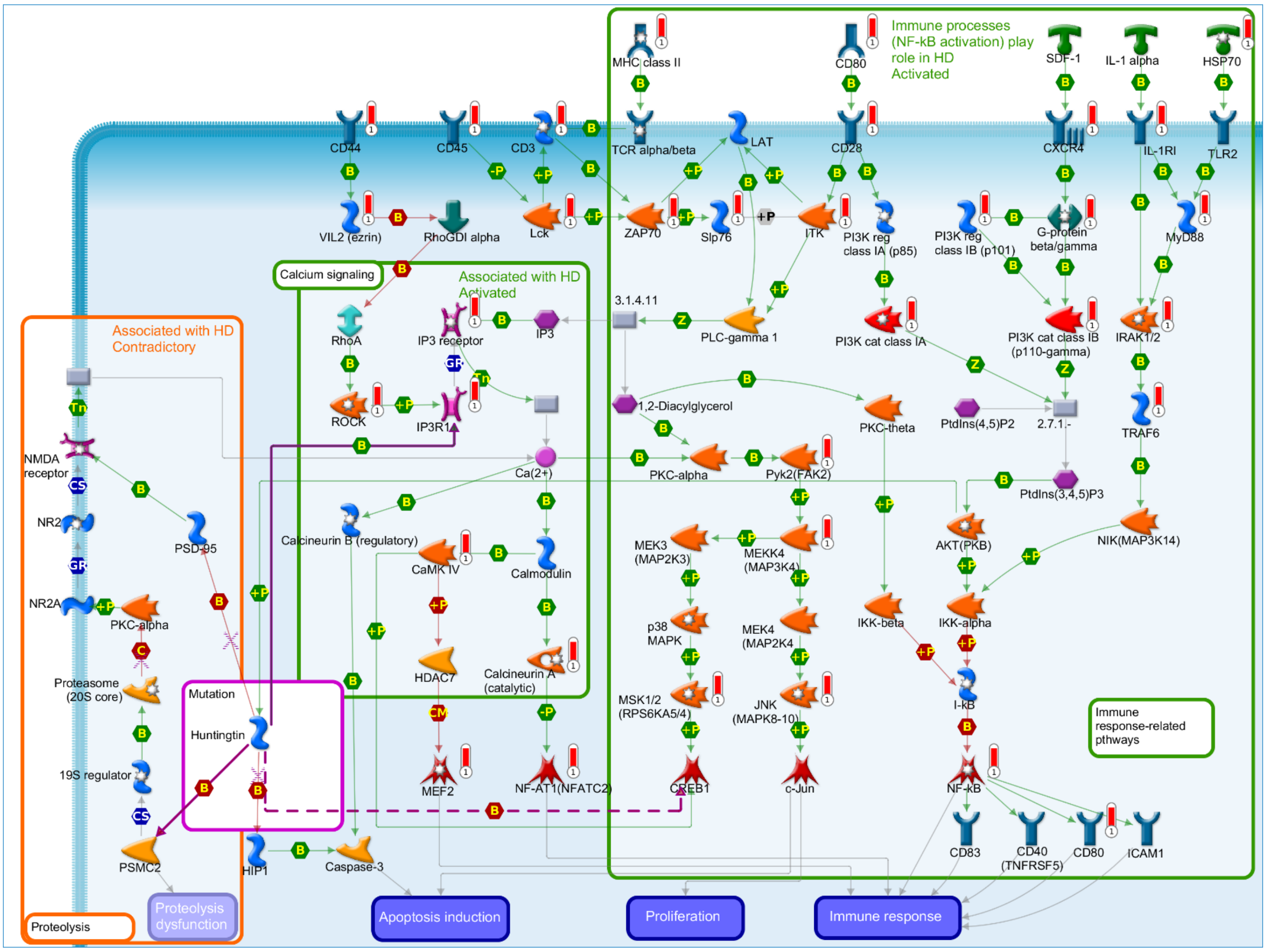

Supplementary Figure S8. MetaCore signaling diagram showing enrichment of CHDI_Correlations from Replication data_Causal network (positive correlations) pathway associated with CD724 expression in cholangiocarcinoma.

Table S_1.1: Protein–protein interaction (PPI): Node 1 and Node 2 represent the interacting protein pairs, while the Score indicates the STRING confidence score (from 0 to 1)

Node 1	***Node 2***	***Score***
AKT1	CD274	0.72
AKT1	EGFR	0.792
AKT1	ITGAV	0.472
AKT1	ITGB3	0.781
AKT1	MAPK1	0.988
AKT1	MAPK3	0.957
AKT1	SRC	0.995
AKT1	STAT3	0.966
EGFR	AKT1	0.792
EGFR	CD274	0.87
EGFR	ITGAV	0.686
EGFR	ITGB3	0.65
EGFR	MAPK1	0.612
EGFR	MAPK3	0.528
EGFR	SRC	0.999
EGFR	STAT3	0.998
MAPK1	AKT1	0.988
MAPK1	EGFR	0.612
MAPK1	ITGAV	0.651
MAPK1	ITGB3	0.728
MAPK1	MAPK3	0.994
MAPK1	SRC	0.932
MAPK1	STAT3	0.996
MAPK3	AKT1	0.957
MAPK3	CD274	0.48
MAPK3	EGFR	0.528
MAPK3	ITGAV	0.551
MAPK3	ITGB3	0.758
MAPK3	MAPK1	0.994
MAPK3	SRC	0.944
MAPK3	STAT3	0.87
SRC	AKT1	0.995
SRC	CD274	0.545
SRC	EGFR	0.999
SRC	ITGAV	0.946
SRC	ITGB3	0.998
SRC	MAPK1	0.932

SRC	MAPK3	0.944
SRC	STAT3	0.999
STAT3	AKT1	0.966
STAT3	CD274	0.992
STAT3	EGFR	0.998
STAT3	ITGAV	0.516
STAT3	MAPK1	0.996
STAT3	MAPK3	0.87
STAT3	SRC	0.999

Table S_2: Pathway analysis of gene co-expressed ITGAV (GDC Dataset) from the MetaCore database (with p-value< 0.05 set as the cutoff value).

S.No.	Maps	pValue	Min FDR	Network Objects from Active Data
1	Development_Positive regulation of WNT/Beta-catenin signaling in the cytoplasm	2.627E-15	3.869E-12	PPP2R2A, HECTD1, ITGB1, Bcl-9, Trabid, BIG1, Tankyrases, BIG2, USP9X, USP25, UBE2B, WNT, APC protein, Alpha-1 catenin, FAK1, 14-3-3, GSK3 alpha/beta, IGF-1 receptor, Beta-catenin, PKA-reg type II (cAMP-dependent), JNK(MAPK8-10), PP2A catalytic, ERK2 (MAPK1), AKT(PKB), Makorin-1, Dsh, PKA-cat (cAMP-dependent), USP47, ILK, Frizzled, SET7, ZBED3, YAP1 (YAp65), DOCK4, DACT1
2	Chemotaxis_Lysophosphatidic acid signaling via GPCRs	5.213E-13	3.839E-10	RhoA, MLCP (reg), ROCK, PRKD1, cPKC (conventional), G-protein alpha-12 family, GSK3 beta, PAK, p70 S6 kinase1, PDZ-RhoGEF, PKC, Vinculin, ATF-2, DIA1, EGFR, TRAF6, PLC-delta 1, LARG, ROCK1, MKL2, ERK1/2, Cyr61, PRK1, FAK1, CREB1, Beta-catenin, EGR1, G-protein beta/gamma, Rho GTPase, N-CoR, TAZ, CRK, JNK(MAPK8-10), c-Raf-1, CTGF, AKT(PKB), PDK (PDPK1), ADAM17, p38 MAPK, Elk-1, SRF, Cofilin, YAP1 (YAp65), G-protein gamma 12
3	Development_The role of GDNF ligand family/ RET receptor in cell survival, growth and proliferation	2.674E-12	1.082E-09	STAT3, RhoA, GDNF, ITGB1, Shc, ROCK, PI3K cat class IA, B-Raf, CREM (activators), NCK1, Cyclin A2, MEKK1(MAP3K1), HIF1A, SHP-2, N-Ras, XIAP, ATF-1, ERK1/2, FAK1, CREB1, EGR1, VEGFR-1, p90RSK2(RPS6KA3), CRK, c-Raf-1, JNK2(MAPK9), SOS, NF-kB, c-FLIP, FRS2, AKT(PKB), PDK (PDPK1), ARTN, Elk-1, Cofilin
4	Glucocorticoid-induced elevation of intraocular pressure as glaucoma risk factor	2.937E-12	1.082E-09	TRIO, RhoA, ITGB1, ROCK, PI3K cat class IA, ROR2, COL4A1, C/EBP zeta, GCR Beta, GCR, PLAT (TPA), Filamin B (TABP), EGFR, SERPINA3 (ACT), WNT2, FAK1, SENP1, Alpha-actinin, GCR Alpha, Filamin A, LAMB3, Collagen IV, Thrombospondin 1, HGF receptor (Met), Fibronectin, PAI1, LAMA1, MLCK
5	Signal transduction_Calcium-mediated signaling	6.303E-12	1.681E-09	RhoA, MLCP (reg), ROCK, cPKC (conventional), MARK2, RelA (p65 NF-kB subunit), Calcineurin A (catalytic), PKC, NUR77, MYH11, PKC-alpha, ATF-2, G6PT, ERK1/2, 14-3-3, CaMK I, CREB1, EGR1, ACTA2, JNK(MAPK8-10), HDAC4, MEF2, NURR1, NF-kB, HDAC5, AKT(PKB), p300, p38 MAPK, Elk-1, SRF
6	Development_Negative regulation of WNT/Beta-catenin signaling in the cytoplasm	7.022E-12	1.681E-09	ELAVL1 (HuR), KLHL12, WWP1, HECTD1, PEG3, c-Cbl, PKC-alpha, STK3, WNT, HUWE1, APC protein, Amer1, Itch, Alpha-1 catenin, Beclin 1, GSK3 alpha/beta, LATS2, Beta-catenin, G-protein beta/gamma, WDR26, Skp2/TrCP/FBXW, TAZ, PP2A catalytic, PI3K cat class III (Vps34), Dsh, RNF185,

				Casein kinase I alpha, Frizzled, YAP1/TAZ, YAP1 (YAp65), G-protein alpha-13, DACT1
**7**	Role of stellate cells in progression of pancreatic cancer	7.990E-12	1.681E-09	COL1A1, Shc, PI3K cat class IA, RECK, RelA (p65 NF-kB subunit), PDGF-B, EGFR, ERK1/2, FAK1, ACTA2, alpha-5/beta-1 integrin, c-Raf-1, SOS, COL1A2, CTGF, PDGF-R-alpha, AKT(PKB), PDGF receptor, NF-kB p50/p65, OSF-2, PDK (PDPK1), Fibronectin, Collagen I, TGF-beta receptor type I, PDGF-R-beta, NF-kB p65/p65, Collagen III
**8**	Neurogenesis_NGF/ TrkA MAPK-mediated signaling	9.420E-12	1.734E-09	SPHK1, Sequestosome 1(p62), Shc, MAPKAPK2, B-Raf, NUR77, NF-kB1 (p50), K-RAS, PKC-lambda/iota, SHP-2, N-Ras, MAP2K5 (MEK5), ERK1/2, KIDINS220, CREB1, CrkL, EGR1, SUR-8, PP2A regulatory, M-Ras, PKA-reg (cAMP-dependent), CRK, c-Raf-1, SOS, PP2A catalytic, FRS2, SP1, RGS2, PVR, JMJD3, PDZ-GEF1, p38 MAPK, Elk-1, KCTD11, PKA-cat (cAMP-dependent), SRF, p90Rsk
**9**	Signal transduction_ESR1 (membrane) and ESR2 (membrane) signaling	1.384E-11	2.265E-09	RhoA, SREBP1 precursor, Shc, mGluR1, CBP, cPKC (conventional), p120GAP, PI3K cat class IA, Protein kinase G1, GSK3 beta, PKC, ROCK2, PKC-alpha, MSN (moesin), EGFR, N-WASP, Adenylate cyclase, ERK1/2, p190RhoGAP, FAK1, CREB1, IGF-1 receptor, Beta-catenin, EGR1, G-protein beta/gamma, ChREBP, PKA-reg (cAMP-dependent), c-Raf-1, SOS, Striatin, AKT(PKB), PDK (PDPK1), Elk-1, PKA-cat (cAMP-dependent), SRF, p90Rsk, Cofilin, G-protein alpha-13, PR (nuclear)
**10**	Stellate cells activation and liver fibrosis	1.681E-11	2.477E-09	COL1A1, Shc, PI3K cat class IA, GSK3 beta, PDGF-B, PTCH1, ICAM1, MyD88, TRAF6, IL-1RI, Beta-catenin, ACTA2, IRAK1/2, c-Raf-1, SOS, COL1A2, PDGF-R-alpha, ERK2 (MAPK1), AKT(PKB), SP1, PDGF receptor, NF-kB p50/p65, SARA, Dsh, ERK1 (MAPK3), Elk-1, Frizzled, TGF-beta receptor type I, PDGF-R-beta
**11**	TGF-beta 1-induced transactivation of membrane receptors signaling in hepatocellular carcinoma (HCC)	2.105E-11	2.667E-09	ITGB1, PI3K cat class IA, GSK3 beta, ITGA2, PTEN, SLUG, DOCK1, FAK1, c-Abl, alpha-2/beta-1 integrin, Beta-catenin, Cyclin A, alpha-5/beta-1 integrin, CRK, PDGF-R-alpha, AKT(PKB), PDGF receptor, ITGA5, Fibronectin, DDX5, Actin, Cofilin, TGF-beta receptor type I, PDGF-R-beta
**12**	G-protein signaling_RhoB activation	2.172E-11	2.667E-09	ELAVL1 (HuR), KIF13A, p190-RhoGEF, PDZ-RhoGEF, PDGF-B, GGTase-I, GCR, HIF1A, ATF-2, SMURF1, EGFR, ATM, LARG, ERK1/2, ARHGEF10, JNK(MAPK8-10), AKT(PKB), TIM1, p164-RhoGEF, ATR, p300, HGF receptor

				(Met), p38 MAPK, NFYA, TGF-beta receptor type I, PDGF-R-beta
**13**	Cell adhesion_Integrin-mediated cell adhesion and migration	3.250E-11	3.683E-09	Tensin, Talin, RhoA, ITGB1, p190-RhoGEF, PARD3, PKC, ICAM1, Vinculin, PKC-lambda/iota, PINCH, Zyxin, DOCK1, LARG, FAK1, alpha-2/beta-1 integrin, alpha-5/beta-1 integrin, Alpha-parvin, Alpha-actinin, CRK, GIT2, alpha-1/beta-1 integrin, Collagen IV, Fibronectin, ILK, Collagen I, Collagen III
**14**	IL-1 beta- and Endothelin-1-induced fibroblast/ myofibroblast migration and extracellular matrix production in asthmatic airways	4.305E-11	4.530E-09	COL1A1, COL4A1, EDNRB, PDGF-B, TIMP3, ERK1/2, Versican, IL-1RI, COL1A2, NF-kB, CTGF, PDGF-R-alpha, ABCC5, NF-kB p50/p65, Thrombospondin 1, Fibronectin, PAI1, EDNRA, Collagen I, PDGF-R-beta, Collagen III
**15**	Apoptosis and survival_NGF/ TrkA PI3K-mediated signaling	4.815E-11	4.728E-09	TRIO, RhoA, MLCP (reg), Shc, ROCK, PI3K cat class IA, MRCKalpha, GSK3 beta, PARD3, FOXO4, p70 S6 kinase1, Calcineurin A (catalytic), MSN (moesin), CLCN4, N-WASP, APC protein, PAK2, DOCK1, ERK1/2, p190RhoGAP, KIDINS220, CREB1, SOS, AKT(PKB), PDK (PDPK1), ARAP3, SSH1L, ILK, Cofilin, Tubulin (in microtubules)
**16**	Development_VEGF signaling via VEGFR2 - generic cascades	1.005E-10	8.833E-09	SPHK1, RhoA, Shc, COX-1 (PTGS1), p120GAP, PI3K cat class IA, GSK3 beta, MAPKAPK2, Calcineurin A (catalytic), PKC, NCK1, MEKK1(MAP3K1), Vinculin, PKC-alpha, PAK2, ROCK1, ERK1/2, FAK1, CREB1, Neurofibromin, Beta-catenin, Fyn, c-Raf-1, SOS, ERK2 (MAPK1), AKT(PKB), HSP90, NF-kB p50/p65, PDK (PDPK1), p38 MAPK, ERK1 (MAPK3), p90Rsk, MLCK
**17**	Signal transduction_PDGF signaling via PI3K/AKT and NFkB pathways	1.019E-10	8.833E-09	PI3K cat class IA (p110-alpha), p120GAP, GSK3 beta, RelA (p65 NF-kB subunit), PDGF-B, MYH11, K-RAS, PTEN, HIF1A, ERK1/2, c-Abl, Transgelin, Beta-catenin, ACTA2, Inversin, PDGFR-ab, NF-kB, PDGF-R-alpha, AKT(PKB), SP1, PDGF receptor, NF-kB p50/p65, PDK (PDPK1), DDX5, Elk-1, SRF, Phox1 (PRRX1), PDGF-R-beta
**18**	Extracellular matrix-regulated proliferation of airway smooth muscle cells in asthma	1.707E-10	1.393E-08	COL1A1, PI3K cat class IA, PDGF-B, ATF-2, SMAD7, EGFR, ERK1/2, Perlecan, alpha-2/beta-1 integrin, alpha-5/beta-1 integrin, JNK(MAPK8-10), CTGF, Collagen IV, Fibronectin, p38 MAPK, LAMA1, Collagen I, TGF-beta receptor type I, Collagen III

**19**	Ligand-independent activation of Androgen receptor in Prostate Cancer	1.796E-10	1.393E-08	STAT3, Tip60, Shc, PI3K cat class IA, GSK3 beta, B-Raf, K-RAS, EGFR, N-Ras, FGF1, c-Abl, IGF-1 receptor, Beta-catenin, PP2A regulatory, c-Raf-1, NCOA1 (SRC1), SOS, PP2A catalytic, FRS2, ERK2 (MAPK1), AKT(PKB), PDK (PDPK1), NCOA2 (GRIP1/TIF2), DDX5, STAT5B, ERK1 (MAPK3), Frizzled
**20**	E-cadherin signaling and its regulation in gastric cancer	3.260E-10	2.401E-08	Ubiquitin, RhoA, Formin, Shc, GSK3 beta, WNT, SLUG, EGFR, FGF1, Beta-catenin, Alpha-actinin, IQGAP1, HAKAI, HGF receptor (Met), TCF8, Dsh, Actin, Alpha-catenin, Frizzled
**21**	Development_Positive regulation of WNT/Beta-catenin signaling in the nucleus	3.974E-10	2.787E-08	SMYD2, Kindlin-2, CBP, Sirtuin1, GSK3 beta, RUNX, WNT, FOXK2, XIAP, Alpha-1 catenin, USP5, Beta-catenin, BCL9/B9L, APPL, FOXK1, ERK2 (MAPK1), p300, CBP/P300, LRRFIP2, NCOA2 (GRIP1/TIF2), Dsh, Casein kinase I alpha, SOX9, Frizzled, UBR5, JRK, YAP1 (YAp65)
**22**	Oxidative stress_ROS signaling	4.834E-10	3.237E-08	ELAVL1 (HuR), PRKD1, Sirtuin1, GSK3 beta, p70 S6 kinase1, RelA (p65 NF-kB subunit), SREBP1 (nuclear), PKC, PTEN, MEKK1(MAP3K1), IRP1, PKA-cat alpha, HIF1A, ATM, DLC1 (Dynein LC8a), IRP2, ERK1/2, GRP75, c-Abl, SENP1, EGR1, PKA-reg (cAMP-dependent), JNK(MAPK8-10), HIF-prolyl hydroxylase, NF-kB, SAE2, AKT(PKB), Isoform p66 Shc, SP1, NF-kB p50/p65, p300, NRF2, ADAM17, p38 MAPK, PAI1
**23**	Autophagy_Autophagy	7.052E-10	4.498E-08	Sequestosome 1(p62), Tip60, PI3K reg class III (p150), APG16L1, Sirtuin1, GSK3 beta, Raptor, C/EBP zeta, HSC70, RalB, eIF2AK3, PKC-alpha, APG7, SNAP-29, AMBRA1, Beclin 1, CREB1, Sec8, SUR-8, PI3K cat class III (Vps34), Exo84, NBR1, Syntaxin 17, Rubicon, APG12, FIP200
**24**	Cytoskeleton remodeling_Integrin outside-in signaling	7.328E-10	4.498E-08	TRIO, Talin, alpha-11/beta-1 integrin, GSK3 beta, Vinculin, PINCH, ERK1/2, FAK1, alpha-2/beta-1 integrin, Beta-catenin, alpha-5/beta-1 integrin, Alpha-parvin, Alpha-actinin, c-Raf-1, SOS, Filamin A, AKT(PKB), Collagen IV, Fibronectin, ILK, Collagen I, WIRE
**25**	Development_Negative regulation of STK3/4 (Hippo) pathway and positive regulation of YAP/TAZ function	8.362E-10	4.801E-08	RhoA, MLCP (reg), G-protein alpha-12 family, PARD3, PDZ-RhoGEF, Nephrocystin-4, STK3, ASPP1, EGFR, Itch, ASPP2, LARG, ERK1/2, LATS2, TAZ, JNK(MAPK8-10), PAR1, RhoGAP5, PJA2, PDK (PDPK1), Actin, ERK1 (MAPK3), ILK, YAP1 (YAp65), ZO-2
**26**	Development_Stimulation of differentiation of mouse embryonic fibroblasts into	8.475E-10	4.801E-08	SREBP1 precursor, Shc, CBP, EGR2 (Krox20), PI3K cat class IA (p110-alpha), HIVEP2, Lysyl oxidase, BMP receptor 2, ATF-2, SHP-2, Adenylate cyclase, XIAP, ATF-1, ERK1/2, CREB1, IGF-1 receptor, AKT2, p90RSK2(RPS6KA3), PKA-reg (cAMP-dependent), c-Raf-1, SOS, ERK2 (MAPK1), PDK (PDPK1), p38 MAPK, ERK1 (MAPK3), Elk-1, PKA-cat (cAMP-dependent)

	adipocytes by extracellular factors			
**27**	Signal transduction_Angiotensin II/ AGTR1 signaling via p38, ERK and PI3K	8.900E-10	4.856E-08	ELAVL1 (HuR), Shc, p70 S6 kinases, PI3K cat class IA, RECK, PDGF-B, NUR77, EGFR, MAP2K5 (MEK5), ERK1/2, SP3, CREB1, IGF-1 receptor, EGR1, G-protein beta/gamma, MEF2A, Fyn, c-Raf-1, HDAC4, SOS, AKT(PKB), SP1, OSF-2, PDK (PDPK1), ADAM17, p38 MAPK, Elk-1, PAI1, PKA-cat (cAMP-dependent), p90Rsk, CaMK II delta, PDGF-R-beta, ATP7A
**28**	Cytoskeleton remodeling_Regulation of actin cytoskeleton organization by the kinase effectors of Rho GTPases	9.678E-10	5.091E-08	Talin, RhoA, MLCP (reg), ROCK, MRCKalpha, PAK, Cdc42 subfamily, RhoJ, Vinculin, MSN (moesin), ERM proteins, Rhov, Spectrin, PRK1, RhoA-related, Caldesmon, Alpha-actinin, Filamin A, Alpha adducin, PIP5KI, MyHC, MLCK, Cofilin, MRCK
**29**	Neurophysiological process_Dynein-dynactin motor complex in axonal transport in neurons	1.088E-09	5.526E-08	Ubiquitin, Importin (karyopherin)-beta, Rab-5A, Centractins, DYNLL, ORP1, Dynein 1, cytoplasmic, heavy chain, Kinesin heavy chain, ERK1/2, DCTN1(p150Glued), BPAG1, PRNP, AKT(PKB), Piccolo, Kinesin light chain, PAFAH alpha (LIS1), Dynein 1, cytoplasmic, intermediate chains, DYNC1I1, MAPRPE1(EB1), DYI2, NUDEL, SPTBN2, Tubulin (in microtubules)
**30**	Development_TGF-beta-dependent induction of EMT via RhoA, PI3K and ILK	1.224E-09	5.814E-08	RhoA, PI3K cat class IA, GSK3 beta, RelA (p65 NF-kB subunit), PINCH, SMURF1, SLUG, ZO-1, ROCK1, MKL2, Beta-catenin, Caldesmon, ACTA2, AKT(PKB), PDK (PDPK1), Fibronectin, Actin, SRF, ILK, Cofilin, TGF-beta receptor type I
**31**	IGF-1 signaling in multiple myeloma	1.224E-09	5.814E-08	RhoA, Shc, c-IAP2, PRKD1, PI3K cat class IA, FOXO4, p70 S6 kinase1, PTEN, HIF1A, XIAP, ERK1/2, GSK3 alpha/beta, IGF-1 receptor, c-FLIP(Long), c-Raf-1, SOS, NF-kB, ERK2 (MAPK1), AKT(PKB), PDK (PDPK1), ERK1 (MAPK3)
**32**	Mechanisms of cell adhesion-mediated-drug resistance (CAM-DR) in multiple myeloma	1.668E-09	7.676E-08	STAT3, TOP2 beta, Talin, RhoA, ITGB1, c-IAP2, ROCK, gp130, GGTase-I, ICAM1, Vinculin, WNT3, Cyclin A, alpha-5/beta-1 integrin, HSP70, Fibronectin, Dsh, Frizzled

**33**	Regulation and signaling of HGF receptor (Met) and MSP receptor (RON) in lung cancer	1.747E-09	7.799E-08	STAT3, Shc, PI3K cat class IA, GSK3 beta, p70 S6 kinase1, Rb protein, NCK1, K-RAS, c-Cbl, MEKK1(MAP3K1), PKC-alpha, HIF1A, ATF-2, ERK1/2, FAK1, CREB1, CrkL, Beta-catenin, Delta-catenin, SOS, Alpha adducin, AKT(PKB), SP1, Thrombospondin 1, PDK (PDPK1), HGF receptor (Met), p90Rsk
**34**	TGF-beta-induced fibroblast/ myofibroblast migration and extracellular matrix production in asthmatic airways	2.209E-09	9.570E-08	COL1A1, ITGA1, ITGB1, PI3K cat class IA, COL4A1, TIMP3, ITGA2, ERK1/2, JNK(MAPK8-10), COL1A2, ABCC5, AKT(PKB), COL5A1, SP1, Collagen IV, TIMP2, ITGA5, Fibronectin, p38 MAPK, PAI1, Collagen I, TGF-beta receptor type I, Collagen III, Thrombospondin 2
**35**	G-protein signaling_Rac1 activation	2.479E-09	1.043E-07	TRIO, GEFT, PI3K cat class IA, PAK, EPS8, Ephrin-A1, K-RAS, AF-6, EGFR, CDEP, Tiam2, DOCK1, KIDINS220, CaMK I, G-protein beta/gamma, Rho GTPase, DOCK7, DOCK6, CRK, SOS, ALS2, p200RhoGAP, FARP2, SHANK, PKA-cat (cAMP-dependent), E3b1(ABI-1), DOCK4
**36**	MAPK-mediated proliferation of normal and asthmatic smooth muscle cells	2.578E-09	1.055E-07	Shc, p70 S6 kinases, Rb protein, EDNRB, PDGF-B, K-RAS, PKC-alpha, PLAT (TPA), EGFR, N-Ras, ERK1/2, CREB1, G-protein beta/gamma, c-Raf-1, SOS, PDGF-R-alpha, PDGF receptor, Elk-1, PAI1, p90Rsk, EDNRA, TGF-beta receptor type I, PDGF-R-beta
**37**	Cell adhesion_PLAU signaling	2.706E-09	1.077E-07	STAT3, FPRL1, RhoA, Shc, c-IAP2, ROCK, Nucleolin, gp130, PI3K cat class IA, LAMC2, c-IAP1, SHP-2, EGFR, XIAP, ERK1/2, FAK1, alpha-5/beta-1 integrin, c-Raf-1, SOS, NF-kB, AKT(PKB), MYLK1, STAT5B, MLCK, PDGF-R-beta
**38**	Cell adhesion_Tight junctions	3.342E-09	1.295E-07	RhoA, APXL, ROCK, JAM3, PARD3, MUPP1, PDZ-RhoGEF, EPB41, MPP5, Tubulin alpha, ARP3, PKC-lambda/iota, AF-6, N-WASP, ZO-1, ACTR3, CGNL1, Actin, ZO-2, Tubulin (in microtubules)
**39**	Development_Positive regulation of STK3/4 (Hippo) pathway and negative regulation of YAP/TAZ function	4.467E-09	1.615E-07	RhoA, Willin, MPP5, Cullin 2, PKA-cat alpha, STK3, AMPK beta subunit, Adenylate cyclase, Itch, Alpha-1 catenin, 14-3-3, MARK4, LATS2, Beta-catenin, Skp2/TrCP/FBXW, TAZ, PKA-reg (cAMP-dependent), MARKK, c-Raf-1, PEZ, Actin, PKA-cat (cAMP-dependent), Alpha-catenin, AMOTL2, YAP1 (YAp65), ZO-2
**40**	Signal transduction_CXCR4 signaling via MAPKs cascades	4.506E-09	1.615E-07	Plectin 1, Ubiquitin, RhoA, ROCK, PAK, RelA (p65 NF-kB subunit), PDGF-B, NF-kB1 (p50), K-RAS, MEKK1(MAP3K1), N-Ras, ERK1/2, CREB1, EGR1, G-protein beta/gamma, p90RSK2(RPS6KA3), JNK(MAPK8-10), c-Raf-1, CTGF, p38 MAPK, Elk-1, G-protein alpha-13

**41**	Cell cycle_Influence of Ras and Rho proteins on G1/S Transition	4.506E-09	1.615E-07	STAT3, RhoA, MLCP (reg), PI3K cat class IA, GSK3 beta, p70 S6 kinase1, RelA (p65 NF-kB subunit), Rb protein, ROCK2, GGTase-I, Cyclin A2, ATF-2, DIA1, ERK1/2, FAK1, RGL2, alpha-5/beta-1 integrin, c-Raf-1, AKT(PKB), NF-kB p50/p65, PDK (PDPK1), MLCK
**42**	Deficient alpha-MSH signaling in melanoma	4.605E-09	1.615E-07	RhoA, PI3K cat class IA, GSK3 beta, Rb protein, B-Raf, PKA-cat alpha, N-Ras, ROCK1, ERK1/2, CREB1, PKA-reg (cAMP-dependent), c-Raf-1, AKT(PKB), PDK (PDPK1), p38 MAPK, PKA-cat (cAMP-dependent), SOX9
**43**	G-protein signaling_RhoA activation	5.286E-09	1.811E-07	TRIO, RhoA, GEFT, p190-RhoGEF, G-protein alpha-12 family, MUPP1, PDZ-RhoGEF, MPP5, Cyclin B, PKC-alpha, SMURF1, Tensin 2, EGFR, ZO-1, Synaptopodin, XIAP, LARG, p190RhoGAP, FAK1, IGF-1 receptor, G-protein beta/gamma, RHG7, Ephrin-A receptors, PAR1, Collagen IV, Ephrin-A, Reticulon 4, Fibronectin, PTPN12
**44**	Development_NOTCH signaling activation	7.048E-09	2.359E-07	NOTCH2 (2ICD), CBP, CARM1, PHF8, FRYL, SMRT, MAML1, Itch, BRG1, NOTCH2(NEXT), KDELC2, PI3K class II (CII-alpha), Beta-catenin, N-CoR, KDELC1, MAML2, MAGP2, ZFP64, AAK1, p300, Furin, NOTCH2 receptor, ADAM10, Jagged1, DDX5, ADAM17, BAF60c, Thrombospondin 2
**45**	WNT signaling in proliferative-type melanoma cells	9.065E-09	2.805E-07	GSK3 beta, B-Raf, DVL-3, NUR77, DKK3, WNT, APC protein, WNT2, ERK1/2, LRP6, Beta-catenin, NURR1, Fibronectin, Dsh, Casein kinase I alpha, SOX9, Frizzled, DACT1
**46**	Apoptosis and survival_BAD phosphorylation	9.127E-09	2.805E-07	Shc, PI3K cat class IA, p70 S6 kinase1, Calcineurin A (catalytic), EGFR, ERK1/2, Beclin 1, 14-3-3, IGF-1 receptor, G-protein beta/gamma, PKA-reg (cAMP-dependent), c-Raf-1, SOS, PP2C, PP2A catalytic, AKT(PKB), PDK (PDPK1), PKA-cat (cAMP-dependent), p90Rsk
**47**	Signal transduction_Angiotensin II/ AGTR1 signaling via RhoA and JNK	9.331E-09	2.805E-07	COL1A1, RhoA, MLCP (reg), GEFT, ROCK, STIM1, LBC, G-protein alpha-12 family, RECK, PDZ-RhoGEF, NCK1, ROCK2, MEKK1(MAP3K1), Vinculin, ATF-2, G-protein alpha-12, LARG, ERK1/2, FAK1, G-protein beta/gamma, CRK, JNK(MAPK8-10), CTGF, PAI1, SRF, MLCK, Collagen III
**48**	Signal transduction_Angiotensin II/AGTR1 signaling via Notch, Beta-catenin and NF-kB pathways	9.331E-09	2.805E-07	COL1A1, RhoA, CBP, PRKD1, GSK3 beta, RelA (p65 NF-kB subunit), PKC, TRAF6, ROCK1, ERK1/2, IGF-1 receptor, Beta-catenin, ACTA2, NCOA1 (SRC1), NF-kB, CTGF, ERK2 (MAPK1), AKT(PKB), NF-kB p50/p65, p300, PDK (PDPK1), Fibronectin, ADAM10, ADAM17, p38 MAPK, PKA-cat (cAMP-dependent), YAP1 (YAp65)

**49**	G-protein signaling_RhoA inhibition	9.331E-09	2.805E-07	RhoA, RhoGAP1, Rap1, ABL2, PRKD1, p120GAP, Protein kinase G1, MARK2, Cyclin B, SMURF1, p190RhoGAP, FAK1, 14-3-3 gamma, RHG7, Fyn, PKA-reg (cAMP-dependent), RhoGAP5, PRNP, Guanylate Cyclase 1, soluble, ERK2 (MAPK1), ARAP3, ERK1 (MAPK3), PKA-cat (cAMP-dependent), SLK, Alpha-catenin, MYO9A, Tubulin (in microtubules)
**50**	Development_Regulation of epithelial-to-mesenchymal transition (EMT)	1.014E-08	2.987E-07	Arkadia, RelA (p65 NF-kB subunit), PDGF-B, ATF-2, WNT, SLUG, EGFR, ZO-1, IL-1RI, CREB1, Caldesmon, ACTA2, PDGF-R-alpha, SP1, HGF receptor (Met), Fibronectin, Jagged1, TCF8, PAI1, SRF, Frizzled, EDNRA, TGF-beta receptor type I, PDGF-R-beta

Table S_3: Pathway analysis of gene co-expressed ITGB3 (GDC) from the MetaCore database (with p-value< 0.05 set as the cutoff value).

S.No.	Maps	pValue	Min FDR	Network Objects from Active Data
**1**	Cell adhesion_ECM remodeling	6.087E-19	9.081E-16	Laminin 5, MMP-13, TIMP3, SERPINE2, PLAT (TPA), MSN (moesin), MMP-1, MMP-16, Kallikrein 2, MMP-14, MMP-2, Versican, MMP-12, HB-EGF, IGF-1 receptor, Matrilysin (MMP-7), PLAUR (uPAR), Actin cytoskeletal, Collagen II, Collagen IV, TIMP2, Fibronectin, Kallikrein 1, Nidogen, Osteonectin, Stromelysin-1, LAMA4, MMP-9, PAI1, PLAU (UPA), Collagen I, TIMP1, Kallikrein 3 (PSA), Collagen III
**2**	TGF-beta-induced fibroblast/ myofibroblast migration and extracellular matrix production in asthmatic airways	2.659E-18	1.984E-15	COL1A1, ITGA1, Biglycan, TGF-beta 2, TGF-beta 1, PI3K cat class IA, COL4A1, MMP-13, TIMP3, ITGA2, Decorin, Endothelin-1, ITGA3, TGF-beta 3, MMP-1, HAS2, TGF-beta receptor type II, ERK1/2, MMP-2, JNK(MAPK8-10), COL1A2, AKT(PKB), COL5A1, Collagen IV, TIMP2, ITGA5, Fibronectin, Stromelysin-1, MMP-9, PAI1, Collagen I, TIMP1, TGF-beta receptor type I, Collagen III, Thrombospondin 2
**3**	IL-1 beta- and Endothelin-1-induced fibroblast/ myofibroblast migration and extracellular matrix production in asthmatic airways	5.552E-18	2.761E-15	COL1A1, IL-1 beta, IL-1 alpha, COL4A1, EDNRB, PDGF-B, TIMP3, Decorin, Endothelin-1, MMP-1, HAS2, ERK1/2, MMP-2, Versican, IL-1RI, COL1A2, NF-kB, CTGF, PDGF-R-alpha, Thrombospondin 1, Fibronectin, Stromelysin-1, PAI1, EDNRA, Collagen I, TIMP1, PDGF-R-beta, Collagen III
**4**	Development_Regulation of epithelial-to-mesenchymal transition (EMT)	4.475E-17	1.669E-14	HGF, IL-1 beta, HEY1, VE-cadherin, TGF-beta 2, TGF-beta 1, PDGF-B, Endothelin-1, TGF-beta 3, WNT, TGF-beta receptor type II, SLUG, SIP1 (ZFHX1B), MMP-2, IL-1RI, CREB1, NOTCH4 receptor, Caldesmon, ACTA2, FGF2, SNAIL1, PDGF-R-alpha, TWIST1, TNF-R1, HGF receptor (Met), Fibronectin, TCF8, DLL4, MMP-9, PAI1, SRF, Frizzled, EDNRA, TGF-beta receptor type I, PDGF-R-beta
**5**	Chemotaxis_Lysophosphatidic acid signaling via GPCRs	2.833E-16	8.455E-14	RhoA, MLCP (reg), LPAR4, ROCK, PRKD1, G-protein alpha-12 family, Caspase-7, PAK, AKT1, p70 S6 kinase1, PKC, PLC-beta, Vinculin, Tcf(Lef), HAS2, LPAR6, G-protein alpha-i family, F-Actin cytoskeleton, Caspase-3, PLC-delta 1, ROCK1, FasR(CD95), ERK1/2, Cyr61, PRK1, FAK1, HB-EGF, CREB1, Beta-catenin, G-protein beta/gamma, Rho GTPase, CDC42, Actin cytoskeletal, IL13RA2, JNK(MAPK8-10), c-Raf-1, Caspase-9, CTGF, AKT(PKB), G-protein alpha-q/11, LIMK, ADAM17,

				PLC-epsilon, Elk-1, SRF, Paxillin, IP3 receptor, mTOR, YAP1 (YAp65), G-protein gamma 12, PREX1
**6**	Stellate cells activation and liver fibrosis	1.804E-15	4.486E-13	COL1A1, IL-1 beta, Biglycan, TGF-beta 1, PI3K cat class IA, PDGF-B, PTCH1, MyD88, Tcf(Lef), TGF-beta receptor type II, RIPK1, MMP-2, IL-1RI, IKK-gamma, Beta-catenin, ACTA2, IRAK1/2, c-Raf-1, SOS, COL1A2, GLI-1, PDGF-R-alpha, ERK2 (MAPK1), AKT(PKB), PDGF receptor, TNF-R1, SARA, Dsh, ERK1 (MAPK3), Elk-1, Frizzled, TLR2, TIMP1, TGF-beta receptor type I, PDGF-R-beta
**7**	Signal transduction_Endothelin-1/ EDNRA signaling	7.356E-15	1.408E-12	MLCP (cat), RhoA, MLCP (reg), Rap1, ROCK, PRKD1, PI3K cat class IA, BCAR3, Calmodulin, PKC, PLC-beta, Endothelin-1, G-protein alpha-12, cPLA2, G-protein alpha-i family, Adenylate cyclase, SLC9A1, ERK1/2, MEF2C, FAK1, Plexin A4, eEF2K, CREB1, IP3R1, ACTA2, G-protein beta/gamma, CDC42, JNK(MAPK8-10), TRPC6, LIPS, CTGF, AKT(PKB), G-protein alpha-q/11, p300, FKBP1B, FARP2, EDNRA, IP3 receptor
**8**	Development_NOTCH/ TGF-beta crosstalk in EMT	7.549E-15	1.408E-12	COL1A1, NOTCH4 (ICD4), HEY1, VE-cadherin, TGF-beta 2, TGF-beta 1, TIE2, HEYL, TGF-beta receptor type II, SLUG, TCF7L2 (TCF4), SIP1 (ZFHX1B), Transgelin, Beta-catenin, NOTCH4 receptor, ACTA2, HEY2, HMGA2, Calponin-1, SNAIL1, COL1A2, LSD1, NRF2, TCF8, DLL4, MMP-9, TGF-beta receptor type I
**9**	Signal transduction_S1P2 receptor activation signaling	2.255E-14	3.738E-12	STAT3, MLCP (cat), COX-2 (PTGS2), RhoA, MLCP (reg), JAK2, ROCK, G-protein alpha-12 family, PI3K cat class IA, PDGF-B, BMP receptor 2, RUNX2, PLC-beta, MYH11, G-protein alpha-12, G-protein alpha-i family, MRLC, ROCK1, ERK1/2, FAK1, HB-EGF, CREB1, SMAD9 (SMAD8), Transgelin, Beta-catenin, ACTA2, G-protein beta/gamma, Actin cytoskeletal, G-protein alpha-q, SMAD1, JNK(MAPK8-10), c-Raf-1, NF-kB, TRPC6, LIMK2, AKT(PKB), PPAR-gamma, G-protein alpha-i2, SRF, Paxillin, IP3 receptor, YAP1 (YAp65), G-protein alpha-13

**10**	Signal transduction_ESR1 (membrane) and ESR2 (membrane) signaling	2.619E-14	3.907E-12	RhoA, p120GAP, PI3K cat class IA, Protein kinase G1, Profilin, Calmodulin, PKC, PLC-beta, ROCK2, MSN (moesin), IRS-1, Tcf(Lef), G-protein alpha-i family, Adenylate cyclase, ERK1/2, eNOS, MMP-2, FAK1, HB-EGF, CACNA1C, CREB1, IGF-1 receptor, Beta-catenin, G-protein beta/gamma, CDC42, G-protein alpha-q, PKA-reg (cAMP-dependent), c-Raf-1, G-protein alpha-i3, SOS, Striatin, AKT(PKB), Caveolin-1, WASF1(WAVE1), FASN, G-protein beta1/gamma2, Elk-1, MMP-9, G-protein alpha-i2, SRF, p90Rsk, G-protein beta-1, IP3 receptor, G-protein alpha-13, PR (nuclear)
**11**	Signal transduction_Non-canonical WNT5A signaling	1.175E-13	1.593E-11	MLCP (cat), RhoA, MLCP (reg), ROCK, MCAM, TIE2, WNT5A, ROR2, FZD2, AKT1, Calcineurin A (catalytic), Calmodulin, CCDC88C, PLC-beta, FZD6, PKC-lambda/iota, MMP-1, G-protein alpha-i family, TCF7L2 (TCF4), ERK1/2, Protein kinase G, Beta-catenin, FZD3, G-protein beta/gamma, CDC42, G-protein alpha-q, JNK(MAPK8-10), FZD9, Filamin A, LIMK2, TAB2, FZD7, Elk-1, NF-AT, Paxillin, IP3 receptor
**12**	Role of endothelial and immune cells in systemic sclerosis	3.282E-13	4.080E-11	COL1A1, Cathepsin L, ST2(L), TGF-beta 1, GCP2, Protein C receptor (endothelial), Endothelin-1, MyD88, MMP-1, TGF-beta receptor type II, TLR4, Neuropilin-1, FasR(CD95), Cyr61, ENA-78, MMP-12, EPAS1, Endoglin, ACTA2, PLAUR (uPAR), SMAD1, TLR8, COL1A2, Caveolin-1, Fibronectin, Cathepsin V, EDNRA, TIMP1, TGF-beta receptor type I, YAP1 (YAp65)
**13**	Cell adhesion_PLAU signaling	3.818E-13	4.381E-11	STAT3, FPRL1, RhoA, c-IAP2, ROCK, PI3K cat class IA, LAMC2, c-IAP1, SHP-2, G-protein alpha-i family, XIAP, F-Actin cytoskeleton, MRLC, ERK1/2, FAK1, PLAUR (uPAR), CDC42, c-Raf-1, VEGFR-2, G-protein alpha-i3, SOS, NF-kB, AKT(PKB), Caveolin-1, MYLK1, sUPAR, PLAU (UPA), MLCK, FPR, Paxillin, PDGF-R-beta
**14**	Induction of fibrosis in systemic sclerosis	4.450E-13	4.712E-11	COL1A1, STAT3, JAK2, TGF-beta 1, IL-1 alpha, IL13RA1, Endothelin-1, MMP-1, WNT, TGF-beta receptor type II, TLR4, ERK1/2, IL-1RI, IL-6, Endoglin, Beta-catenin, ACTA2, SMAD1, IL13RA2, JNK(MAPK8-10), c-Raf-1, COL1A2, CTGF, LAP beta 1, COL5A1, PDGF receptor, p300, Thrombospondin 1, Fibronectin, Stromelysin-1, Cathepsin V, PPAR-gamma, Frizzled, EDNRA, TGF-beta receptor type I, Collagen III

**15**	Cytoskeleton remodeling_Regulation of actin cytoskeleton organization by the kinase effectors of Rho GTPases	4.737E-13	4.712E-11	MLCP (cat), BETA-PIX, Talin, RhoA, MLCP (reg), ROCK, MRCKalpha, PAK, Cdc42 subfamily, RhoJ, Vinculin, MSN (moesin), ERM proteins, F-Actin cytoskeleton, MRLC, SLC9A1, PRK1, RhoA-related, Caldesmon, CDC42, Actin cytoskeletal, Alpha-actinin, Filamin A, CPI-17, MyHC, LIMK, MLCK, Paxillin, MRCK
**16**	Role of TGF-beta 1 in fibrosis development after myocardial infarction	5.880E-13	5.483E-11	COL1A1, Biglycan, TGF-beta 1, EDNRB, Endothelin-1, MMP-1, TGF-beta receptor type II, MMP-2, ACTA2, ACE1, COL1A2, CTGF, TIMP2, Thrombospondin 1, Fibronectin, Prolyl endopeptidase, MMP-9, PAI1, EDNRA, Collagen I, TIMP1, TGF-beta receptor type I, Collagen III
**17**	Transcription_HIF-1 targets	8.702E-13	7.637E-11	NIX, TGF-beta 2, TGF-beta 1, GLUT1, PDGF-B, Lysyl oxidase, ROR-alpha, Endothelin-1, CITED2, PLGF, TGF-beta 3, HXK2, HIF1A, HXK1, P4HA2, SLC9A1, LOXL2, MMP-2, Endoglin, PLAUR (uPAR), VEGFR-1, P4HA1, FGF2, GLUT3, ENO1, Angiopoietin 2, CTGF, ABCG2, Alpha-1B adrenergic receptor, MGF, DEC1 (Stra13), Thrombospondin 1, HGF receptor (Met), MSH6, MMP-9, PAI1, LRP1, IBP3
**18**	Stromal-epithelial interaction in Prostate Cancer	1.077E-12	8.931E-11	HGF, Keratin 17, TGF-beta 2, TGF-beta 1, PDGF-B, TGF-beta 3, TGF-beta receptor type II, MMP-2, Versican, IL-6, IGF-1 receptor, ACTA2, FGF2, SNAIL1, PDGF-R-alpha, HIC5, TIMP2, HGF receptor (Met), Fibronectin, MMP-9, Collagen I, TIMP1, TGF-beta receptor type I, PDGF-R-beta
**19**	MAPK-mediated proliferation of normal and asthmatic smooth muscle cells	1.199E-12	9.416E-11	TGF-beta 1, p70 S6 kinases, Amphiregulin, Rb protein, EDNRB, PDGF-B, PLC-beta, K-RAS, Endothelin-1, PLAT (TPA), N-Ras, G-protein alpha-i family, ERK1/2, HB-EGF, CREB1, G-protein beta/gamma, FGF2, c-Raf-1, SOS, PDGF-R-alpha, PDGF receptor, Elk-1, PAI1, p90Rsk, EDNRA, IP3 receptor, TGF-beta receptor type I, PDGF-R-beta
**20**	Development_Hedgehog signaling	3.100E-12	2.312E-10	CARD7, RhoA, CDON, ROCK, BOC, PI3K cat class IA, Calmodulin, RUNX2, GLI-3, PLC-beta, NOX4, KIF7, PTCH1, FHL2, PTCH2, HIF1A, Bone sialoprotein, G-protein alpha-i family, Adenylate cyclase, Caspase-3, Liprin-alpha1, G-protein beta/gamma, PKA-reg (cAMP-dependent), Collagen X, PRMT5, PA24A, GLI-1, DLG5(P-dlg), Ihh, Caspase-9, AKT(PKB), Dynein 1, cytoplasmic, intermediate chains, FoxD1, SUFU, Casein kinase I alpha, GLI-2, DOCK4

**21**	Signal transduction_PDGF signaling via PI3K/AKT and NFkB pathways	4.504E-12	3.200E-10	PI3K cat class IA (p110-alpha), p120GAP, Myocardin, PDGF-B, Calmodulin, MYH11, K-RAS, PTEN, HXK2, HIF1A, ETS1, ERK1/2, MMP-2, Transgelin, Beta-catenin, ACTA2, SGK1, Inversin, Thrombomodulin, PDGFR-ab, NF-kB, PDGF-R-alpha, AKT(PKB), SOS1, PDGF receptor, Elk-1, MMP-9, SRF, Phox1 (PRRX1), mTOR, PDGF-R-beta
**22**	Role of alpha-V/ beta-6 integrin in colorectal cancer	5.047E-12	3.423E-10	TGF-beta 1, PKC, ITGAV, LTBP1, ETS1, Caspase-3, ERK1/2, MMP-2, alpha-V/beta-6 integrin, Caspase-9, ERK2 (MAPK1), Collagen IV, Fibronectin, ITGB6, MMP-9, PLAU (UPA), Collagen I
**23**	Role of stellate cells in progression of pancreatic cancer	1.025E-11	6.651E-10	COL1A1, TGF-beta 1, PI3K cat class IA, MMP-13, PDGF-B, TGF-beta receptor type II, ERK1/2, MMP-2, FAK1, HB-EGF, IL-6, ACTA2, FGF2, c-Raf-1, SOS, COL1A2, CTGF, PDGF-R-alpha, AKT(PKB), PDGF receptor, OSF-2, Fibronectin, Stromelysin-1, Collagen I, TIMP1, TGF-beta receptor type I, PDGF-R-beta, Collagen III
**24**	HGF signaling in colorectal cancer	1.095E-11	6.808E-10	HGF, COX-2 (PTGS2), Laminin 5, PI3K cat class IA, AKT1, p70 S6 kinase1, LAMC2, PTEN, SHP-2, XIAP, TCF7L2 (TCF4), ERK1/2, MMP-2, FAK1, Beta-catenin, PLAUR (uPAR), Actin cytoskeletal, JNK(MAPK8-10), c-Raf-1, SOS, AKT(PKB), LAMB3, TIMP2, Thrombospondin 1, HGF receptor (Met), MMP-9, LAMA3 (Epiligrin), TIMP1, mTOR
**25**	Signal transduction_Non-neuronal ACM1, ACM3 and ACM5 signaling	2.646E-11	1.474E-09	MLCP (cat), RhoA, MLCP (reg), ROCK, p70 S6 kinases, PI3K cat class IA, Rb protein, Adenylate cyclase type VIII, Calmodulin, ITGA2, PKC, ITGA3, G-protein alpha-12, Adenylate cyclase, MRLC, ERK1/2, MEF2C, Protein kinase G, G-protein alpha-11, G-protein alpha-q, PKA-reg (cAMP-dependent), c-Raf-1, CPI-17, AKT(PKB), G-protein alpha-q/11, PLC-epsilon, RAP-2B, SRF, MLCK, mTOR, Adenylate cyclase type III
**26**	Signal transduction_Angiotensin II/ AGTR1 signaling via p38, ERK and PI3K	2.715E-11	1.474E-09	COX-2 (PTGS2), JAK2, p70 S6 kinases, PI3K cat class IA, PDGF-B, Calmodulin, WISP1, ETS1, cPLA2, Cal-DAG-GEFII, TLR4, eIF4E, ERK1/2, MEF2C, MMP-14, SP3, MMP-2, HB-EGF, CREB1, IL-6, IGF-1 receptor, G-protein beta/gamma, MEF2A, Fyn, G-protein alpha-q, c-Raf-1, SOS, AKT(PKB), MNK1, OSF-2, ADAM17, Elk-1, PAI1, p90Rsk, CaMK II delta, PDGF-R-beta, ATP7A
**27**	Glucocorticoid-induced elevation of intraocular pressure as glaucoma risk factor	2.744E-11	1.474E-09	TRIO, COX-2 (PTGS2), RhoA, Laminin 5, ROCK, PI3K cat class IA, WNT5A, ROR2, COL4A1, PLAT (TPA), MMP-1, Filamin B (TABP), WNT2, MMP-2, FAK1, SENP1, CDC42, Actin cytoskeletal, Alpha-actinin,

				Filamin A, LAMB3, Collagen IV, Thrombospondin 1, HGF receptor (Met), Fibronectin, Stromelysin-1, PAI1, MLCK
**28**	TGF-beta signaling via SMADs in breast cancer	2.766E-11	1.474E-09	TGF-beta 2, Cyclin A1, TGF-beta 1, MMP-13, RUNX2, NOX4, CITED2, TGF-beta 3, ETS1, TGF-beta receptor type II, SLUG, TBX2, MMP-14, HMGA2, SNAIL1, Ihh, CTGF, TWIST1, FOXQ1 (HFH1), MMP-9, PAI1, IL-11, TGF-beta receptor type I, GLI-2
**29**	Development_Regulation of cytoskeleton proteins in oligodendrocyte differentiation and myelination	4.243E-11	2.143E-09	MLCP (cat), Tubulin beta, RhoA, MLCP (reg), CNTN1 (F3), Tubulin alpha, MAP4, ROCK2, Netrin-1, MRLC, L1CAM, FAK1, chTOG, Dcc, CDC42, Actin cytoskeletal, Fyn, CDK5R2 (p39), HDAC6, KLHL2, PDGF-R-alpha, PDGF receptor, WASF1(WAVE1), Gelsolin, WASF2, Paxillin, Tubulin (in microtubules)
**30**	Resolution of inflammation in healing myocardial infarction	4.309E-11	2.143E-09	STAT3, HGF, JAK2, TGF-beta 1, TIE2, MMP-1, ERK1/2, MMP-2, Angiopoietin 1, FGF2, VEGFR-2, NF-kB, AKT(PKB), MGF, HGF receptor (Met), G-CSF, MMP-9
**31**	Tumor-stroma interactions in pancreatic cancer	4.728E-11	2.276E-09	HGF, Galectin-3, TGF-beta 1, PDGF-B, PTCH1, HIF1A, MMP-1, MMP-2, FGF2, COL1A2, GLI-1, OSF-2, HGF receptor (Met), Fibronectin, SUFU, Collagen I, PDGF-R-beta, Thrombospondin 2
**32**	Stimulation of TGF-beta signaling in lung cancer	4.931E-11	2.299E-09	SCUBE3, COX-2 (PTGS2), IL-1 beta, TGF-beta 2, TGF-beta 1, PI3K cat class IA (p110-alpha), Vinculin, TGF-beta 3, TGF-beta receptor type II, SLUG, Ski, IRAKM, MMP-2, ACTA2, TGF-beta, Fyn, SNAIL1, AKT(PKB), MMP-28, Fibronectin, MMP-9, PAI1, TGF-beta receptor type I, Tropomyosin-2
**33**	Th2 cytokine- and TNF-alpha-induced profibrotic response in asthmatic airway fibroblasts/ myofibroblasts	5.843E-11	2.642E-09	COL1A1, COX-2 (PTGS2), IL-1 beta, Biglycan, TGF-beta 1, Decorin, FAN, MMP-1, HAS2, ETS1, ERK1/2, MMP-2, ACTA2, IL13RA2, JNK(MAPK8-10), c-Raf-1, COL1A2, NF-kB, PDGF-R-alpha, AKT(PKB), TNF-R1, MMP-9, Collagen I, TIMP1, Collagen III
**34**	Signal transduction_S1P2 receptor inhibitory signaling	6.501E-11	2.774E-09	MLCP (cat), COX-2 (PTGS2), RhoA, MLCP (reg), VE-cadherin, ROCK, G-protein alpha-12 family, PLC-beta, PTEN, G-protein alpha-12, cPLA2, G-protein alpha-i family, Adenylate cyclase, MRLC, ROCK1, ERK1/2, FAK1, G-protein beta/gamma, G-protein alpha-q, PKA-reg (cAMP-dependent), Alpha-actinin, c-Raf-1, AKT(PKB), G-protein alpha-i2, IP3 receptor, G-protein alpha-13

**35**	Neurophysiological process_Melatonin signaling in the nervous system	6.508E-11	2.774E-09	RhoA, ROCK, GLUT1, PI3K cat class IA, MUPP1, Calmodulin, PKC, PLC-beta, ROR-alpha, Kir3.2, G-protein alpha-i family, Adenylate cyclase, ERK1/2, CREB1, Beta-catenin, G-protein beta/gamma, PKA-reg (cAMP-dependent), CRMP2, c-Raf-1, G-protein alpha-i3, NR2F1, NF-kB, AKT(PKB), G-protein alpha-q/11, NRF2, NQO1, BDNF, G-protein alpha-i2, p90Rsk, IP3 receptor
**36**	MAPK-independent proliferation of normal and asthmatic smooth muscle cells	6.975E-11	2.891E-09	STAT3, Histamine H1 receptor, JAK2, TGF-beta 1, PI3K cat class IA, p70 S6 kinase1, Rb protein, EDNRB, PDGF-B, Endothelin-1, PLAT (TPA), G-protein alpha-i family, HB-EGF, G-protein beta/gamma, FGF2, NF-kB, PDGF-R-alpha, AKT(PKB), G-protein alpha-q/11, Tryptase, PDGF receptor, TBXA2R, CysLT1 receptor, EDNRA, mTOR, TGF-beta receptor type I, PDGF-R-beta, IBP3
**37**	Signal transduction_HTR2A signaling outside the nervous system	1.475E-10	5.801E-09	STAT3, MLCP (cat), COX-2 (PTGS2), RhoA, MLCP (reg), JAK2, TGF-beta 1, ROCK, PI3K cat class IA, p70 S6 kinase1, Rb protein, Calmodulin, PKC, PLC-beta, IRS-1, G-protein alpha-i family, Adenylate cyclase, SLC9A1, ERK1/2, HTR2A, HB-EGF, KV1.5, c-Raf-1, AKT(PKB), G-protein alpha-q/11, Caveolin-1, HB-EGF(mature), ADAM17, mTOR, CaMK II delta
**38**	Angiogenesis in hepatocellular carcinoma (HCC)	1.478E-10	5.801E-09	Ephrin-B1, STAT3, COX-2 (PTGS2), TGF-beta 1, TIE2, Heparanase 1, NF-kB1 (p50), HIF1A, Ephrin-B, TGF-beta receptor type II, Presenilin 1, MMP-2, Presenilin, Angiopoietin 1, IGF-1 receptor, VEGFR-1, FGF2, VEGFR-2, Angiopoietin 2, NF-kB, PGES2, MMP-9, TIE, TGF-beta receptor type I
**39**	Development_The role of GDNF ligand family/ RET receptor in cell survival, growth and proliferation	1.791E-10	6.851E-09	STAT3, RhoA, RAP-1A, GDNF, ROCK, PI3K cat class IA, Calmodulin, CREM (activators), Cyclin A2, HIF1A, IRS-1, SHP-2, GFRalpha2, N-Ras, XIAP, F-Actin cytoskeleton, ATF-1, ERK1/2, FAK1, IKK-gamma, CREB1, VEGFR-1, CDC42, p90RSK2(RPS6KA3), c-Raf-1, SOS, NF-kB, AKT(PKB), SHANK3, ARTN, JNK1(MAPK8), Elk-1, Paxillin, IP3 receptor
**40**	Signal transduction_FAK1 signaling	2.191E-10	8.173E-09	TRIO, RhoA, IL-1 beta, GRP-R, PI3K cat class IA, Rab-21, Calmodulin, ITGA2, PLC-beta, KLF8, MMP-1, ETS, ERK1/2, MMP-14, MMP-2, FAK1, IGF-1 receptor, RAS, JNK(MAPK8-10), c-Raf-1, SOS, AKT(PKB), G-protein alpha-q/11, Collagen IV, PDGF receptor, Fibronectin, MMP-9, Endophilin A2, Collagen I, Paxillin
**41**	Inhibition of Ephrin receptors in colorectal cancer	2.483E-10	8.725E-09	Ephrin-B1, RhoA, RAP-1A, ROCK, c-Rel (NF-kB subunit), Ephrin-B, WNT, TCF7L2 (TCF4), FAK1, Beta-catenin, Ephrin-A receptor 3, CDC42, Ephrin-A receptors,

				Ephrin-A, Ephrin-B2, Ephrin-A receptor 2, Frizzled, Paxillin
**42**	Development_Endothelial differentiation during embryonic development	2.488E-10	8.725E-09	HEY1, TIE2, PI3K cat class IA, VEGF-C, PKC, ETS1, WNT, Neuropilin-1, ERK1/2, Angiopoietin 1, FOXC2, Beta-catenin, HEY2, SMAD1, c-Raf-1, VEGFR-2, Angiopoietin 2, Ihh, AKT(PKB), DLL4, Ephrin-B2, VEGFR-3, FOXF1, GLI-2
**43**	Development_VEGF signaling via VEGFR2 - generic cascades	2.515E-10	8.725E-09	COX-2 (PTGS2), RhoA, p120GAP, PI3K cat class IA, Calcineurin A (catalytic), Calmodulin, PKC, Vinculin, TCF7L2 (TCF4), ROCK1, eIF4E, ERK1/2, eNOS, FAK1, IKK-gamma, CREB1, Beta-catenin, PLAUR (uPAR), CDC42, Actin cytoskeletal, Fyn, c-Raf-1, VEGFR-2, SOS, ERK2 (MAPK1), AKT(PKB), HSP90, MNK1, ERK1 (MAPK3), p90Rsk, PLAU (UPA), MLCK, Paxillin, IP3 receptor
**44**	COVID-19: SARS-CoV-2 effects on the vascular endothelium	2.744E-10	9.303E-09	STAT3, RhoA, IL-1 beta, VE-cadherin, TIE2, ASC/NLRP3, Endothelin-1, CARD5, RIPK1, eNOS, Angiopoietin 1, IKK-gamma, IL-6, CDC42, Thrombomodulin, VEGFR-2, Angiopoietin 2, NF-kB, Tissue factor, NQO1, ADAM17, Coagulation factor VIII, PAI1, TLR2, S1P3 receptor, NALP3
**45**	Apoptosis and survival_NGF/ TrkA PI3K-mediated signaling	4.709E-10	1.561E-08	MLCP (cat), TRIO, BETA-PIX, RhoA, RAP-1A, MLCP (reg), ROCK, PI3K cat class IA, MRCKalpha, AKT1, p70 S6 kinase1, Calcineurin A (catalytic), Calmodulin, Kalirin, MSN (moesin), MRLC, FOXO3A, ERK1/2, KIDINS220, CREB1, CDC42, Actin cytoskeletal, SOS, LIMK2, AKT(PKB), ARAP3, SSH1L, mTOR, PREX1, Tubulin (in microtubules)
**46**	CHDI_Correlations from Discovery data_Causal network	5.313E-10	1.693E-08	PI3K cat class IA, Calmodulin, PLC-beta, PTEN, Kir3.4, WNT, G-protein alpha-i family, TCF7L2 (TCF4), ERK1/2, TSC-22, CREB1, p15, Beta-catenin, G-protein beta/gamma, G-protein alpha-q, c-Raf-1, CCR1, SOS, MEF2, PDGF-R-alpha, AKT(PKB), YY1, Dsh, Elk-1, p90Rsk, Frizzled, IP3 receptor, mTOR, TGF-beta receptor type I
**47**	Signal transduction_S1P1 receptor signaling	5.333E-10	1.693E-08	COX-2 (PTGS2), RhoA, IL-1 beta, VE-cadherin, p70 S6 kinases, PI3K cat class IA (p110-alpha), AKT1, PDGF-B, Calmodulin, PLC-beta, Tcf(Lef), cPLA2, G-protein alpha-i family, Adenylate cyclase, ERK1/2, Cyr61, MMP-14, eNOS, FAK1, PI3K class II (CII-alpha), IL-6, Beta-catenin, G-protein beta/gamma, CDC42, Fyn, Alpha-actinin, JNK(MAPK8-10), c-Raf-1, VEGFR-2, G-protein

				alpha-i3, SOS, CTGF, AKT(PKB), G-protein alpha-i2, Paxillin, IP3 receptor, mTOR, YAP1 (YAp65), PDGF-R-beta
**48**	Cell adhesion_Histamine H1 receptor signaling in the interruption of cell barrier integrity	5.530E-10	1.719E-08	MLCP (cat), Talin, RhoA, Histamine H1 receptor, MLCP (reg), VE-cadherin, ROCK, Calmodulin, PLC-beta, Vinculin, MRLC, FAK1, Beta-catenin, G-protein beta/gamma, Actin cytoskeletal, Alpha-actinin, CPI-17, LIMK2, G-protein alpha-q/11, MLCK, Paxillin, IP3 receptor
**49**	Role of Tissue factor-induced Thrombin signaling in cancer	6.364E-10	1.938E-08	MLCP (cat), RhoA, MLCP (reg), ROCK, PAR3, G-protein alpha-12 family, PI3K cat class IA (p110-alpha), MMP-13, Calmodulin, G-protein alpha-i family, MRLC, ERK1/2, MMP-2, FAK1, Angiopoietin 1, VEGFR-1, Actin cytoskeletal, c-Raf-1, PAR1, ERK2 (MAPK1), AKT(PKB), G-protein alpha-q/11, Tissue factor, ERK1 (MAPK3), MLCK, Paxillin, IP3 receptor
**50**	Immune response_HMGB1/RAGE signaling	6.714E-10	2.004E-08	IL-1 beta, PI3K cat class IA, IL-1 alpha, K-RAS, PLAT (TPA), TLR4, ERK1/2, MEF2C, FAK1, CREB1, IL-6, MEF2A, CDC42, p90RSK2(RPS6KA3), JNK(MAPK8-10), c-Raf-1, IL1RN, NF-kB, AKT(PKB), Tissue factor, Secretogranin II, PAI1, Paxillin, TLR2

Table S_4: Pathway analysis of gene co-expressed EGFR (Pan Cancer Dataset) from the MetaCore database (with p-value< 0.05 set as the cutoff value).

S.No.	Maps	Min FDR	p-value	Network Objects from Active Data
**1**	Development_The role of GDNF ligand family/ RET receptor in cell survival, growth and proliferation	2.688E-07	1.854E-10	STAT3, GDNF, ROCK, PI3K cat class IA, B-Raf, NCK1, c-Fos, GAB1, Cyclin A2, MEKK1(MAP3K1), HIF1A, IKK-alpha, SHP-2, XIAP, ATF-1, EGR1, VEGFR-1, p90RSK2(RPS6KA3), CRK, SOS, c-FLIP, FRS2, AKT(PKB), PDK (PDPK1), ARTN, JNK1(MAPK8)
**2**	Development_EGFR signaling	3.041E-07	4.194E-10	STAT3, EGR2 (Krox20), PI3K cat class IA, Amphiregulin, GSK3 beta, NCK1, SOS2, c-Fos, GAB1, c-Cbl, IKK-alpha, SHP-2, WNT, EGR1, CRK, TGF-alpha, SOS, AKT(PKB), SOS1, EGR3, p120-catenin, Mcl-1, p90Rsk, Betacellulin
**3**	Development_Positive regulation of STK3/4 (Hippo) pathway and negative regulation of YAP/TAZ function	3.233E-07	6.689E-10	Dopamine D1A receptor, LATS1, MPP5, Cullin 2, INADL, Itch, Alpha-1 catenin, FasR(CD95), RASSF5, MARK4, LATS2, Mol1b, Skp2/TrCP/FBXW, TAZ, PKA-reg (cAMP-dependent), MARKK, PEZ, Alpha-catenin, AMOTL2, YAP1 (YAp65), ZO-2, LIF receptor
**4**	Development_Negative regulation of STK3/4 (Hippo) pathway and positive regulation of YAP/TAZ function	6.562E-07	1.810E-09	MLCP (reg), G-protein alpha-12 family, LATS1, PARD3, Itch, LARG, LATS2, Mol1b, TAZ, JNK(MAPK8-10), RASSF6, PAR1, RhoGAP5, G-protein alpha-q/11, PJA2, PDK (PDPK1), MASK, NEDD4, YAP1 (YAp65), ZO-2
**5**	Androgen receptor activation and downstream signaling in Prostate cancer	6.834E-07	2.617E-09	STAT3, Androgen receptor, N-cadherin, gp130, Rb protein, B-Raf, GAB1, c-Cbl, GCR, PTEN, PI3K cat class IA (p110-beta), SHP-2, Versican, IL-6, NCOA1 (SRC1), PAR1, SOS, c-FLIP, FRS2, AKT(PKB), PDK (PDPK1), NCOA2 (GRIP1/TIF2), ADAM10, Elk-4, ADAM17, IL-6 receptor, IL6RA
**6**	DNA damage_Intra S-phase checkpoint	6.834E-07	2.828E-09	HBOA, RAD18, MLL1 (HRX), RIF1, Sirtuin1, BRIP1, SMC3, Histone H3, MRN complex, SMC1, PCAF, ATF-3, HUWE1, ATM, DNA-PK, ASCIZ, Cyclin A, Rad50, Nibrin, TLK1, FBXW11, p53BP1
**7**	G-protein signaling_RhoB activation	7.131E-07	3.443E-09	HGF, KIF13A, p190-RhoGEF, GCR, HIF1A, ATF-2, SMURF1, TGF-beta receptor type II, ATM, LARG, ARHGEF10, JNK(MAPK8-10), PIAS1, AKT(PKB), TIM1, p164-RhoGEF, p300, HGF receptor (Met), TGF-beta receptor type I
**8**	EGFR signaling pathway in lung cancer	8.381E-07	4.624E-09	STAT3, HGF, gp130, PI3K cat class IA, Amphiregulin, c-Fos, PTEN, HIF1A, IKK-alpha, IL-6, EGR1, TGF-alpha,

				SOS, AKT(PKB), PDK (PDPK1), HGF receptor (Met), Mcl-1, ADAM17
**9**	IL-6 signaling in Prostate Cancer	3.087E-06	1.916E-08	STAT3, Androgen receptor, gp130, PI3K cat class IA, GAB1, PTEN, SHP-2, IL-6, NCOA1 (SRC1), SOS, AKT(PKB), PDK (PDPK1), Mcl-1, IL-6 receptor, IL6RA
**10**	Chemotaxis_Lysophosphatidic acid signaling via GPCRs	4.296E-06	2.963E-08	MLCP (reg), ROCK, G-protein alpha-12 family, GSK3 beta, PKC, c-Fos, Vinculin, PI3K cat class IA (p110-beta), ATF-2, DIA1, TRAF6, LARG, ROCK1, FasR(CD95), MKL2, Cyr61, EGR1, N-CoR, TAZ, CRK, JNK(MAPK8-10), MSK1, AKT(PKB), G-protein alpha-q/11, PDK (PDPK1), PKC-epsilon, ADAM17, YAP1 (YAp65)
**11**	Development_Negative regulation of WNT/Beta-catenin signaling in the cytoplasm	7.250E-06	5.500E-08	WWP1, HECTD1, PEG3, Prickle-1, LATS1, c-Cbl, WNT, HUWE1, APC protein, Itch, Alpha-1 catenin, GSK3 alpha/beta, LATS2, WDR26, Skp2/TrCP/FBXW, TAZ, JNK1(MAPK8), A20, YAP1/TAZ, YAP1 (YAp65), G-protein alpha-13
**12**	Development_Positive regulation of WNT/Beta-catenin signaling in the cytoplasm	1.050E-05	8.691E-08	HECTD1, Bcl-9, BIG1, Tankyrases, BIG2, IRS-2, USP9X, USP25, WNT, APC protein, Alpha-1 catenin, GSK3 alpha/beta, PKA-reg type II (cAMP-dependent), JNK(MAPK8-10), AKT(PKB), USP47, SET7, ZBED3, YAP1 (YAp65), DOCK4
**13**	Development_Signaling in embryonic hepatocyte maturation	1.325E-05	1.188E-07	STAT3, HGF, gp130, OSM receptor, PI3K cat class IA, GAB1, HNF1-alpha, FasR(CD95), c-FLIP(Long), HNF6, SOS, OSMR, AKT(PKB), HGF receptor (Met), HNF4-alpha, p120-catenin
**14**	Development_TGF-beta-dependent induction of EMT via RhoA, PI3K and ILK	1.723E-05	1.663E-07	PI3K cat class IA, GSK3 beta, PINCH, SMURF1, IKK-alpha, TGF-beta receptor type II, SLUG, ZO-1, ROCK1, MKL2, Occludin, Caldesmon, AKT(PKB), PDK (PDPK1), TGF-beta receptor type I
**15**	IL-6 signaling in lung cancer	1.893E-05	1.958E-07	STAT3, gp130, PI3K cat class IA, GAB1, HIF1A, ATF-2, IKK-alpha, SHP-2, IL-6, AKT(PKB), PDK (PDPK1), IL-6 receptor, IL6RA
**16**	Regulation and signaling of HGF receptor (Met) and MSP receptor (RON) in lung cancer	1.997E-05	2.204E-07	STAT3, HGF, PI3K cat class IA, GSK3 beta, Rb protein, NCK1, c-Fos, GAB1, c-Cbl, MEKK1(MAP3K1), HIF1A, ATF-2, IL-6, SOS, AKT(PKB), PDK (PDPK1), HGF receptor (Met), p90Rsk, IL-6 receptor
**17**	Immune response_IL-6 signaling via MEK/ERK and PI3K/AKT cascades	2.372E-05	2.781E-07	STAT3, gp130, PI3K cat class IA, GSK3 beta, c-Fos, GAB1, SHP-2, XIAP, IL-6, EGR1, SOS, AKT(PKB), sIL6-RA, PDK (PDPK1), ADAM10, Mcl-1, ADAM17, IL-6 receptor, IL6RA
**18**	Neurophysiological process_Netrin-1 signaling	3.556E-05	4.415E-07	TRIO, ROCK, PI3K cat class IA, MENA, NCK1, VAMP7, ERM proteins, N-WASP, DAPK1, DOCK1, DSCAM,

				Fyn, PKA-reg (cAMP-dependent), VEGFR-2, AKT(PKB), SFK, JNK1(MAPK8), UNC5C
**19**	Development_Regulation of epithelial-to-mesenchymal transition (EMT)	5.292E-05	6.965E-07	HGF, N-cadherin, Arkadia, ATF-2, WNT, TGF-beta receptor type II, SLUG, ZO-1, Sno-N, SIP1 (ZFHX1B), IL-1RI, Occludin, Caldesmon, PDGF-R-alpha, HGF receptor (Met), TCF8, TGF-beta receptor type I
**20**	IGF signaling in hepatocellular carcinoma (HCC)	5.292E-05	7.540E-07	HGF, PI3K cat class IA, Amphiregulin, GSK3 beta, PKC, c-Fos, IRS-2, HIF1A, TGF-alpha, SOS, AKT(PKB), IGF-2 receptor, PDK (PDPK1), HGF receptor (Met), ADAM17
**21**	G-protein signaling_RhoA activation	5.292E-05	7.664E-07	TRIO, N-cadherin, p190-RhoGEF, G-protein alpha-12 family, MUPP1, MPP5, Cyclin B, INADL, SMURF1, ZO-1, Synaptopodin, XIAP, LARG, p190RhoGAP, RHG7, Ephrin-A receptors, PAR1, Collagen IV, Ephrin-A, JNK1(MAPK8)
**22**	HGF signaling in melanoma	5.341E-05	8.368E-07	HGF, N-cadherin, PI3K cat class IA, GSK3 beta, B-Raf, GAB1, HIF1A, ATF-2, SLUG, EGR1, JNK(MAPK8-10), AKT(PKB), HGF receptor (Met)
**23**	TGF-beta signaling via kinase cascades in breast cancer	5.341E-05	8.471E-07	Amphiregulin, ITGAV, c-Fos, ATF-2, IKK-alpha, TGF-beta receptor type II, XIAP, TRAF6, JNK(MAPK8-10), TGF-alpha, AKT(PKB), SOS1, TIMP2, ADAM17, TGF-beta receptor type I, TAB3
**24**	NF-AT signaling in cardiac hypertrophy	5.346E-05	8.848E-07	HDAC9, CBP, gp130, PI3K cat class IA, GSK3 beta, Calcineurin A (catalytic), GAB1, SHP-2, NCX1, IL-6, MEF2A, AKT(PKB), G-protein alpha-q/11, p300, PKC-epsilon, IL-6 receptor, LIF receptor
**25**	Transcription_Hypoxia- and receptor-mediated HIF-1 activation	6.235E-05	1.113E-06	STAT3, HGF, ARNT, CBP, PI3K cat class IA, GSK3 beta, LRRK2, HIF1A, HIF-1, NCOA1 (SRC1), AKT(PKB), p300, PDK (PDPK1), NCOA2 (GRIP1/TIF2)
**26**	DNA damage_ATM-dependent double-strand break foci	6.235E-05	1.118E-06	CBP, HBXAP, Histone H3, MRN complex, BRM, PIAS4, ATM, Histone H2B, BMI-1, RNF168, SMARCA5, PIAS1, HERC2, p300, Bard1, Nibrin, p53BP1
**27**	Development_SLIT-ROBO1 signaling	6.250E-05	1.164E-06	ROCK, PI3K cat class IA, MENA, Calcineurin A (catalytic), NCK1, SLIT1, SLIT2, p190RhoGAP, SLIT3, Fyn, VEGFR-2, Cytohesin3, AKT(PKB)
**28**	Crosstalk between Alzheimer disease and diabetes	6.740E-05	1.301E-06	STAT3, PI3K cat class IA (p110-alpha), GSK3 beta, eIF2AK3, PTEN, IRS-2, PKR, BACE1, IL-6, Fyn, JNK(MAPK8-10), IRE1, AKT(PKB), PDK (PDPK1), IDE
**29**	Immune response_IL-11 signaling via MEK/ERK and PI3K/AKT cascades	6.792E-05	1.405E-06	gp130, PI3K cat class IA (p110-alpha), GSK3 beta, Rb protein, c-Fos, IKK-alpha, SHP-2, ATF-1, IL-6, Fyn, SOS, AKT(PKB), PDK (PDPK1), ADAM10, SFK, p90Rsk, YAP1 (YAp65)

**30**	Signal transduction_Genomic ESR1 and ESR2 signaling	6.792E-05	1.405E-06	CBP, Somatotropin, c-Fos, c-Rel (NF-kB subunit), PCAF, LDLR, SP3, MED1, IL-6, N-CoR, NCOA1 (SRC1), AKT(PKB), p300, IBP1, NCOA2 (GRIP1/TIF2), PR (membrane), PR (nuclear)
**31**	ERBB family and HGF signaling in gastric cancer	7.910E-05	1.691E-06	STAT3, HGF, PI3K cat class IA, Amphiregulin, GSK3 beta, c-Fos, MEKK1(MAP3K1), HIF1A, Neuropilin-1, EGR1, TGF-alpha, SOS, AKT(PKB), PDK (PDPK1), HGF receptor (Met)
**32**	Development_Growth hormone signaling via PI3K/AKT and MAPK cascades	9.298E-05	2.176E-06	PI3K cat class IA (p110-alpha), GSK3 beta, C/EBP zeta, Somatotropin, c-Fos, IRS-2, ATF-2, SHP-2, EGR1, SOS, AKT(PKB), Elk-4, ADAM17
**33**	Development_Canonical TGF-beta signaling	9.298E-05	2.188E-06	ID2, N-cadherin, Arkadia, CBP, Importin (karyopherin)-beta, c-Fos, ETS1, TGF-beta receptor type II, SLUG, Itch, Sno-N, SIP1 (ZFHX1B), Occludin, Karyopherin beta 1, p300, TCF8, TGF-beta receptor type I
**34**	E-cadherin signaling and its regulation in gastric cancer	9.298E-05	2.221E-06	HGF, Formin, GSK3 beta, WNT, SLUG, SIP1 (ZFHX1B), IQGAP1, HAKAI, HGF receptor (Met), p120-catenin, TCF8, Alpha-catenin
**35**	Glucocorticoid-induced elevation of intraocular pressure as glaucoma risk factor	9.298E-05	2.244E-06	TRIO, ROCK, PI3K cat class IA, ROR2, COL4A1, C/EBP zeta, GCR Beta, GCR, PI3K cat class IA (p110-beta), Filamin B (TABP), Myocilin, GCR Alpha, Collagen IV, HGF receptor (Met), PKC-epsilon, MLCK
**36**	Oxidative stress_ROS signaling	9.659E-05	2.398E-06	Sirtuin1, GSK3 beta, PKC, PTEN, MEKK1(MAP3K1), HIF1A, IKK-alpha, ATM, DLC1 (Dynein LC8a), IRP2, GRP75, IL-6, EGR1, PKA-reg (cAMP-dependent), JNK(MAPK8-10), SAE2, AKT(PKB), p300, FASN, NRF2, JNK1(MAPK8), ADAM17
**37**	Immune response_Oncostatin M signaling via MAPK	1.194E-04	3.087E-06	gp130, OSM receptor, c-Fos, MEKK1(MAP3K1), LDLR, SHP-2, LIFR, EGR1, JNK(MAPK8-10), SOS, OSMR, LIF receptor
**38**	Signal transduction_TrkB signaling	1.194E-04	3.282E-06	Bcl-W, CBP, PI3K cat class IA, Rab-5A, MAP3K2 (MEKK2), PKC, c-Fos, GAB1, IRS-2, SHP-2, Myosin Va, MKP-1, MEF2A, p90RSK2(RPS6KA3), JNK(MAPK8-10), SOS, SLITRK5, AKT(PKB), p300
**39**	Development_Stimulation of differentiation of mouse embryonic fibroblasts into adipocytes by extracellular factors	1.194E-04	3.344E-06	CBP, EGR2 (Krox20), PI3K cat class IA (p110-alpha), HIVEP2, BMP receptor 2, IRS-2, ATF-2, SHP-2, XIAP, ATF-1, BMPR1A, p90RSK2(RPS6KA3), SMAD1, PKA-reg (cAMP-dependent), SOS, PDK (PDPK1), LIF receptor

**40**	TGF-beta 1-induced transactivation of membrane receptors signaling in hepatocellular carcinoma (HCC)	1.194E-04	3.377E-06	PI3K cat class IA, GSK3 beta, ITGA2, PTEN, TGF-beta receptor type II, SLUG, DOCK1, Cyclin A, CRK, PDGF-R-alpha, AKT(PKB), PDGF receptor, DDX5, TGF-beta receptor type I
**41**	Survival pathways in Prostate Cancer	1.194E-04	3.377E-06	STAT3, Androgen receptor, PI3K cat class IA, GSK3 beta, PTEN, IKK-alpha, XIAP, IL-6, c-FLIP(Long), AKT(PKB), DR4(TNFRSF10A), PDK (PDPK1), Mcl-1, IL-6 receptor
**42**	Transcription_Epigenetic regulation of gene expression	1.230E-04	3.563E-06	HBOA, HDAC9, CBP, MLL1 (HRX), Sirtuin1, Histone H3, MOZ, PCAF, UTX, Histone H2B, MORF, JMJD1A, p300, AOF1, SET7
**43**	Cholesterol dysregulation in Alzheimer disease	1.385E-04	4.107E-06	Sirtuin1, c-Fos, eIF2AK3, BACE1, VLDLR, NRSF, LDLR, HMDH, LAMP2, Fyn, ABCA1, NPC1, SYNJ1, NRF2, NR2A, CYP7B1, LRP1
**44**	IL-6 signaling in multiple myeloma	1.416E-04	4.369E-06	STAT3, gp130, PI3K cat class IA, GSK3 beta, Rb protein, SHP-2, IL-6, VEGFR-2, SOS, AKT(PKB), PDK (PDPK1), Mcl-1, IL-6 receptor, IL6RA
**45**	Ovarian cancer (main signaling cascades)	1.416E-04	4.395E-06	HGF, Androgen receptor, PI3K cat class IA (p110-alpha), PI3K cat class IA, GSK3 beta, NCOA4 (ARA70), B-Raf, c-Fos, PTEN, MEKK1(MAP3K1), IKK-alpha, IL-6, PKA-reg (cAMP-dependent), SOS, AKT(PKB), HGF receptor (Met)
**46**	Development_Androgen receptor in reproductive system development	1.418E-04	4.499E-06	AHR, Androgen receptor, N-cadherin, CBP, PI3K cat class IA, Amphiregulin, CYP19, Occludin, ARID4B, NCOA1 (SRC1), EEA1, SOS, PICALM, AKT(PKB), PDK (PDPK1), NCOA2 (GRIP1/TIF2), ARID4A, ZO-2
**47**	Immune response_IFN-alpha/beta signaling via PI3K and NF-kB pathways	1.736E-04	5.628E-06	STAT3, SLFN5, PI3K cat class IA, GSK3 beta, Rb protein, IFIT1, EMSY, IRS-2, IKK-alpha, ISG54, eIF4B, p130, Cyclin A, c-FLIP, eIF4G1/3, AKT(PKB), PDK (PDPK1), p107, PKC-epsilon, PD-L1
**48**	Signal transduction_IGF-1 receptor signaling	1.940E-04	6.421E-06	STAT3, Androgen receptor, PI3K cat class IA, GSK3 beta, Calcineurin A (catalytic), GAB1, IRS-2, PCAF, SHP-2, HMDH, EGR1, SOS, AKT(PKB), p300, PDK (PDPK1), FASN, NRF2, PKC-epsilon, JNK1(MAPK8), p90Rsk, LRP1
**49**	Development_Role of growth factors in the maintenance of embryonic stem cell pluripotency	2.036E-04	7.160E-06	ROCK, PI3K cat class IA, GSK3 beta, c-Fos, GAB1, Activin A, SHP-2, TGF-beta receptor type II, ROCK1, SOS, FRS2, AKT(PKB), PDK (PDPK1), TGF-beta receptor type I
**50**	IL-6 signaling in breast cancer cells	2.036E-04	7.160E-06	STAT3, N-cadherin, gp130, PI3K cat class IA, c-Fos, GAB1, SHP-2, CYP19, IL-6, SOS, AKT(PKB), Mcl-1, IL-6 receptor, IL6RA

Table S_5: Pathway analysis of gene co-expressed PD-L1 (Pan Cancer) from the MetaCore database (with p-value< 0.05 set as the cutoff value).

S.No.	Maps	pValue	Min FDR	Network Objects from Active Data
**1**	COVID-19: immune dysregulation	5.164E-19	7.576E-16	HGF, IL-1 beta, GATA-3, alpha-L/beta-2 integrin, MIG, HLA-DRB1, MHC class II, IRAK4, sIL2RA, CCR7, IP10, MyD88, CD3, IL-10, FOXP3, CCL8, Granzyme B, CCR2, FasR(CD95), CD4, IL-6, HLA-DPA1, MIP-1-beta, STAT5, GATA Group, HLA-DRA1, CCR5, TLR7, IL1RN, TLR8, NF-kB, CSF1, MIP-1-alpha, Caspase-1, TLR3, HLA-DMB, ITGAL, Granzyme A, Btk, HLA-DPB1, Perforin
**2**	Immune response_CTLA-4 signaling	9.541E-17	6.998E-14	RAP-1A, alpha-L/beta-2 integrin, IDO1, CD80, PI3K cat class IA, AP-1, Hck, PKC-eta, Lck, JunB, NF-AT1(NFATC2), Eomesodermin, ZAP70, CD3, FOXP3, TXK, Lyn, CD3 zeta, Granzyme B, PAK2, Itch, CD28, CBL-B, CTLA-4, Fyn, FasL(TNFSF6), NF-kB, C3G, ALPHA-PIX, CD86, NF-AT
**3**	Chemokines in inflammation in adipose tissue and liver in obesity, type 2 diabetes and metabolic syndrome	3.279E-16	1.603E-13	ITGAX, IL-1 beta, Fc gamma RI, MHC class II, VCAM1, CD45, CD14, ITGA4, LYVE-1, CD44, CMKLR1, CD3, PSGL-1, TLR4, CCR2, IL-6, MANR, FCGR3A, CCR5, EMR1, MIP-1-alpha, ITGAL, CD86, CXCR4, CD163, CD1c
**4**	CHDI_Correlations from Replication data_Causal network (positive correlations)	7.478E-16	2.743E-13	Pyk2(FAK2), ROCK, CD80, MHC class II, PI3K cat class IA, Calcineurin A (catalytic), Lck, CD45, Slp76, NF-AT1(NFATC2), CD44, MyD88, ZAP70, CD3, ITK, TRAF6, IL-1RI, CD28, IP3R1, G-protein beta/gamma, MSK1/2 (RPS6KA5/4), IRAK1/2, JNK(MAPK8-10), MEF2, NF-kB, HSP70, CaMK IV, PI3K reg class IB (p101), CXCR4, PI3K cat class IB (p110-gamma), IP3 receptor, MEKK4(MAP3K4), VIL2 (ezrin)
**5**	B-regulatory cells and tumor cells intercellular interaction	1.550E-15	4.547E-13	FPRL1, IL-1 beta, TNF-R2, JAK2, gp130, CD80, GM-CSF receptor, EBI3, IL-10, VDR, IL-35, TLR4, Granzyme B, GM-CSF, CCR2, FasR(CD95), IL-1RI, CD28, IL-6, JNK(MAPK8-10), VEGFR-2, TLR7, FasL(TNFSF6), NF-kB, CD5, Bcl-6, IL-12 alpha, CD86, TGF-beta receptor type I, IL-12RB2
**6**	Chemotaxis_SDF-1/ CXCR4-induced chemotaxis of immune cells	5.946E-15	1.454E-12	BETA-PIX, Talin, RAP-1A, Pyk2(FAK2), alpha-L/beta-2 integrin, JAK2, PI3K cat class IA, VCAM1, PAK, Lck, CD45, NCK1, PLC-beta, ITGB2, Vinculin, ZAP70, CD3, CD3 zeta, ITK, G-protein alpha-i family, PAK2, ROCK1, G-protein beta/gamma, WASP, Fyn, PKA-reg (cAMP-dependent), SFK, PI3K reg class IB (p101), Btk, CXCR4, PI3K cat class IB (p110-gamma), Cofilin

**7**	Breakdown of CD4+ T cell peripheral tolerance in type 1 diabetes mellitus	7.856E-15	1.646E-12	HLA-DRB1, CD80, MHC class II, IL-12 receptor, AP-1, Lck, ZAP70, CD3, FOXP3, T-bet, CD4, CD28, STAT5, CTLA-4, HLA-DQB1, PTPN22, NF-kB, c-FLIP, IL-23 receptor, ICOS, Bcl-6, HLA-DQA1, IL-2R alpha chain, CD86, NF-AT
**8**	Rheumatoid arthritis (general schema)	1.568E-13	2.876E-11	IL-1 beta, Fc gamma RI, alpha-L/beta-2 integrin, TNF-R2, HLA-DRB1, CD80, MHC class II, VCAM1, FOXP3, HLA-DRB, MMP-1, CD2, TLR4, CD4, CD28, IL-6, FCGR3A, FGF2, PTPN22, CSF1, MHC class II beta chain, IL-2R alpha chain, CD86, BAFF(TNFSF13B)
**9**	Mast cell migration in asthma	2.038E-13	3.322E-11	MIG, ROCK, CCL21, PI3K reg (p87-gamma), CCR7, IP10, CD44, I-TAC, TGF-beta receptor type II, CXCR6, G-protein alpha-i family, CCR2, P2Y1, G-protein beta/gamma, VEGFR-1, FGF2, VEGFR-2, CCR1, LTBR2, MGF, MIP-1-alpha, Adenosine A3 receptor, PI3K reg class IB (p101), CCR4, C3aR, CXCR4, CXCR3, PI3K cat class IB (p110-gamma), TGF-beta receptor type I
**10**	Immune response_IL-12 signaling	3.087E-13	4.529E-11	JAK2, MLL1 (HRX), c-IAP2, PI3K cat class IA, IL-12 receptor, MMP-13, Lck, NF-kB1 (p50), RUNX3, Eomesodermin, CD3, IL-10, MMP-1, T-bet, CD3 zeta, Granzyme B, STAT5, Fyn, NF-kB, ICOS, Bcl-6, PDK (PDPK1), IL-2R alpha chain, IL-12 alpha, IL-12RB1, Perforin, BLIMP1 (PRDI-BF1), IL-12RB2, MEKK4(MAP3K4)
**11**	Macrophage and dendritic cell phenotype shift in cancer	5.059E-13	6.747E-11	NF-kB p50/p50, COX-2 (PTGS2), PGE2R2, IL-1 beta, IFNGR1, PGE2R4, IDO1, CD80, MHC class II, IL-12 receptor, GM-CSF receptor, IP10, c-Rel (NF-kB subunit), IL-10, MSR1, M-CSF receptor, TLR4, GM-CSF, TRAF6, SOCS3, IL-1RI, IL-6, EPAS1, PERC, TLR7, NF-kB, MER, CSF1, IRF4, ILT4, IL-12 alpha, CD86, ILT3, TGF-beta receptor type I
**12**	Dengue virus infection mechanism	1.525E-12	1.864E-10	Syk, IL-1 beta, Fc gamma RI, IRp60, PI3K cat class IA, ILT2, DAP12, Calcineurin A (catalytic), CD14, TRAF3, CD44, IL-10, Fc epsilon RI gamma, UFO, ITGB3, APG5, TRAF6, Itch, SOCS3, CD209, TBK1, MDL-1, MANR, FCGR3A, TLR7, Fc gamma RII alpha, HSP70, Caspase-1, PDK (PDPK1), TLR3, DC-SIGNR, PKA-cat (cAMP-dependent), NALP3
**13**	Immune response_Immunological synapse formation	2.133E-12	2.407E-10	Talin, RAP-1A, alpha-L/beta-2 integrin, CD80, MHC class II, PI3K cat class IA, VCAM1, Slp76, NCK1, ITGB2, STK4, URP2, Vinculin, ZAP70, CD3, ITK, CD28, WASP, Fyn, FYB1, C3G, Cytohesin1, CD86, WaspIP

**14**	Immune response_Antigen presentation by MHC class II	2.940E-12	3.081E-10	MHC class II alpha chain, Syk, Cathepsin L, Dectin-1, MHC class II, PI3K cat class IA, ORP1, Dynein 1, cytoplasmic, heavy chain, MARCH1, HSC70, PKC, LLIR, c-Cbl, MyD88, Kinesin heavy chain, Fc epsilon RI gamma, HCLS1, CLEC9A, TLR4, Itch, CD209, CD4, SWAP-70, Fc alpha receptor, LY75, Fc gamma RII beta, HLA-DO, MANR, FCGR3A, JNK(MAPK8-10), PLEKHM2, Legumain, PI3K cat class III (Vps34), MHC class II beta chain, PDK (PDPK1), Cathepsin V
**15**	Chemotaxis_Lysophosphatidic acid signaling via GPCRs	3.187E-12	3.117E-10	Tiam1, MLCP (reg), ROCK, PRKD1, cPKC (conventional), G-protein alpha-12 family, AP-1, PAK, LPAR3, PKC, c-Fos, PLC-beta, Vinculin, DIA1, Tcf(Lef), HAS2, LPAR6, EGFR, LPAR5, G-protein alpha-i family, TRAF6, ROCK1, FasR(CD95), Cyr61, HB-EGF, G-protein beta/gamma, Rho GTPase, IL13RA2, JNK(MAPK8-10), MSK1, G-protein alpha-q/11, PDK (PDPK1), PLC-epsilon, Cofilin, IP3 receptor, YAP1 (YAp65), G-protein gamma 12, PREX1
**16**	Cell adhesion_Integrin inside-out signaling in neutrophils	1.401E-11	1.285E-09	Syk, Talin, RAP-1A, Fc gamma RI, alpha-L/beta-2 integrin, DAP12, Talin-1, Hck, Slp76, ITGB2, CD44, URP2, PSGL-1, Lyn, G-protein alpha-i family, FGR, G-protein beta/gamma, FYB1, C3G, PDK (PDPK1), Cytohesin1, PI3K reg class IB (p101), Btk, FPR, PI3K cat class IB (p110-gamma), IP3 receptor, PREX1, PLC-beta2
**17**	Immune response_TLR2 and TLR4 signaling	1.506E-11	1.299E-09	COX-2 (PTGS2), IL-1 beta, TLR1, PI3K cat class IA, AP-1, Hck, Pellino 1, IRAK4, CD14, NF-kB1 (p105), MyD88, TLR10, IL-10, TLR6, Lyn, TLR4, TRAF6, MD-2, IRAK2, IL-6, MSK1/2 (RPS6KA5/4), JNK(MAPK8-10), NF-kB, TAB2, PDK (PDPK1), p90Rsk
**18**	Role of microglia in Alzheimer disease	1.986E-11	1.618E-09	Syk, COX-2 (PTGS2), FPRL1, IL-1 beta, PI3K cat class IA, DAP12, CD14, IL10RA, MyD88, IL-10, C1q, CD33, MSR1, TLR6, Lyn, TLR4, DAP10, IL-6, C1qa, MIP-1-beta, Fyn, NF-kB, PI3K cat class III (Vps34), MIP-1-alpha, Caspase-1, PDK (PDPK1), FPR, NALP3
**19**	Immune response_Glucocorticoid receptor immunological signaling	2.207E-11	1.704E-09	COX-2 (PTGS2), IL-1 beta, GATA-3, MIG, PI3K cat class IA, AP-1, IL7RA, TIGIT, IP10, GCR, IL-10, T-bet, GM-CSF, MKP-1, eNOS, CIITA, IL-6, MIP-1-beta, JNK(MAPK8-10), HLA-DRA1, DSIPI (GILZ), PDK (PDPK1), NCOA2 (GRIP1/TIF2), NF-AT, CXCR4, BLIMP1 (PRDI-BF1)
**20**	B cell signaling in hematological malignancies	4.323E-11	3.169E-09	Syk, Cyclin D2, JAK2, IRAK4, PKC-beta1, CD20, TRAF3, c-Cbl, MyD88, ZAP70, CD38, Lyn, TRAF6, CD70(TNFSF7), Semaphorin 4D, BCMA(TNFRSF17), CD27(TNFRSF7), IL-6, Bcl-10, NF-kB, Bcl-6, PDK (PDPK1), Btk, PKC-beta2, PI3K cat class IA (p110-delta), IP3 receptor, BAFF(TNFSF13B)

**21**	Immune response_Antigen presentation by MHC class I: cross-presentation	4.537E-11	3.169E-09	Syk, Cathepsin L, CLEC12A, Dectin-1, Fc gamma RI, Rab-27A, DAP12, IRAK4, IRAP, LLIR, MyD88, C1q, Fc epsilon RI gamma, UFO, MSR1, cPLA2, CLEC9A, TLR4, gp91-phox, CD209, LY75, HSP105, MANR, FCGR3A, TLR7, Fc gamma RII alpha, HSP70, Rab8B, TLR3, Rab-33A, p47-phox
**22**	Chemotaxis_Common mechanisms of Th17 cell migration	4.836E-11	3.225E-09	alpha-L/beta-2 integrin, MIG, VCAM1, IP10, PLC-beta, CD43, I-TAC, PSGL-1, CCL8, CXCR6, G-protein alpha-i family, CCR2, MIP-1-beta, G-protein beta/gamma, CCR5, MIP-1-alpha, PI3K reg class IB (p101), CCR4, CXCR3, PI3K cat class IB (p110-gamma), IP3 receptor
**23**	Cytokines/chemokines in spinal neuronal-glial interactions driving neuropathic pain	5.167E-11	3.296E-09	COX-2 (PTGS2), PGE2R2, IL-1 beta, TNF-R2, JAK2, ROCK, IL-18R1, PI3K cat class IA, DAP12, CCL21, PKC, IP10, c-Fos, PLC-beta, cPLA2, G-protein alpha-i family, M-CSF receptor, TRAF6, CCR2, CXCL13, IL-1RI, IL-6, P2Y12, NF-kB, CSF1, IL-23 receptor, P2Y6, JNK1(MAPK8), PKA-cat (cAMP-dependent), p90Rsk, CXCR4, CXCR3, PI3K cat class IB (p110-gamma)
**24**	Immune response_Inhibitory PD-1 signaling in T cells	5.925E-11	3.622E-09	Syk, GATA-3, alpha-L/beta-2 integrin, CD80, MHC class II, PI3K cat class IA, PD-L2, Lck, PTEN, Eomesodermin, ZAP70, CD3, FOXP3, T-bet, CD3 zeta, CD4, CD28, CBL-B, Skp2/TrCP/FBXW, FYB1, PDK (PDPK1), CD86
**25**	Immunological synapse between dendritic and CD8+ T cells in allergic contact dermatitis	7.164E-11	4.204E-09	alpha-L/beta-2 integrin, CD80, IL-12 receptor, AP-1, PD-L2, Lck, NF-AT1(NFATC2), CD2, CD70(TNFSF7), CD27(TNFRSF7), CD28, CTLA-4, NF-kB, CD137(TNFRSF9), ICOS, CD86, RANK(TNFRSF11A)
**26**	Th17 cells in cystic fibrosis (mouse model)	7.802E-11	4.303E-09	IL-1 beta, JAK2, CD80, MHC class II, CD14, MyD88, FOXP3, TGF-beta receptor type II, TLR4, GM-CSF, CD4, MD-2, IL-1RI, CD28, IL-6, NF-kB, IL-23 receptor, IRF4, SARA, CD86, TGF-beta receptor type I
**27**	SLE genetic marker-specific pathways in T cells	8.051E-11	4.303E-09	Calcineurin A (alpha), HLA-DRB1, TNIP1, MHC class II, IL-12 receptor, Calcineurin A (catalytic), Lck, Slp76, CalDAG-GEFIII, RUNX3, NF-AT1(NFATC2), ZAP70, CD3, IL-10, HLA-DRB, ETS1, T-bet, Aiolos, CD3 zeta, IP3R1, HLA-DRB3, DNMT1, PTPN22, SOS, NF-kB, IL-23 receptor, MHC class II beta chain, KLRK1 (NKG2D), A20, MICB, Lnk
**28**	Immune response_M-CSF-receptor signaling	8.527E-11	4.303E-09	PU.1, Syk, Cyclin D2, Pyk2(FAK2), p120GAP, PI3K cat class IA, AP-1, DAP12, Hck, MAP3K2 (MEKK2), PKC, c-Fos, c-Cbl, Tcf(Lef), MSR1, ETS1, M-CSF receptor, WASP, Fyn, NF-kB,

				CSF1, SOS1, PDK (PDPK1), GAB3, STAT5B, PLAU (UPA), IP3 receptor
**29**	Signal transduction_S1P3 receptor signaling	8.781E-11	4.303E-09	COX-2 (PTGS2), Tiam1, Pyk2(FAK2), JAK2, ROCK, G-protein alpha-12 family, PI3K cat class IA, AP-1, VCAM1, c-Fos, PLC-beta, G-protein alpha-12, TGF-beta receptor type II, EGFR, G-protein alpha-i family, Adenylate cyclase, ROCK1, MEF2C, eNOS, IL-6, G-protein beta/gamma, JNK(MAPK8-10), MSK1, VEGFR-2, PA24A, SOS, PDK (PDPK1), CXCR4, PI3K cat class IB (p110-gamma), Cofilin, IP3 receptor, TGF-beta receptor type I, YAP1 (YAp65)
**30**	T follicular helper cell dysfunction in SLE	8.800E-11	4.303E-09	RC3H2, IFNGR1, TRIM, CD80, MHC class II, NTB-A, PI3K cat class IA, IL-12 receptor, IL-7, CD4, CXCL13, CD84, CD28, IL-6, SAP, CTLA-4, Fyn, Bcl-10, SLAM, TLR7, NF-kB, IL-23 receptor, ICOS, Bcl-6, IRF4, A20, CD86, BLIMP1 (PRDI-BF1), BAFF(TNFSF13B)
**31**	Role of Bregs in attenuation of T and NK cells mediated anti-tumor immune responses	1.185E-10	5.608E-09	MIG, CD80, Lck, IP10, CD3, CD1d, IL-10, FOXP3, TGF-beta receptor type II, CD3 zeta, Granzyme B, FasR(CD95), CD28, CTLA-4, FasL(TNFSF6), IL-2R alpha chain, CD86, CXCR3, TGF-beta receptor type I
**32**	Immune response_T cell co-signaling receptors, schema	1.416E-10	6.491E-09	PP2135, CD30L (TNFSF8), CD80, MHC class II, NTB-A, TIGIT, PD-L2, LAIR1, CD244, CD30(TNFRSF8), CD2, CD70(TNFSF7), GITRL, CD27(TNFRSF7), CD28, CTLA-4, SLAM, CD137(TNFRSF9), BTLA, ICOS, CD48, CD86
**33**	Immune response_Fc epsilon RI pathway: Lyn-mediated cytokine production	1.917E-10	8.522E-09	Syk, IL-1 beta, PKC-beta, AP-1, Calcineurin A (catalytic), PKC-beta1, Slp76, MAP3K2 (MEKK2), PKC, CCL1, c-Fos, NF-AT1(NFATC2), Fc epsilon RI gamma, Lyn, TRAF6, Fer, MEF2C, IL-6, BFL1, JNK(MAPK8-10), Bcl-10, SOS, Fc epsilon RI beta, NF-kB, FYB1, Btk, NF-AT, IP3 receptor
**34**	Signal transduction_Mu-type opioid receptor signaling in non-neuronal cells	2.223E-10	9.590E-09	IL-1 beta, cPKC (conventional), AP-1, Calcineurin A (catalytic), Lck, Metenkefalin, c-Fos, NF-AT1(NFATC2), EGFR, G-protein alpha-i family, HB-EGF, G-protein beta/gamma, PKA-reg (cAMP-dependent), JNK(MAPK8-10), CCR1, SOS, PDK (PDPK1), PI3K reg class IB (p101), LPP3, PKA-cat (cAMP-dependent), NF-AT, PI3K cat class IB (p110-gamma), IP3 receptor
**35**	Immune response_Th17, Th22 and Th9	3.992E-10	1.673E-08	PU.1, AHR, IL-1 beta, GATA-3, CD80, MHC class II, AP-1, ROR-alpha, NF-AT1(NFATC2), IL-10, TGF-beta receptor type

	cell differentiation			II, CD4, CD28, IL-6, NF-kB, IRF4, CD86, TGF-beta receptor type I
**36**	Aberrant production of IL-2 and IL-17 in SLE T cells	4.792E-10	1.953E-08	Syk, IL-1 beta, MHC class II, NTB-A, AP-1, Lck, CREM (repressors), c-Fos, NF-AT1(NFATC2), ZAP70, CD3, Fc epsilon RI gamma, ETS1, CD3 zeta, CD4, IL-6, SAP, PKA-reg (cAMP-dependent), SOS, CD3 epsilon, CaMK IV, PKA-cat (cAMP-dependent)
**37**	Immune response_HSP60 and HSP70/ TLR signaling	6.922E-10	2.672E-08	CD69, IL-1 beta, CD80, MHC class II, AP-1, IRAK4, CD14, NF-kB1 (p105), MyD88, IL-10, TLR4, TRAF6, MD-2, IL-6, IRAK1/2, JNK(MAPK8-10), NF-kB, TAB2, HSP70, IL-12 alpha, CD86
**38**	Th17 cells in cystic fibrosis	6.922E-10	2.672E-08	IL-1 beta, JAK2, CD80, MHC class II, VCAM1, CD14, ROR-alpha, MyD88, TGF-beta receptor type II, TLR4, GM-CSF, CD4, MD-2, IL-1RI, CD28, IL-6, NF-kB, IL-23 receptor, IRF4, CD86, TGF-beta receptor type I
**39**	Role of tumor-infiltrating B cells in anti-tumor immunity	8.210E-10	3.088E-08	MIG, IL-18R1, MHC class II, IL-12 receptor, IP10, CD20, XAGE1, CD38, I-TAC, RGS13, T-bet, G-protein alpha-i family, Granzyme B, FasR(CD95), CD4, CXCL13, CD27(TNFRSF7), G-protein beta/gamma, FasL(TNFSF6), NF-kB, Bcl-6, IRF4, RGS1, Btk, Perforin, CXCR4, CXCR3, BLIMP1 (PRDI-BF1)
**40**	Immune response_IFN-gamma in macrophages activation	9.774E-10	3.584E-08	PU.1, COX-2 (PTGS2), Fc gamma RI, CYP27B1, JAK2, MIG, IP10, c-Rel (NF-kB subunit), IL-10, TLR4, gp91-phox, IL-6, MER, C1qb, Caspase-1, IL-12 alpha, CLIC5, Pleiotrophin (OSF1), C1qc, RANK(TNFRSF11A)
**41**	Immune response_Naive CD4+ T cell differentiation	1.342E-09	4.800E-08	PU.1, AHR, IL-1 beta, GATA-3, CD80, DEC2, MHC class II, AP-1, ROR-alpha, RUNX3, NF-AT1(NFATC2), IL-10, T-bet, CD4, CD28, IL-6, NF-kB, IRF4, CD86
**42**	Role of endothelial and immune cells in systemic sclerosis	1.478E-09	4.833E-08	Cathepsin L, GATA-3, gp130, VCAM1, MyD88, MMP-1, TGF-beta receptor type II, TLR4, CCR2, Neuropilin-1, FasR(CD95), Cyr61, LIFR, MMP-12, EPAS1, SMAD1, TLR8, FLI1, Cathepsin V, PI3K cat class IA (p110-delta), TGF-beta receptor type I, YAP1 (YAp65)
**43**	Signal transduction_MIF signaling	1.478E-09	4.833E-08	PU.1, Syk, COX-2 (PTGS2), PI3K cat class IA, AP-1, VCAM1, PLC-beta, CD44, ZAP70, cPLA2, Lyn, G-protein alpha-i family, TLR4, G-protein beta/gamma, JNK(MAPK8-10), MSK1, NF-kB, PDK (PDPK1), SFK, PI3K reg class IB (p101), CXCR4, PI3K cat class IB (p110-gamma)
**44**	Immune response_Fc epsilon RI pathway: signaling	1.478E-09	4.833E-08	Syk, SGK3, AP-1, PAG, c-Cbl, Fc epsilon RI gamma, Lyn, ZFP36(Tristetraprolin), SYTL3, FGR, CCL3L1, PI3K class II (CII-alpha), STAT5, SGK1, Fyn, Fc epsilon RI beta, NF-kB,

	through Fyn and PI3K			FYB1, PDK (PDPK1), STAT5B, NF-AT, PI3K cat class IA (p110-delta)
**45**	Immune response_NF-AT in immune response	1.483E-09	4.833E-08	Syk, TRIM, CD80, MHC class II, PI3K cat class IA, Calcineurin A (catalytic), Lck, Slp76, NF-AT1(NFATC2), ZAP70, CD3, Lyn, CD3 zeta, ITK, CD28, NF-kB, Btk, CD86, NF-AT, IP3 receptor
**46**	Chemotaxis_CCL19- and CCL21-mediated chemotaxis	1.530E-09	4.879E-08	PGE2R2, Pyk2(FAK2), PGE2R4, ROCK, CCL21, CCR7, c-Fos, G-protein alpha-i family, Adenylate cyclase, G-protein beta/gamma, PKA-reg (cAMP-dependent), JNK(MAPK8-10), PKA-cat (cAMP-dependent), Cofilin, IP3 receptor, PLC-beta2
**47**	Immune response_CD40 signaling in dendritic cells, monocytes, and macrophages	1.981E-09	6.184E-08	Syk, COX-2 (PTGS2), PGE2R2, IL-1 beta, IDO1, c-IAP2, CD80, MHC class II, PI3K cat class IA (p110-alpha), PI3K cat class IA, PKC-beta1, TRAF3, IL-10, Lyn, NF-kB p50/c-Rel, TRAF6, IL-1RI, IL-6, JNK(MAPK8-10), SOS, NF-kB, JNK1(MAPK8), IL-12 alpha, PKC-beta2, CD86
**48**	T regulatory cell migration in asthma	2.221E-09	6.650E-08	IL-16 mature, PI3K cat class IA (p110-alpha), Lck, CCL1, CCR8, G-protein alpha-i family, CD4, G-protein beta/gamma, CCL18, PDK (PDPK1), PI3K reg class IB (p101), CCR4, PI3K cat class IB (p110-gamma)
**49**	Cell adhesion_Integrin inside-out signaling in T cells	2.221E-09	6.650E-08	Talin, RAP-1A, alpha-L/beta-2 integrin, JAK2, VCAM1, Lck, CCL21, CCR7, ITGB2, STK4, URP2, Vinculin, ZAP70, CD3, CD3 zeta, G-protein alpha-i family, G-protein beta/gamma, FYB1, CXCR4, PLC-beta2
**50**	Th2 cell migration in asthma	2.666E-09	7.670E-08	IL-16 mature, alpha-L/beta-2 integrin, PI3K cat class IA (p110-alpha), VCAM1, Lck, CCL1, CD44, CCR8, G-protein alpha-i family, CD4, G-protein beta/gamma, Beta-2 adrenergic receptor, CCL18, PI3K reg class IB (p101), CCR4, PI3K cat class IB (p110-gamma)
